# Comparative Study of Functionalized Carbosilane Dendrimers for siRNA Delivery: Synthesis, Cytotoxicity, and Biophysical Properties

**DOI:** 10.1021/acsomega.4c08314

**Published:** 2024-12-20

**Authors:** Monika Müllerová, Piotr Tarach, Tomáš Strašák, Petra Cuřínová, Roman Petrickovic, Táňa Závodná, Jan Topinka, Anna Janaszewska, Barbara Klajnert-Maculewicz, Lucie Červenková Št’astná

**Affiliations:** a Institute of Chemical Process Fundamentals Czech Academy of Sciences, Rozvojová 135, Prague 165 02, Czech Republic; b Faculty of Biology and Environmental Protection, Department of General Biophysics, University of Lodz, Pomorska 141/143, Lodz 90-236, Poland; c Institute of Experimental Medicine, Czech Academy of Sciences, Vídeňská 1083, Prague 142 00, Czech Republic; d Department of Organic Chemistry, University of Chemistry and Technology Prague, Technická 5, Prague 6 166 28, Czech Republic

## Abstract

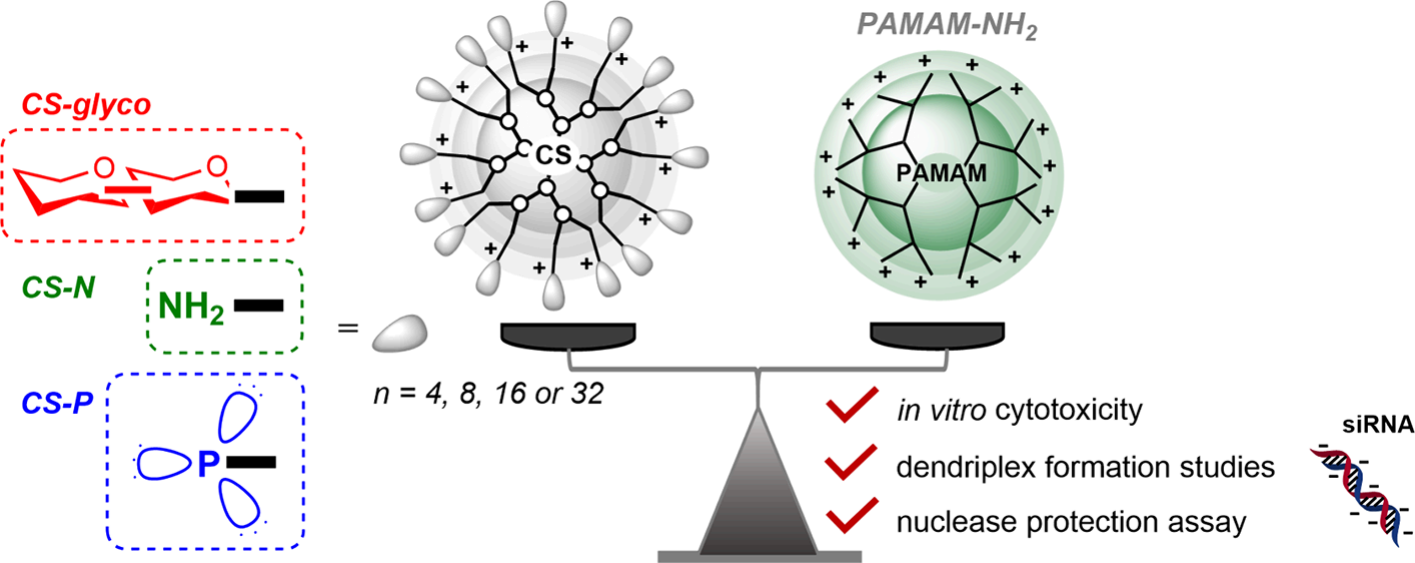

Efficient and safe carriers of genetic material are crucial for advancing gene therapy. Three new series of cationic dendritic nanocarriers based on a carbosilane scaffold, differentiated by peripheral modifications: saccharide (CS-glyco), amine (CS-N), and phosphonium dendrimers (CS-P) were designed for binding, protecting, and releasing polyanionic compounds like therapeutic siRNA. Besides introducing synthetic methodology, this study brings a unique direct interstructural comparison of 16 dendritic nanovector’s characteristics, addressing a gap in typical research that focuses on uniform structural types. The study evaluates the dendrimer’s *in vitro* cytotoxicity, biophysical properties, and complexation capabilities in comparison with widely used PAMAM dendrimers. CS-glyco and PAMAMs were significantly less toxic to MCF-7 and THP-1 cell lines than were CS-N and CS-P, despite having the same peripheral charge density. Notably, CS-glyco maintained biocompatibility comparable to analogous neutral CS glycodendrimers, underscoring the exceptional capability of sugar coating to reduce toxicity. Dendriplexes formed from these nanocarriers protected siRNA from RNase degradation and facilitated its release in the presence of heparin, highlighting its potential in gene delivery applications. The study provides a background for future in-depth investigations into the introduced dendritic nanocarriers, which show significant potential for advancing drug delivery.

## 1 Introduction

The rapidly evolving field of gene therapy holds significant promise for the treatment of various fatal genetic-based diseases.^1^ At the heart of this approach is the precise local delivery of NAs, i.e., DNA and small interfering RNA (siRNA), into target cells to exert a therapeutic effect on specific genes.^2^ Advances in gene delivery depend on the development of safe and effective transport vehicles. While virus-based vectors still raise eligible concerns regarding mutagenicity, oncogenesis, and host immune responses, rapid progress in nonviral delivery platforms promises highly efficient and safe synthetic alternative.^3^ Investigations into lipid-, peptide-, and polymer-based transport systems, inorganic nanoparticles, and carbon-based nanomaterials proved this concept.^4,5^ Compared to the above, dendrimers (DDMs) have an undeniable advantage in topological and structural fine-tunability, narrow molecular weight distribution, and a highly functionalizable surface.^6^

As for other nanoparticles, DDMs’ ability to condense nucleic acids (NAs) relies on the attractive electrostatic interactions between the negatively charged phosphate groups in the NA backbone and the positively charged groups in the DDM. This results in dendriplexes forming, often demonstrating improved transfection efficiency and lower toxicity than linear or branched polymers.^7,8^ The DDMs’ complexation ability is influenced by the DDM to NA ratio and the prevailing bulk conditions, such as pH or ionic strength.^9^ The complexation of genetic material enhances its bioavailability and stability, protects it from degradation by endogenous nucleases, and facilitates its delivery.^10^ Moreover, the DDMs’ size and surface charge can be tailored to optimize their interaction with biological systems, i.e., to enhance cellular uptake, prolong circulation time in the body, or target specific cells or tissues. In summary, a well-designed dendritic vector can significantly improve the efficacy of gene therapy and reduce potential side effects.^11^ While DDMs offer significant advantages as drug delivery carriers, there are still challenges that need addressing.

DDMs with polyamine structural features have been vastly studied as nonimmunogenic transporters of genetic material with minimal nonspecific blood–protein binding.^12^ The relationship between the size and surface functionalization of polyamidoamine DDMs (PAMAMs) directly influences their biodistribution and toxicity.^13^ Compared with polyethylenimine (PEI) DDMs, PAMAMs are generally more biocompatible and have higher NA loading capacity.^14^ In addition, the proton sponge effect of PAMAM DDMs helps endosomal escape, which is a critical step for augmenting transfection efficiency.^12^ However, a primary concern in PAMAMs is the size constraint: PAMAMs up to generation 5 can be effectively cleared through glomerular filtration in the renal excretion pathway. In contrast, hepatic clearance pathways are responsible for the elimination of PAMAMs of generation 6 and above.^15^ DDMs sized between 4 and 10 nm possess the capability to engage with nanoscale cellular components and can surpass the cellular endocytosis barrier.^16^ Nevertheless, due to their elevated costs and pronounced toxicity, PAMAM DDMs of generation 6 and beyond are infrequently employed; thus, other structural alternatives are sought for. A convenient approach to reduce dendritic carrier toxicity is to exploit dendritic frameworks with an inert internal structure. Carbosilane (CS) DDMs bearing ammonium^17,18^ and phosphonium^19^ peripheral groups were systematically investigated in drug and gene delivery. In our previous study, we showed lower toxicity of phosphonium-terminated CS-DDMs compared to their ammonium analogs and demonstrated that smart choice of ligands attached to the phosphorus atom can further decrease the toxicity of the compounds.^19^ Consecutively, we showed that CS-DDMs with PMe_3_ groups at the periphery are low-toxic transfection vectors for siRNA cell delivery.^20^ Krashenina et al.^21^ investigated the formation of complexes between anticancer siRNAs and ammonium-terminated CS-DDMs and their interactions with HeLa and HL-60 cancer cells, showing high promises for siRNA delivery into tumor tissue. Zawadzki et al.^22^ identified ammonium-terminated CS-DDMs decorated with PEG chains potentially capable of safe delivery of genetic materials across the blood–brain barrier.

Cationic sugar-decorated polymeric and dendritic compounds were investigated for targeted gene delivery.^23^ The conjugation of saccharides enhances the solubility, stability, and biocompatibility of cationic DDMs, which are known to exhibit significant cytotoxicity.^6,7^ For instance, galactose-modified bifunctional PAMAM or PPI constructs have demonstrated selective siRNA/DNA delivery into HepG2 cells.^24^ Han et al. prepared glucose-decorated lysine dendrons with aliphatic chains with reduced toxicity and high transfection efficiency in HeLa and HepG2 cells.^25^

The ideal delivery vehicle for siRNA should effectively shield it from degradation, efficiently transport it to specific cells or tissues, enable its successful transfection into the cytoplasm, and achieve these objectives with minimal toxicity to healthy tissues, thereby avoiding adverse effects.^2^ While the majority of research focuses on dendritic nanovectors of the same structural type, there is a notable deficiency in studies that evaluate dendritic carriers from an interstructural perspective.^26^ This gap in research complicates the comparison between dendritic vectors composed of different structural elements.

In this report, we detail a synthetic method and comprehensive characterization of three novel types of cationic dendritic nanocarriers based on CS scaffolds, each modified with different peripheral units: sugar (CS-glyco), CS-N, and phosphonium (CS-P). This study aims to examine the biological and biophysical attributes of these newly developed dendritic vectors in comparison to the extensively used PAMAMs, to evaluate their potential for biomedical applications, and to lay the groundwork for further studies. The evaluation involved *in vitro* biocompatibility testing using MCF-7 and THP cell lines, along with fundamental biophysical assessments such as nanoparticle size and zeta potential measurements. Additionally, the formation and stability of dendriplexes was demonstrated by zeta potential profiles and gel retardation assay. Finally, the effectiveness of the dendritic carriers in protecting genetic material from degradation by endogenous nucleases was shown through gel retardation assay.

## 2 Materials and Methods

### 2.1 Synthesis and Characterization of the DDMs

Allyl-terminated (CS_0_-allyl, CS_1_-allyl, CS_2_-allyl) and 3-iodopropyl-terminated (CS_1_-I, CS_2_-I, CS_3_-I) CS-DDMs were prepared according to literature and our previously reported procedures.^19,27^ Unless otherwise stated, dimethylformamide (DMF), MeI, diisopropylethylamine (DIPEA), 2,2-dimethoxy-2-phenylacetophenone (DMPA), methyl-thioglycolate, tris(2,4,6-trimethoxyphenyl)phosphine (TTMP), tris(4-methoxy-3,5-dimethylphenyl)phosphine, and Amberlite IRA402 were purchased from commercial sources and used without further purification. Organic solvent nanofiltration (OSN) was performed according to the previously reported procedure.^28^

NMR spectra were obtained on a Bruker Avance 400 (^1^H at 400.1 MHz; ^13^C{^1^H} at 100.6 MHz; ^29^Si {^1^H} at 79.5 MHz) at 25 °C. ^1^H and ^13^C NMR signals of the compounds were assigned to corresponding atoms using gHSQC, gCOSY, gHMBC, and gHSQC TOCSY 2D NMR correlation spectra. ^1^H and ^13^C chemical shifts (δ/ppm) are given relative to residual solvent signals (δ_H_/δ_C_: DMSO-*d*_6_, 2.50/39.52, CDCl_3_, 7.26/77.16, acetonitrile-*d*_3_, 2.13/118.26); ^29^Si spectra were referenced to external standard hexamethyldisilane (−19.87 ppm). ^31^P spectra were referenced to external standard triphenylphosphine oxide (−17.00 ppm). HRMS spectra were obtained using a MicroTOF-QIII instrument (Bruker Daltonics, Germany) with an ESI or APCI ionization source in positive mode.

Hereinbelow, representative abbreviations were used to refer to certain characteristics or structural motives of the compounds: *G_n_* refers to the generation of the DDM, where *n* = 1, 2, or 3; IPh refers to **6** units^28^ in the DDM structure.

#### 2.1.1 CS-glyco: General Synthetic Procedure

The CS glyco DDMs **1a**–**3a** prepared according to the previously reported procedure^29^ were dissolved in dry DMF (0.5 mL per 0.1 g of the DDM), and MeI was added (0.1 mL per 0.1 g of the DDM). The solution was transferred to a microwave reaction vial (10 mL), sealed with a septum, placed into the microwave reactor cavity, and irradiated up to 55 °C with stirring (600 rpm) for 3 h. Completion of the quaternization was checked using ^1^H NMR, and the procedure was repeated when necessary. After cooling down to 20 °C, the vial content was concentrated under reduced pressure, yielding compounds **1b**–**3b** as yellowish-brown powders (95–97%). Then, these derivatives with iodide counterion **1b**–**3b** were converted to chloride derivatives **1c**–**3c** by ion exchange on AMBERLYST A-21 resin (water/MeOH 1:1). The products were obtained in quantitative yields.

#### 2.1.2 CS-N: General Synthetic Procedures

##### 2.1.2.1 Compound **4**

To the solution of tetraallylsilane (CS_0_allyl; 1 equiv) in dry DMF (10 mL) was added a catalytic amount of DMPA. The solution was degassed with argon stream (5 min), then methyl-thioglycolate (1.1 eq. per reactive site) was added. The reaction mixture was irradiated by a UV light (400 W mercury lamp, thin-wall vials; PYREX filter; 30 min) under vigorous stirring. The solvent and volatiles were evaporated under vacuum. The crude reaction mixture was dissolved in diethyl ether and extracted with 1% NaOH (3×), water (2×), and saline (1×). The organic phase was dried with MgSO_4_, filtered, and concentrated under vacuum. The crude product was purified using gradient column chromatography (PE/EtOAc 1:4 → 1:2) yielding **4** as the vicious yellowish liquid (87%).

##### 2.1.2.2 Compounds **5** and **6**

To the solution of allyl-terminated DDM (CS_1_allyl, CS_2_allyl; 1 equiv) in dry DMF (10 mL), a catalytic amount of DMPA was added. The solution was degassed with argon stream (5 min), and then methyl-thioglycolate (1.1 equiv per reactive site) was added. The reaction mixture was irradiated by UV light (400 W mercury lamp, thin-wall vials; PYREX filter; 30 min) under vigorous stirring. The solvent and volatiles were evaporated under a vacuum. The crude reaction mixture was dissolved in diethyl ether and extracted with 1% NaOH (3×), water (2×), and saline (1×). The organic phase was dried with MgSO_4_, filtered, and concentrated under vacuum. The crude product was purified using OSN using DCM/MeOH (1:2) yielding vicious transparent liquid (83–86%).

##### 2.1.2.3 Compound **7**

The access amount (10 mL) of ethylenediamine was added to ester **4** under an argon atmosphere. The reaction mixture was heated (70 °C) under vigorous stirring for 12 h. The access of ethylenediamine was evaporated under vacuum to obtain a vicious brownish liquid (98%).

##### 2.1.2.4 Compounds **8** and **9**

The access amount (10 mL) of ethylenediamine was added to ester **5** or **6** under an argon atmosphere. The reaction mixture was heated (70 °C) under vigorous stirring for 12 h. The access of ethylenediamine was evaporated under vacuum, and the crude product was purified using OSN (DCM/MeOH 1:2) to obtain a vicious brownish liquid (94–96%).

#### 2.1.3 CS-P: General Synthetic Procedure

Reactions were carried out on a 0.15–0.30 mmol scale.

##### 2.1.3.1 Compounds **10a**–**15b**

Respective phosphines TTMP or TDMP (1.2 equiv per reactive site) were added to a solution of CS_1_-I, CS_2_-I, or CS_3_-I (1 equiv) in acetonitrile (25 mL). The reaction mixture was heated (80 °C) under vigorous stirring for 48 h. Then, the solvent was removed under vacuum. Toluene (30 mL) was added to the crude product, and the suspension was refluxed (1 h). After cooling to rt, the toluene phase was removed and the procedure was repeated (3×) to separate the access of the phosphine reagent. The products **10a**–**15a** were obtained as a brownish powder (88–93%). Then, these derivatives with iodide counterion **10a**–**15a** were converted to chloride derivatives **10b**–**15b** by ion exchange on an AMBERLYST A-21 resin (MeOH liquid phase). The products were obtained in quantitative yields.

### 2.2 DDM-siRNA Calculations

The dendrimer/siRNA ratio was calculated according to the following formula:
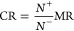
where CR is the charge ratio, MR is the molar ratio (DDM/siRNA), *N*^+^ denotes the number of cations per DDM, and *N*^–^ denotes the number of anions per siRNA molecule.

### 2.3 Zeta Potential Measurements

The zeta potential of the nanoparticles was determined using a Zetasizer Nano ZS instrument (Malvern Instruments Ltd., UK) at 25 °C in Malvern disposable folded capillary cells (800 μL sample volume). For the zeta potential measurements of DDMs, 10 μM DDM solutions were prepared in a Na-phosphate buffer (pH 7.4). The zeta potential for each sample was ascertained as the mean value from 10 to 15 individual recordings with each assessment being replicated three times.

For dendriplex zeta potential measurements, the siRNA/DDM complexes were formed by mixing respective volumes of siRNA (0.2 μM) and DDM solutions (Na-phosphate buffer, 10 mmol/L, pH 7.4) at concentrations providing the desired MR (16:1 to 1:16) in a total volume of 0.7 mL. The mixtures were incubated for 5 min and gently vortexed before measurements. The zeta potential for each sample was ascertained as the mean value from 10 to 15 individual recordings, with each assessment being replicated three times.

### 2.4 DLS Measurements of Hydrodynamic Diameter

The hydrodynamic diameter of the DDMs and/or their aggregates was measured by DLS employing a Zetasizer Nano ZS (Malvern Instruments Ltd., UK) at 25 °C in Malvern disposable plastic microcuvettes (100 μL sample volume). For the preparation of samples, commercial sterile PBS was used to create 10 μM solutions of the DDMs. The light scattered at 173° from the incident light was fitted to an autocorrelation function, adopting the method of cumulants (Malvern Instruments Ltd., UK). Before the measurements, the samples were vortexed to ensure uniformity. The hydrodynamic diameters were determined from five independent repetitions (each 10–50 runs). Multimodal intensity-weighted particle size distribution was used for data analysis.

### 2.5 Gel Retardation Electrophoresis and Nuclease Protection Assay

Agarose gel electrophoresis served as the analytical technique to examine the complexation between the DDMs and siRNA. This is predicated on the principle that the positively charged DDMs impede the migration of the negatively charged siRNA when subjected to an electric field.^30^ Additionally, this electrophoretic method was employed to ascertain whether the DDMs confer protective benefits to siRNA against degradation by ribonucleases.

For the assessment of dendriplex stability, complexes of control siRNA (Dharmacon Inc.; Lafayette, CO, USA, 0.5 μM) and DDM in a range of DDM/siRNA MRs (Figure 3) were prepared in 10 mM phosphate buffer (pH 7.4) and incubated for 30 min at room temperature. Then, the samples were placed on a 3% agarose gel containing GelRed stain (Biotium, Fremont, CA, USA) and separated by electrophoresis in a Tris-acetate-EDTA (TAE) buffer for 45 min at 90 V/35 mA. The gel was subsequently visualized using UV light, and a digital picture of the stained gel was taken with an Azure 300 Imagining System (Azure Biosystems, Dublin, CA, USA).

An analogous methodology was applied to perform the nuclease protection assay. The DDM/siRNA complexes were constituted at the optimal ratio, as determined by gel retardation electrophoresis. This ratio was identified as the point at which the entire quantity of genetic material was complexed by the dendrimer (Table 2, gel electrophoresis, MR_sat_). The complexes were prepared in 10 mM phosphate buffer (pH 7.4) and incubated for 30 min at room temperature. Then, the samples were incubated with RNase A/T1 (Thermo Fisher Scientific, Waltham, MA, USA, 0.25 μg/mL) for 15 min at 37 °C. Then, RNAsin (Eastport Lifescience, 1 μL) was added to each sample to ensure RNase deactivation prior to putting the sample on ice for 10 min. Heparin (Sigma-Aldrich, St. Louis, MO, USA, 0.082 mg/mL) was added to the samples for 3 min to release siRNA from the complexes. Then, the samples were placed on 3% agarose gel containing GelRed stain (Biotium, Fremont, CA, USA) and separated by electrophoresis in a TAE buffer for 45 min at 90 V/35 mA. The gel was subsequently visualized, and a digital picture was obtained as described above.

### 2.6 Cytotoxicity of the DDMs

Cytotoxicity of the DDMs and DDM complexes was tested on cancer (MCF-7 and THP1) human cell lines in a concentration range of 0.5–25 μM by using a resazurin assay. MCF-7 (human breast adenocarcinoma, HTB-2) and THP1 (monocyte human leukemia, ATCC TIB-202) cell lines were purchased from the American Type Culture Collection (ATCC, Rockville, USA). MCF-7 cells were grown as monolayers in DMEM growth medium supplemented with fetal bovine serum (10%), penicillin (10 U/mL), and streptomycin (50 μg/mL). THP1 cells were grown in a suspension in an RPMI-1640 medium (Gibco, Thermo Fisher Scientific, Waltham, MA, USA) supplemented with fetal bovine serum (10%), penicillin (10 U/mL), and streptomycin (50 μg/mL; Sigma-Aldrich, Taufkirchen, Germany). Cells were grown at 37 °C in 5% CO_2_. The cells were subcultured 24 h before the treatment to ensure that they were in the exponential growth phase. MCF-7 cells were trypsinized, suspended in fresh medium, seeded into 96-well plates containing 1.5 × 10^4^ cells/well, and incubated for 24, 48, or 72 h at 37 °C in a humidified atmosphere containing 5% CO_2_. THP1 cells were centrifuged at 1300 × *g* for 5 min, suspended in a new medium, and seeded into plates at optimal densities. After 24 h, selected concentrations of DDMs were added to plates for 24, 48, or 72 h. After the incubation, resazurin was added to the culture medium to a final concentration of 10 μg/mL. The plates were incubated at 37 °C in darkness to allow for the conversion of resazurin to resorufin. The fluorescence of the metabolized resazurin in the cell suspension was measured after 3 h at 530 nm excitation and 590 nm emission using a PowerWave HT Microplate Spectrophotometer (BioTek, USA). Data were presented as a percentage of viability of control (untreated) cells.

### 2.7 Data Evaluation and Statistics

Statistical analysis of zeta potential and DLS measurements and cell viability data were performed using the GraphPad software (La Jolla, CA). Results were expressed as the mean ± standard deviation (SD). IC_50_ values were estimated by the GraphPad program using nonlinear regression.

## 3 Results and Discussion

### 3.1 Synthesis and Characterization of the DDMs

**Scheme 1:**
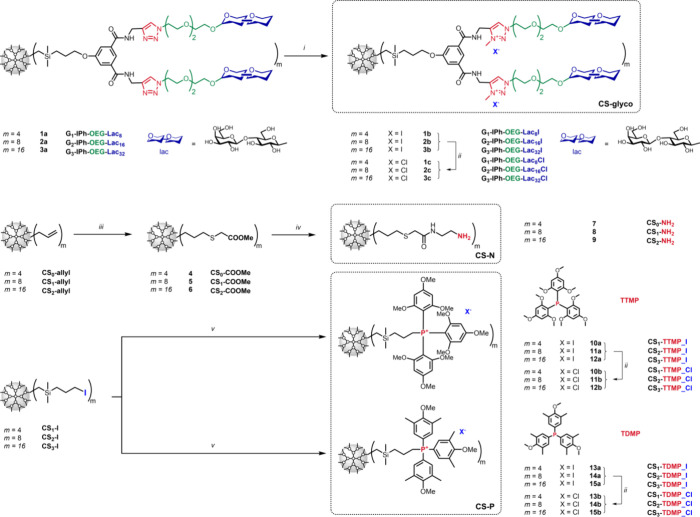
Synthesis of Dendritic Nanocarriers—CS-glyco, CS-N and CS-P; (i) MeI, DMF, MW, 50°C, (ii) AMBERLYST A-21, water/MeOH 1:1, (iii) Methyl-Thioglycolate, DMPA, DMF, MW, (iv) Ethylenediamine, 70 °C, (v) TTMP or TDMP, Acetonitrile, 80 °C

Lactose-decorated CS-DDMs **1a**–**3a** were prepared following the previously reported robust synthetic protocol.^29^ This method facilitates peripheral attachment of saccharide moieties in a multivalent manner via copper(I)-catalyzed azide–alkyne cycloaddition (CuAAC). The microwave-assisted reaction setup is known to accelerate the reaction progress^31−33^ and proved to be generally beneficial in the synthesis of our CS glycoDDMs in different synthetic steps including CuAAC,^28^ de-*O*-acetyletion,^29^ and *N*-methylation. To achieve positively charged glycoDDMs **1b**–**3b** (Scheme 1), the formation of the triazole ring through CuAAC was followed by quaternization via *N*-methylation. The formation of the uniform singlet of the *N*-methyl group (4.39–4.69 ppm) and the shift of the indicative singlet of triazole proton (7.94 pm) downfield to 8.94–8.98 ppm in ^1^H NMR reliably indicated quantitative quaternization of the triazole ring of the glycoDDMs. Notably, quaternization with MeI was unsuccessful at normal temperatures in the absence of MW irradiation. We assume that an elevated temperature is required to accelerate the reaction, but the low boiling point of MeI prevents the heating of the reaction outside the MW reactor cuvettes. Finally, we substituted iodide anions with chloride anions using an ion-exchange resin, yielding glycoDDMs **1c**–**3c.**

The CS-N, composed of a CS interior (0th–second generation CS) core and a polyamidoamine outer layer, was synthesized from basic allyl-terminated CS-DDMs (CS_0–2_-allyl). DDMs **4**–**6** were generated via a photoinitiated thiol–ene reaction between the CS_0–2_-allyl DDMs and methyl-thioglycolate, used in slight excess. The quantitative addition of thioglycolate to the CS_0–2_-allyl DDMs was reliably indicated by the emergence of a uniform singlet of the methylene group adjacent to the sulfur atom (approximately 3.3 ppm) and the disappearance of the double-bond signals (two multiplets at cca 5.4 and 6.0 ppm) in ^1^H NMR spectra. The reaction mixture required processing under inert conditions to minimize the formation of disulfide bridges from excess methyl-thioglycolate and careful evaporation of solvents and excess methyl-thioglycolate below 40 °C to avoid thermal degradation of the products. The separation of the cross-linked methyl-thioglycolate from the crude DDMs was imperative to prevent it from further reacting in subsequent synthetic stages, which could lead to the formation of undesirable, virtually inseparable macromolecular byproducts. DDMs **5** and **6** were purified using OSN, while DDM **4**, with its lower molecular weight, required column chromatography for purification.

The amino-terminated series of DDMs **7**–**9** were synthesized from DDMs **4**–**6** through aminolysis with an excess of ethylenediamine, which also functioned as the solvent. Complete conversion, achieved typically overnight, was indicated by the disappearance of the methyl singlet (approximately 3.6 ppm) and the emergence of signals corresponding to the methylenes of the −CH_2_CH_2_NH_2_ group (2.57–3.14 ppm) in ^1^H NMR spectra. Similarly to previous processes, the complete removal of the residual ethylenediamine was critical to avoid byproduct formation in later synthetic steps. The evaporation of ethylenediamine required careful temperature control due to the thermal sensitivity of the products, with degradation observed starting from 50 °C.

The synthesis of the CS-P DDM series was conducted utilizing our well-established protocol for the incorporation of onium salts onto the periphery of DDMs.^19^ This involved a quaternization process where 3-iodopropyl-terminated DDMs (CS_1–3_-I) were reacted with a slight excess of the selected phosphines (TTMP or TDMP). In accordance with previous observations, the initially hydrophobic DDMs, insoluble in acetonitrile, progressively attained solubility as the peripheral onium group count increased, effectively transitioning the compounds into a soluble state. Upon completion of this reaction, surplus phosphine was removed using toluene extraction. In line with the behavior of previously synthesized analogs, the resulting DDMs **10a**–**15a** having I^–^ counterion were insoluble in water. To address this issue, we performed a counterion exchange in the concluding step, substituting iodide ions for chloride anions, following a procedure akin to that used for the glycoDDMs mentioned earlier. The final products **10b**–**15b** were acquired as off-white solids. The NMR spectroscopic and analytical data corresponded to the proposed structures, indicating the presence of the Si(CH_2_)_3_P unit by a doublet of outer silicon atoms in ^29^Si{^1^H} NMR (typical coupling constant ^4^*J*_(Si–P)_ 2.2–3.2 Hz) and phosphorus signals in ^31^P {1H} NMR (cca 5.2 ppm for compounds **10b**–**12b** and 3.4 ppm for **13b**–**15b**). In the case of CS-P as quaternary phosphonium salts, it is noteworthy that the ions were readily detectable through electrospray ionization, primarily due to the generation of multiple-charged species. This characteristic facilitated simple monitoring of counterions in the positive mode, thereby providing a reliable method to confirm the completion of the iodide/chloride ion-exchange process. Full structures are provided in SI (Scheme SI 1).

### 3.2 Cytotoxicity

**Figure 1:**
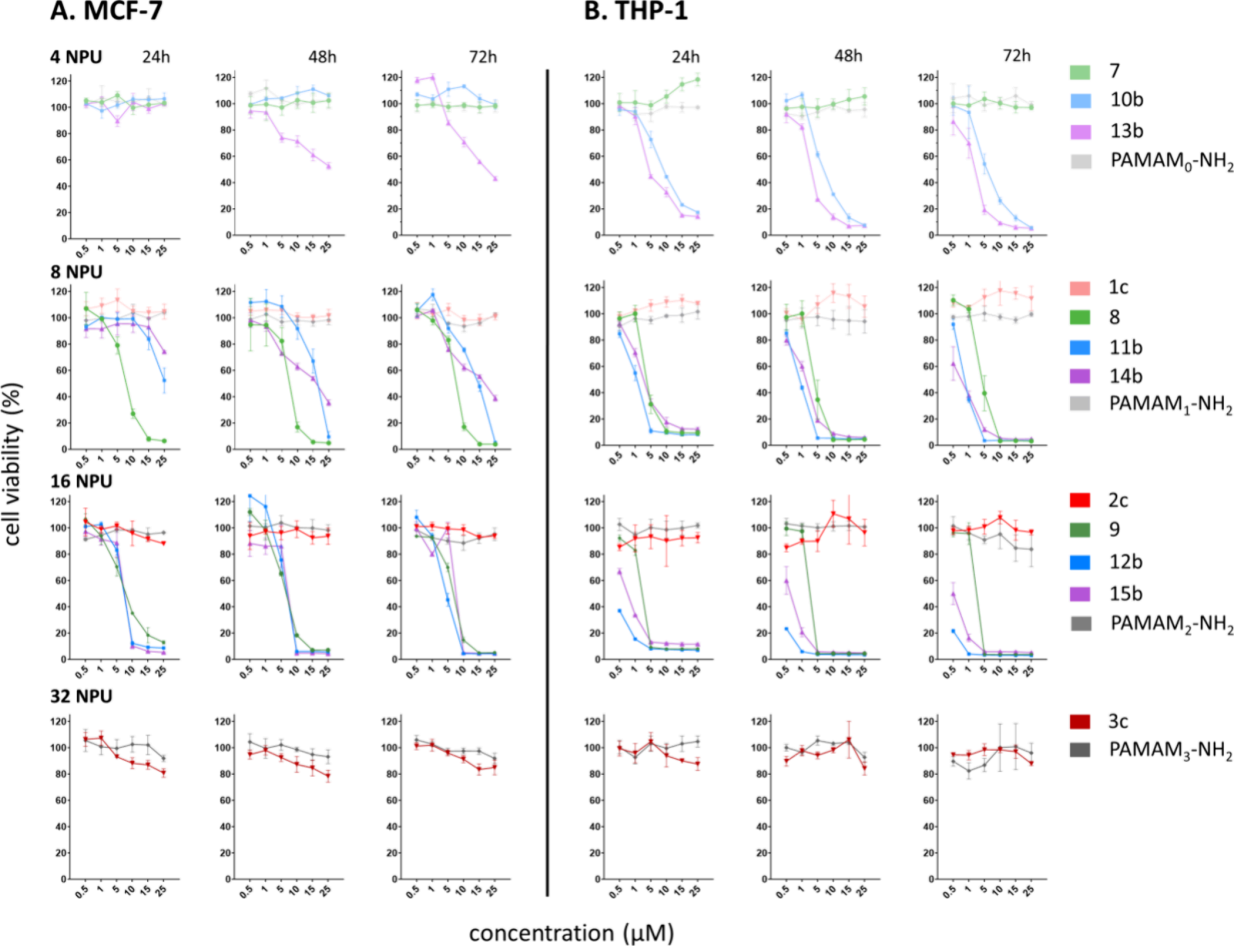
Cytotoxicity comparison of four different structural types of DDMs with resembling NPUs to MCF-7 (A) and THP1 (B) cancer cell lines. The cytotoxicity was measured after 24, 48, and 72 h in the 0.5–25 μM concentration range. Data are presented as mean ± SD.

The biocompatibility or possible anticancer properties of the DDMs are associated with their structural composition; generation; and most importantly, with the amount and type of positively charged functional groups.^8^ Therefore, the dendritic nanovectors (CS-glyco, CS-N, and CS-P), which differ in their chemical composition, were evaluated and contrasted according to their number of peripheral units (NPUs). In the tested concentration range, the dendritic nanocarriers exhibited notable variations in their toxicity toward cancer cells, depending on their generation and structural design (Figure 1).

To obtain CS-glyco, we incorporated charged triazolium motifs through the quaternization of triazole rings using a methylation agent. Since this approach to integrating inherently charged groups within glycoDDM frameworks has not been reported before, the implications of these charged motifs on the functional aspects of glycoDDMs remain unexplored and cannot be directly compared to existing systems. Yet, previous research has consistently shown the high compatibility of glycoDDMs with biological systems.^34−38^ For example, maltose-modified open-shell and dense-shell poly(propyleneimine) DDMs exhibited IC_50_ of 100–145 μM when tested on the SKOV3 cell line.^39^ In a similar vein, Wrobel et al. demonstrated a significant hemolytic activity for maltose-functionalized hyperbranched poly(ethylene imine)s, with the activity initiating at concentrations of 100–300 μM.^40^ Recently, we have reported exceptional biocompatibility of neutral CS glycoDDMs.^28,41^ In this study, the THP-1 human monocytic cell line was selected due to its relevance in studying immune responses, particularly its ability to model monocyte/macrophage functions and assess the potential immunogenicity of nanocarriers.^42^ This is critical for evaluating whether our dendritic carriers could trigger undesirable immune reactions during gene therapy applications. On the other hand, the MCF-7 breast cancer cell line was chosen as it serves as a standard model for assessing the antitumor activity and cytotoxicity of drug delivery systems.^43^ Given that one of the potential applications of these DDMs is targeted cancer therapy via siRNA delivery, MCF-7 allows us to investigate their efficacy in a tumor environment while also providing insights into their interaction with cancer cells.

For CS-glyco, the cell viability was above 85% up to 25 μM concentration in both THP1 and MCF-7 cell lines, indicating the absence of a toxic effect. This is a particularly favorable outcome as generally, the presence of charged moieties at the DDM’s periphery contributes most significantly to the compound’s cytotoxicity. The presence of charged triazolium moieties in the CS-glyco did not decrease cell viability either with increasing generation or with increasing time exposure. The effect of CS-glyco on the tested cells’ viability was steadily stable across DDM generations with negligible difference in viability after 24, 48, and 72 h of exposure. Therefore, despite being charged, CS-glyco bearing up to 32 charged functional moieties possess resembling biocompatibility with analogous neutral drug delivery systems (DDSs) like the glucose- and galactose-decorated CS-DDMs we reported earlier.^28^ From the aforementioned, it can be assumed that the presence of a biocompatible saccharide peripheral layer significantly decreases the cytotoxic effect of the charged DDM periphery (triazolium moieties), while the complexation capability is retained (see Sec 3.4). An additional factor could be that the positive charge of the system is likely partially shielded within the interior of the DDM scaffold, which may further enhance its biocompatibility. This phenomenon has been observed in other dendritic systems, where the internal shielding of positive charge similarly contributes to improved biocompatibility.^6^ In our study, comparable cell viabilities were obtained for PAMAM DDMs with the same number of NPU, where elevated toxicity started to be observed from fourth generation (64 NPU). This is in alignment with reported cytotoxicity results for PAMAMs. Shao et al.^44^ demonstrated that PAMAM_3_-NH_2_ is not toxic at concentrations up to 72 μM. Zhang et al.^45^ reported the viabilities of MCF-7/ADR cells treated with PAMAM_4_-NH_2_ after 48 and 72 h to be more than 80% up to 50 μM concentration.

Contrarily, the viability of both cancer cell lines upon exposure to CS-P and CS-N was strongly concentration- and generation-dependent, and these DDMs showed significantly higher toxicity toward the cell lines tested than both CS-glyco and PAMAMs with equivalent NPU.

Except for virtually nontoxic G1, the CS-N was similarly toxic to both tested cell lines regardless of the exposure time. With their IC_50_ from 7 to 2 μM (Table 1), this was in strong contrast with the nontoxic PAMAM DDMs of the same NPU (PAMAM_1_-NH_2_ and PAMAM_2_-NH_2_). A possible explanation for this outcome might be the electrostatic repulsion between the polar polyamine exterior and the hydrophobic CS core, unlike that in the PAMAM structure. This repulsion prevents effective packing, leaving all amine-terminated branches, which are protonated^12^ under physiological conditions, exposed on the surface. While this makes the CS-N reactive with biological materials, it simultaneously increases their toxicity.

**Table 1:** IC_50_ Values of the Tested DDMs.^a^

IC_50_ (μM)							
NPU	Cell line	MCF-7			THP-1		
	Time (h)	24	48	72	24	48	72
4	CS_0_-NH_2_ (7)	>100	>100	>100	>100	>100	>100
	CS_1_–P-TTMP-Cl (10)	>100	>100	>100	7.8 ± 0.8	5.7 ± 1.0	4.5 ± 0.7
	CS_1_–P-TDMP-Cl (13)	>100	23.5 ± 1.8	22.8 ± 4.2	4.5 ± 0.4	2.5 ± 0.3	1.8 ± 0.2
	PAMAM_0_-NH_2_	>100	>100	>100	>100	>100	>100
							
8	G_1_-IPh-OEG-Lac_8_Cl (1c)	>100	>100	>100	>100	>100	>100
	CS_1_–NH_2_ (8)	6.0 ± 1.4	5.2 ± 1.4	5.4 ± 1.5	3.2 ± 0.6	3.0 ± 0.7	3.4 ± 1.0
	CS_2_–P-TTMP-Cl (11)	54.1 ± 13.7	24.5 ± 8.1	15.3 ± 3.8	1.3 ± 0.2	1.0 ± 0.2	1.0 ± 0.2
	CS_2_–P-TDMP-Cl. (14)	>100	15.6 ± 0.7	17.4 ± 1.2	2.6 ± 0.2	1.5 ± 0.1	0.7 ± 0.1
	PAMAM_1_-NH_2_	>100	>100	>100	>100	>100	>100
							
16	G_2_-IPh-OEG-Lac_8_Cl (2c)	>100	>100	>100	>100	>100	>100
	CS_2_–NH_2_ (9)	6.6 ± 1.0	4.9 ± 1.1	4.4 ± 1.0	2.0 ± 0.4	2.2 ± 0.6	2.1 ± 0.6
	CS_3_–P-TTMP-Cl (12)	5.6 ± 1.5	5.5 ± 2.0	3.3 ± 0.8	0.3 ± 0.1	0.1 ± 0.1	0.1 ± 0.1
	CS_3_–P-TDMP-Cl (15)	5.2 ± 1.5	4.5 ± 1.4	5.2 ± 1.8	0.8 ± 0.1	0.5 ± 0.1	0.4 ± 0.1
	PAMAM_2_-NH_2_	>100	>100	>100	>100	>100	>100
							
32	G_3_-IPh-OEG-Lac_8_Cl (3c)	98.6 ± 15.1	82.4 ± 8.7	>100	>100	>100	>100
	PAMAM_3_-NH_2_	>100	>100	>100	>100	>100	>100
							
64^b^	PAMAM_4_-NH_2_	13.0 ± 0.8	6.0 ± 0.4	6.2 ± 0.7	50.2 ± 6.1	12.8 ± 1.5	4.7 ± 0.7

a Data presented as mean ± SD.

b Control measurement.

Our prior study^19^ highlighted a considerable disparity in the IC_50_ values among phosphonium CS-DDMs, extending over 3 orders of magnitude and dependent on the characteristics of the ligands attached to the phosphorus atoms. DDMs substituted with 4-methoxyphenyl ligands exhibited markedly lower cytotoxicity across a spectrum of cell lines (B14, BRL, and NRK). Our hypothesis suggested that the reduced cytotoxicity could be linked to the dispersal of positive charges across phosphorus atoms, which was facilitated by three electron-donating methoxy groups in para positions. This led us to hypothesize that introducing methoxy groups into the ortho positions, as seen in the CSn-TTMP series, might enhance this effect. In this study, we observed that both tested CS-P DDM series provided results consistent with prior findings,^19^ where cytotoxicity significantly increased with both the generation and concentration of the CS-P. However, it is important to note that the assessment of biocompatibility was conducted using different cell lines and methodologies (MTT and CV assay versus rezasurin), precluding a direct comparison of results. The CS-Ps were notably more toxic toward the THP1 cell line compared to MCF-7. For example, while the G_1_ DDM of the CS_n_-TTMP series was tolerated by MCF-7 cells, its IC_50_ for THP-1 was 7.8 μM after 24 h of exposure. For G_3_, there was approximately an order of magnitude difference in cytotoxicity (5.6 μM for MCF-7 and 0.3 μM for THP1).

While the CS-P and CS-N show constrained biocompatibility, a more accurate biocompatibility evaluation of these DDSs could be achieved by analyzing the cytotoxicity of the dendriplexes they form with genetic materials. This investigation will be pursued in a future study.

### 3.3 Hydrodynamic Size and Zeta Potential of the DDMs

Determining the hydrodynamic diameters (*d*_h_) and zeta potentials of dendritic nanoparticles and their aggregates is crucial for understanding their interactions with genetic materials. These characteristics are influenced by the pH and type of buffer, resulting in variable measurements across different media, such as water, phosphate buffer, PBS, and HEPES.^46^ This complicates direct comparisons across various compounds. For example, compounds measured in buffers containing relatively large ions, such as Tris or HEPES, exhibit larger hydrodynamic diameters compared to those measured in water or NaBr solution.^46^ In this study, the *d_h_* and zeta potentials were measured in phosphate buffer.

Interestingly, nanoparticles with identical NPU exhibited substantial variations in size, indicating the presence of both monomolecular nanoparticles and clusters (Table 2). Specifically, the CS-glyco series (compounds **1c**–**3c**) formed clusters ranging from 7.3 to 11.2 nm in diameter. This aggregation propensity aligns with prior findings regarding CS glycoDDMs,^28,29,41^ which also tended to cluster within a comparable size spectrum. Similarly, small aggregates of analogous dimensions were noted in the case of CS-N specifically for compounds **8** and **9**. In both CS-P DDM series, similar *d*_*h*_ values were obtained for the respective nanoparticle generations, ranging from 1.1 nm for G_1_ to 6.3 nm for G_3_. The obtained results correspond to the study of Wrobel et al.^47^ in which a comprehensive evaluation of biophysical properties of several series of CS-P was performed (samples in dH_2_O). The measured *d*_*h*_ of PAMAMs was similar to the reported values.^48^

**Table 2:** Zeta Potential and Hydrodynamic Size (*d_h_*) of the DDMs and/or Their Aggregates (clusters) and Parameters of siRNA Complexation by DDMs Calculated from Biophysical Assays–Zeta Potential and Gel Retardation Electrophoresis.^a^

				assay					
				zeta potential				gel electrophoresis	
NPU	compound	zeta [mV]	d_*h*_^f^ [nm]	MR_50_^c^^,^^g^	MR_sat_	CR_50_^e^	CR_sat_^e^	MR_sat_^h^	CR_sat_^e^
4	PAMAM_0_-NH_2_	-d	-d	-d	-d	-d	-d	-d	-d
	CS_0_-NH_2_	–3.5 ± 0.7	1.1 ± 0.2	73.0	-d	7.3	-d	225	23
	CS_1_–P-TTMP-Cl	13.3 ± 3.6	1.1 ± 0.1	8.9	12	0.9	1.2	50	5
	CS_1_–P-TDMP-Cl	18.0 ± 1.9	-d	10.3	12	1.0	1.2	50	5
									
8	G_1_-IPh-OEG-Lac_8_Cl	12.2 ± 1.9	8.5 ± 0.8^b^	6.0	12	1.2	1.2	175	35
	CS_1_–NH_2_	0.7 ± 0.1	4.7 ± 0.1^b^	13.9	-d	2.8	-d	50	10
	CS_2_–P-TTMP-Cl	13.5 ± 2.5	2.1 ± 1.0	3.2	8	0.6	1.6	20	4
	CS_2_–P-TDMP-Cl	6.1 ± 0.7	2.9 ± 0.8	4.8	8	1.0	1.6	20	4
	PAMAM_1_-NH_2_	-d	-d	-d	-d	-d	-d	2000	400
									
16	G_2_-IPh-OEG-Lac_8_Cl	9.7 ± 0.4	7.3 ± 0.5^b^	1.9	8	0.8	3.2	100	40
	CS_2_–NH_2_	1.1 ± 0.2	7.0 ± 0.5^b^	8.6	-d	3.4	-d	16	6
	CS_3_–P-TTMP-Cl	20.1 ± 1.1	3.0 ± 0.1	0.7	2	0.3	0.8	20	8
	CS_3_–P-TDMP-Cl	21.6 ± 1.0	3.5 ± 0.5	3.1	10	1.2	4.0	20	8
	PAMAM_2_-NH_2_	8.9 ± 1.8	3.4 ± 0.3	12.8	-d	5.1	-d	25	10
									
32	G_3_-IPh-OEG-Lac_8_Cl	10.6 ± 1.4	11.2 ± 1.2^b^	0.7	1	0.6	0.8	50	40
	PAMAM_3_-NH_2_	9.5 ± 1.0	3.7 ± 0.4	11.0	-d	8.8	-d	12	10
									
64	PAMAM_4_-NH_2_	7.4 ± 0.8	5.0 ± 0.1	0.1	-d	0.2	-d	5	8

a For both biophysical assays, the values represent a molar (charge) excess of the respective DDMs required to reach MR_50_/MR_sat_ (CR_50_/CR_sat_) of siRNA. The ξ and *d_h_* are given as the mean ± SD

b Experimentally measured size of dendrimer aggregates.^41^

c Points of zero crossing (Figure 2) were taken as MR_50_.

d Not detected/not detected within the measured range.

e Calc. for ∼40 negative phosphate charges on the siRNA backbone.^50^

f 10 μM solutions.

g The initial siRNA concentration was 0.2 μM.

h The initial siRNA concentration was 0.5 μM.

Electrophoretic light scattering is a technique that is based on the scattering of laser light by nanoscale objects as they move within an electric field. The value of the zeta potential, which is the potential at the boundary of the solvate shell, was calculated from the scattering data to provide insight into the surface charge of the nanoparticles. Given that most cellular membranes are negatively charged, the zeta potential of the nanoparticles is an important factor influencing their cellular uptake. The positive charge of the DDMs facilitates the penetration of the cell membranes, enabling the internalization of the dendriplexes. However, nanoparticles with a positive charge are often associated with increased toxicity due to their propensity to disrupt cellular membranes.

Except for CS_0_-NH_2_, the zeta potential of the majority of the tested DDMs was positive, yet near-neutral value (−3.5–13.5 mV, Table 2),^49^ which is in line with their chemical structure. A notable exception was both G_3_ CS-Ps (compounds **12b** and **15b**) exhibiting zeta potentials exceeding 20 mV. Zawadzki et al.^22^ reported a similar zeta potential value (20.3 mV, water) for his ammonium-terminated G_3_ CS DDM (G_3_Si PEG6000) having analogous structure, molecular weight, and NPU.

### 3.4 Dendriplex Formation Studies

The zeta potential and hydrodynamic size are essential parameters assessed for DDMs and/or their clusters, particularly concerning their interaction with siRNA. Two principal biophysical methods were used to evaluate siRNA complexation: electrophoretic light scattering and gel electrophoresis. These assays identified the molar excess of DDM required to reach the MR_50_/MR_sat_ (midpoint of complexation/saturation point) for siRNA complexation.^21^ The former refers to the point of charge offset, while the latter delineates the characteristics of dendriplexes when the complexation is completed, i.e., denoting the MR beyond which the measured parameter remains constant. The values derived from both methods reflect the critical ratio of DDM to siRNA required to reach these specified complexation milestones, offering insight into the biophysical dynamics of siRNA–DDM interactions.

#### 3.4.1 Dendriplex Zeta Potential Profiles

Shifts in the zeta potential upon the addition of the DDMs indicate the formation of dendriplexes within the examined compounds (Figure 2). The DDMs were incrementally introduced until reaching a 1:16 siRNA/DDM MR. Since the DDMs are cationic, either inherently or under experimental conditions, it is possible to calculate CR values. In this publication, the MR/CR values consistently represent the molar/charge excess of the DDM relative to siRNA. Comparing the CR values becomes more meaningful when DDMs of different sizes (generations) are considered, as this highlights the impact of the charge density per dendrimer molecule on the complexation process. Consequently, the discussion below focuses on the comparison based on CR values referred to as (±) excess. However, MR data are also presented, given the inconsistency in the literature regarding the use of MR or CR to characterize the nanoparticle complexation process.

**Figure 2:**
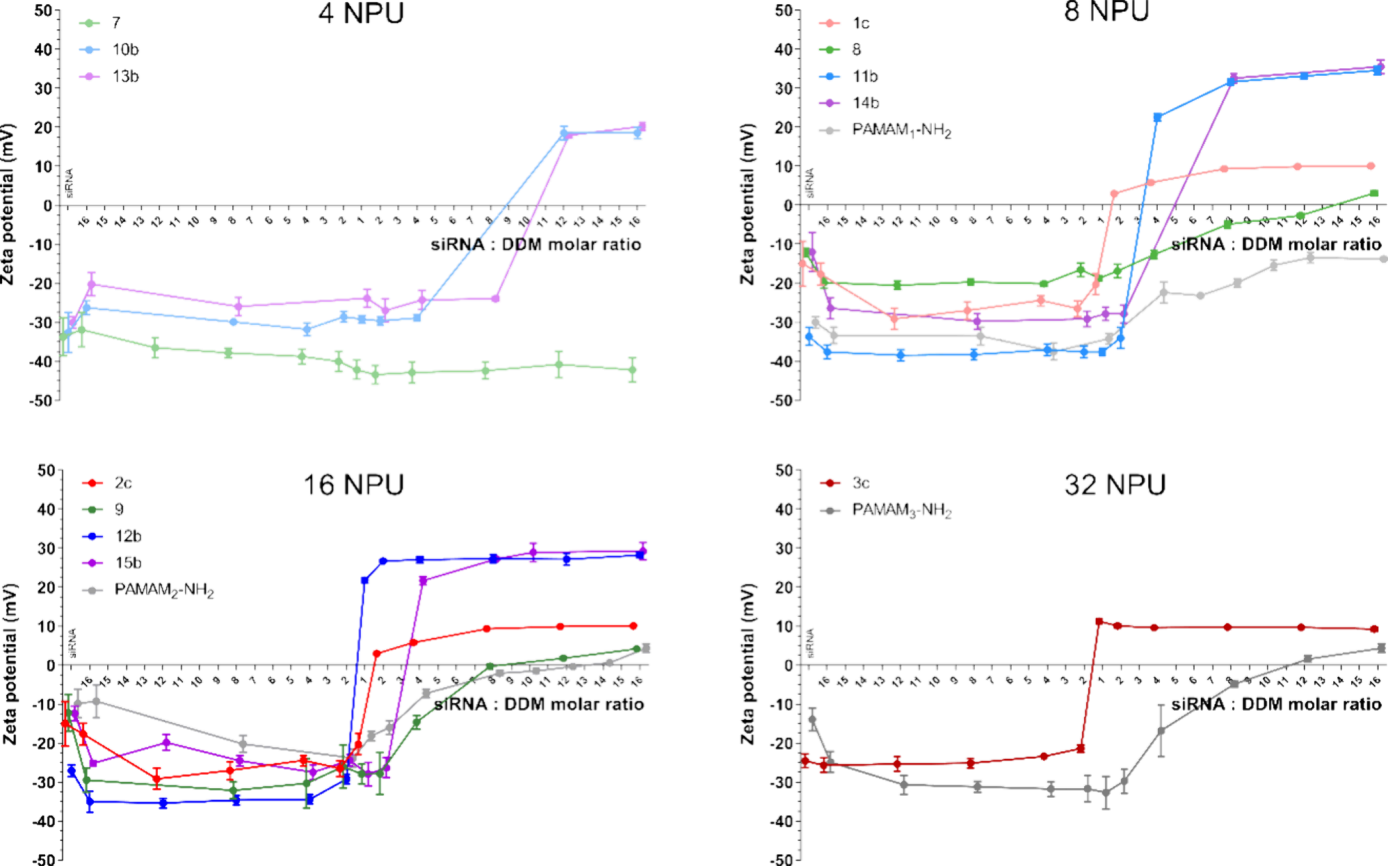
Zeta potential of the dendriplexes. The dendriplexes were prepared by mixing siRNA and DDMs over 16:1 to 1:16 siRNA/DDM molar CR in 10 mmol/L phosphate buffer at pH 7.4. The siRNA concentration was 0.2 μM, with the DDMs being incrementally added to the mixture. Results are given as mean ± SD from a minimum of four independent experiments.

The resulting complexation profiles exhibited a sigmoidal configuration, demonstrating a specific ratio at which the zeta potential of the dendriplexes transitions from negative to positive. The concentration of dendritic nanoparticles required for compensating for the negative charge of siRNA varied across different generations and structural types of dendritic nanoparticles (Table 2—zeta potential). For nanoparticles with 4 NPU, CS-P (compounds **10b** and **13b**, respectively) achieved the CR_50_ at a 1:1 DDM/siRNA CR. However, CS_0_-NH_2_ needed over a seven-fold (±) excess to achieve similar results, with PAMAM_0_-NH_2_ failing to attain charge neutralization under the experimental conditions. This trend was consistent with 8 NPU dendritic nanoparticles, underscoring the structure-dependent nature of complexation properties. Here, the CS-P (compounds **11b** and **14b**, respectively) and CS-glyco required roughly an equivalent (±) ratio to balance the negatively charged siRNA. In contrast, CS_1_-NH_2_ required about 3-fold (±) excess and PAMAM_1_-NH_2_ did not achieve CR_50_. With 16 NPU nanoparticles, CS_3_-P-TTMP-Cl neutralized siRNA at a 0.3:1 (±) ratio. An approximate 1:1 (±) ratio was sufficient for CS_3_-P-TDMP-Cl and G_2_-IPh-OEG-Lac_8_Cl to achieve charge neutralization, whereas CS_2_-NH_2_ and PAMAM_2_ needed considerably higher (±) excesses (3.4 and 5.1 times, respectively). For 32 NPU DDMs, a mere 0.6:1 (±) ratio of G_3_-IPh-OEG-Lac_16_Cl was sufficient to balance siRNA, while PAMAM_3_-NH_2_ required a nine-fold (±) excess to approach similar results. Saturation points (CR_sat_) were convincingly achieved solely with CS-glyco and CS-P within the tested ratio range. The zeta potential of dendriplexes formed with CS-P reached 20–30 mV, which was two to three times higher compared with those formed with CS-glyco (about 10 mV) upon reaching saturation. Analogous complexation profiles were obtained for PMe_3_ terminated DDMs with similar CR_50_ values.^20^ These findings highlight a stark contrast in the efficiency of CS-P and CS-glyco in complexing genetic material as opposed to CS-N and particularly PAMAMs, which, up to their second generation, demonstrated an inability to reach CR_50_ within the given molar scale.

#### 3.4.2 Gel Retardation Electrophoresis

Agarose gel electrophoresis, a commonly used method to study the formation of dendriplexes, utilizes the principle of the differential mobility of charged macromolecules (or their assemblies) within an electric field by navigating through an agarose gel matrix. The migration rate of the molecules and/or complexes is dictated by their charge density and molecular weight. This technique facilitates the identification of noncomplexed genetic material within the test samples.

**Figure 3:**
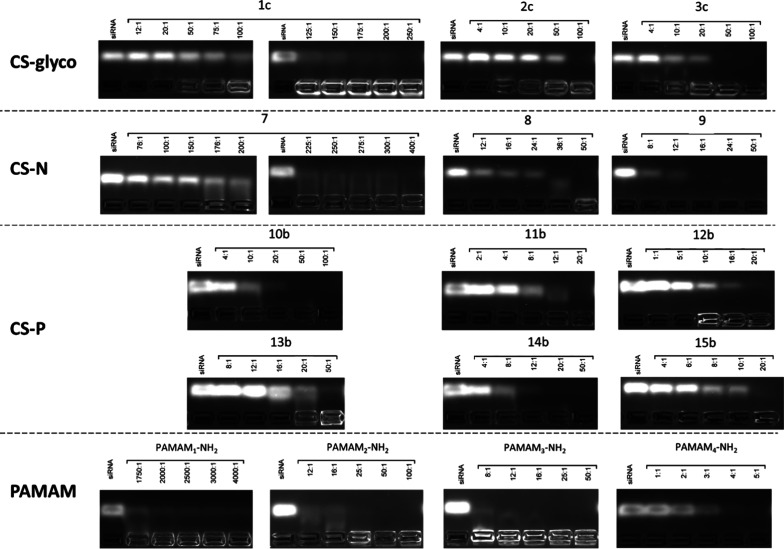
Gel retardation electrophoresis. Electropherograms were obtained for DDMs and siRNA complexed at specific MRs (DDM/siRNA). DDMs within a selected range of MRs were mixed with siRNA (0.5 μM) in 10 mM phosphate buffer (pH 7.4) to form dendriplexes. The samples were vortexed and incubated for 30 min before undergoing agarose gel electrophoresis (90 V, 45 min, TAE buffer). A control sample containing only siRNA was also run alongside the dendriplexes.

A series of comparative assays varying systematically in terms of the DDM’s type, generation, and CR was conducted to evaluate the capacity of the dendritic molecules to form complexes with negatively charged siRNA. The stained gels demonstrate that the noncomplexed siRNA (bright bands under UV irradiation) migrated toward the anode, whereas the neutral dendriplexes maintained their initial position (Figure 3). As the molar concentration of the DDMs increases, these bands progressively weaken and eventually disappear when all the siRNA is fully complexed. All newly developed dendritic vectors (CS-glyco, CS-N, and CS-P) successfully formed complexes with siRNA, irrespective of the peripheral modification, with distinctions evident among generations, respectively among DDMs having the same number of NPU.

Expectedly, different structural types of DDMs required varying amounts of excess for full siRNA complexation. However, the electrophoretic assay also highlighted disparities among dendritic nanovectors that share the same NPU. In the gel retardation assay, the MR_sat_ value was identified as the lowest MR tested at which the siRNA band completely disappeared. This indicated that the whole amount of siRNA was complexed by the DDMs; thus, the overall surface charge of the dendriplex was neutral or positive since it does not migrate toward anode. The CR_sat_ was calculated from this value (Table 2—gel electrophoresis). However, the dispersion in some bands suggests that binding by certain DDMs results in negatively charged intermediates that can to some extent migrate within the gel.

The differences in CR_sat_ can be demonstrated in compounds with 16 NPU: while G_2_-IPh-OEG-Lac8Cl reached CR_sat_ in a 40:1 (±) ratio, CS_2_-NH_2_, CS-P, and PAMAM_2_ required 4–6× lesser (±) ratio (6:1, 8:1, 10:1 DDM/siRNA, respectively) to achieve CR_sat_.

In certain scenarios, full saturation of siRNA was achieved using equivalent (±) excess across different generations of the same type of dendritic nanovectors. Specifically, CS-glyco **1c–3c** and G_2–4_**PAMAM-NH**_**2**_ reached CR_sat_ values ranging between 35 and 40 and between 8 and 10 (±) excess, respectively. This indicates that different structural types of DDMs have varying capacities to complex with siRNA, with charge density playing a key role.

In this study, the MRs/CRs needed to reach half-binding or full saturation during dendriplex formation, as identified through zeta potential measurements, were lower than those obtained by gel retardation assays. This variation in half-binding or saturation ratios, dependent on the biophysical method used, was described for other cationic DDMs.^21,26,51,52^ Krasheninina et al.^21^ investigated dendriplex formation in ammonium-terminated CS-DDMs and siRNA by four biophysical methods: circular dichroism (CD), ethidium bromide intercalation assay (EB), gel retardation electrophoresis, and zeta potential measurements. In their experiments, similar CR_sat_ values were obtained by gel electrophoresis, CD, and EB, while CR_sat_ values from the zeta potential were three to four times higher. It is necessary to consider that the complexation of NAs by DDMs is governed by electrostatic interactions that are influenced by bulk conditions such as pH and ionic strength, along with the inherent physicochemical properties of the DDMs/NAs involved.^53^ Despite focusing on the same characteristic, gel electrophoresis and zeta potential measurements are biophysical methods for characterizing polyplexes that are conducted under their specific conditions (refer to section 2), which may yield differing results.

Additionally, a study exploring the rapid exchange between free and bound states in RNA–DDM polyplexes offers a further perspective on the subject.^54^ Dendriplexes are liable to spontaneous disintegration into smaller complexes, wherein RNA swiftly transitions in milliseconds between its free and dendriplex-bound forms. Based on this, processes occurring during siRNA binding to the cationic DDMs are described.^21,53^ Initially, the mutual interaction leads to distortion of the NA duplex structure (corresponds to the MR_50_/CR_50_ values, Table 2). Subsequently, upon increasing the DDM concentration, the dendriplex rearrange due to the reversibility of their architecture. The presence of more DDM molecules compared to earlier stages increases the surface charge of the dendriplexes. This dendriplex state can be referred to as MR_sat_/CR_sat_. Given the dynamic formation of dendriplexes, we suggest that two distinct packing states, each defined by specific MR_50_/CR_50_ and MR_sat_/CR_sat_ values, emerged during the experiments, as reflected in the different outcomes between gel electrophoresis and zeta potential measurements.

An important consideration for further biological testing is determining the most appropriate (±) ratio in dendriplexes. Low (±) ratios result in the formation of soluble anionic aggregates. Such aggregates can to some extent migrate through the agarose gel toward anode as observed in a certain dendriplex we tested (Figure 3). An excess of DDMs (CR_sat_, Table 2) leads to overcharging of the anionic NA, forming cationic complexes. These cationic dendriplexes are considered the most effective for NA delivery.^53^

The study by Kresheninina et al.^21^ favors results obtained from zeta potential profiles for *in vitro* experiments. While minimizing DDM content is beneficial due to the toxicity associated with high positive surface charges, a higher charge enhances interactions with negatively charged cell membranes^55,56^ and promotes smaller nanoparticle sizes.^26,51,52,57^ These factors are crucial for effective cellular uptake.^53,55,58^ Additionally, in some instances, an excess of positive charge led to an increased transfection efficiency. For example, Peng et al.^56^ investigated G_4_-PAMAM/DNA dendriplexes and identified three distinct packaging states based on the (±) CR, with dendriplexes possessing a higher positive surface charge showing more effective transfection in HT1080 cells.

Thus, testing dendriplexes at various (±) ratios would be ideal to pinpoint the optimal composition for transfection experiments. It is crucial to balance the beneficial role of free DDM in enhancing the transfection efficiency against its negative impacts on the colloidal stability and cytotoxicity. In this context, the highly biocompatible CS-glyco is particularly advantageous, as its increased content in dendriplexes is less likely to lead to significant toxic effects. Additionally, excess free DDM in the formulation influences interactions with the surrounding bulk medium such as the formation of a protein corona. This interaction, in turn, can alter the pharmacokinetics and pharmacodynamics of the dendriplexes.^59^

### 3.5 Nuclease Protection Assay

**Figure 4:**
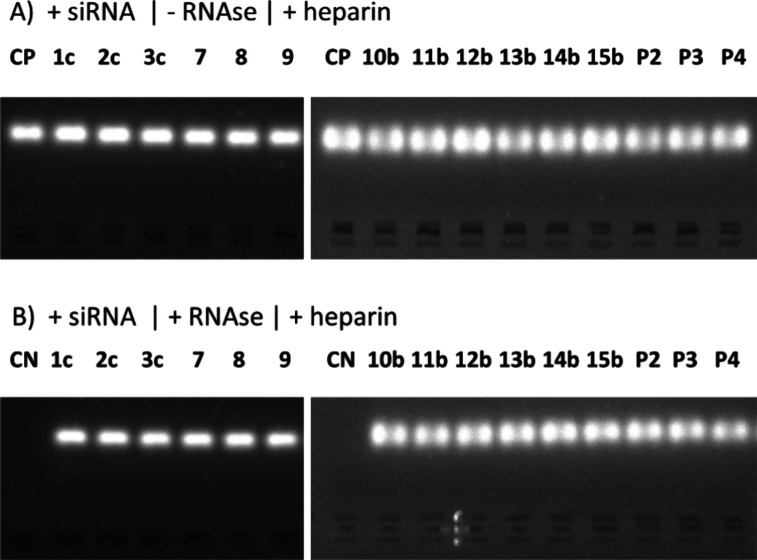
Nuclease protection assay. Dendriplexes were prepared by mixing siRNA (0.5 μM) and DDMs at their respective MR_sat_ ratio (see Table 2, gel electrophoresis). Samples were subsequently treated (A) with heparin (0.082 mg/mL) or (B) with RNase solution (0.25 μg/mL, 30 min incubation at 37 °C), followed by heparin. PAMAM-NH_2_ DDMs were labeled as P2 (PAMAM_2_-NH_2_), P3 (PAMAM_3_-NH_2_), and P4 (PAMAM_4_-NH_2_). Negative/positive control samples were run along with the dendriplexes: pure siRNA (CN) and siRNA treated with RNase (CP). Samples were then subjected to agarose gel electrophoresis (90 V, 45 min in TAE buffer). The gels with samples were visualized under UV light (365 nm). P2, PAMAM_2_-NH_2_; P3, PAMAM_3_-NH_2_; P4, PAMAM_4_-NH_2_.

In the context of gene delivery vectors, a pivotal characteristic is their capacity for safeguarding the genetic payload against enzymatic degradation by nucleases. A nuclease protection assay was conducted to evaluate this protective attribute.^58,60^ The assay utilized dendriplexes prepared from the DDMs conjugated with siRNA at a gel electrophoresis-based MR_sat_, i.e., the MR in which they effectively bound the whole amount of the genetic material (see Table 2, MR_sat_ for gel electrophoresis). Polyanionic molecules, such as heparin, have the potential to disrupt the dendriplex structure and release siRNA. This liberation was demonstrated in all tested samples through gel electrophoresis assays in which bands of the free siRNA in the heparin-treated samples were visualized under UV light (Figure 4A).

The formation of dendriplexes can protect NAs from degradation by the restriction nucleases.^46,48,53,55^ To examine this, the dendriplexes were incubated with the RNase solution followed by the addition of heparin. The detection of siRNA bands after the RNase treatment of dendriplexes confirms the protective properties of the dendriplexes against RNase degradation (Figure 4A). Additionally, a control experiment was performed using free siRNA exposed to RNase (referred to as CP), which verified the degradation of siRNA in the absence of protection by the dendriplex. Protective properties were previously confirmed for other dendritic compounds, such as polyamine,^61^ phosphorus,^26^ and CS-DDMs.^20,62^ The findings from the nuclease protection assay indicate that the tested dendriplexes show promising protective characteristics to deliver therapeutic genetic material.

## 4 Conclusions

This study introduces synthetic methodologies and characterizes three novel types of cationic dendritic nanocarriers based on the CS scaffold, each differentiated by peripheral modification: CS-glyco, CS-N, and CS-P. Notably, CS-glyco is the first glycoDDMs with an inherently cationic nature, featuring a quaternized triazole ring designed specifically for complexing polyanionic compounds like therapeutic siRNA. The dendritic nanocarriers varied in cytotoxicity, biophysical properties, and complexation capabilities, a finding of particular importance when compared to widely used PAMAMs under identical experimental conditions.

CS-glyco and PAMAMs exhibited significantly lower toxicity to MCF-7 and THP-1 cell lines than CS-N and CS-P, having the same NPUs. Despite their charged nature, CS-glyco possessing up to 32 charged functional moieties maintained biocompatibility comparable to that of previously described neutral CS glycoDDMs, highlighting the exceptional capability of sugar coating to reduce the toxicity of the dendritic scaffolds. This study underscores that while cationic charge density is generally viewed as the primary determinant of toxicity, careful modification of the dendritic scaffold’s structural composition can yield biocompatible, inherently cationic dendritic nanovectors.

Except for CS_0_-NH_2_, the majority of the tested DDMs exhibited a positive yet near-neutral zeta potential in the phosphate buffer solution, enabling interaction with polyanionic compounds like siRNA. As indicated by previous findings, the nanoparticle size measurement revealed the tendency of dendritic nanoparticles to form macromolecular associates. Monomolecular nanoparticles were detected only in CS-P and PAMAMs. The formation of dendriplexes was confirmed using two complementary methods: zeta potential profiles and gel retardation electrophoresis, both verifying the complexation of model siRNA by the DDMs. Differences in the siRNA/DDM ratios required to reach saturation, observed among various structural types and even among DDMs with identical numbers of NPU, suggest that factors such as structure, composition, and aggregation behavior also significantly influence complexation dynamics. The dendriplexes formed were effective in protecting the model siRNA from degradation by RNase and facilitated the release of the genetic material when exposed to heparin.

The study establishes a foundational background for future advancements in drug delivery research by introducing promising dendritic nanocarriers and detailing differences in their performance through biophysical and biological evaluations.

## Supporting Information Available

List of compounds, data for compounds **1c**–**15b**, NMR spectra for compounds **1c**–**15b** (PDF)
