# Supplementary material for: Comparative Study of Functionalized Carbosilane Dendrimers for siRNA Delivery: Synthesis, Cytotoxicity, and Biophysical Properties
Source: ACS Omega. 2024 Dec 20;10(1):1047–60. doi: 10.1021/acsomega.4c08314 (PMC11740622; doi:10.1021/acsomega.4c08314)
Supplement: Supplementary file 1 — ao4c08314_si_001.pdf [file ao4c08314_si_001.pdf]

SUPPORTING INFORMATION

Comparative study of functionalized carbosilane dendrimers for siRNA delivery: synthesis, cytotoxicity, and biophysical properties

*Monika Müllerová[a,c], Piotr Tarach[b], Tomáš Strašák[a], Petra Cuřínová[a,d], Roman Petrickovic[a], Táňa Závodná[c], Jan Topinka[c], Anna Janaszewska[b], Barbara Klajnert-Maculewicz[b], Lucie Červenková Šťastná[a]**

[a] Institute of Chemical Process Fundamentals Czech Academy of Sciences, Rozvojová 135, 165 02 Prague, Czech Republic

[b] University of Lodz, Faculty of Biology and Environmental Protection, Department of General Biophysics, Pomorska 141/143, 90-236 Lodz, Poland

[c] Institute of Experimental Medicine, Czech Academy of Sciences, Vídeňská 1083, 142 00 Prague, Czech Republic

[d] University of Chemistry and Technology Prague, Department of Organic Chemistry, Technická 5, 166 28 Prague 6, Czech Republic

Table of Contents:

List of compounds S3
1. Data for compounds **1c – 15b** S7
2. NMR spectra of the compounds **1c – 15b** S18

CS-glyco

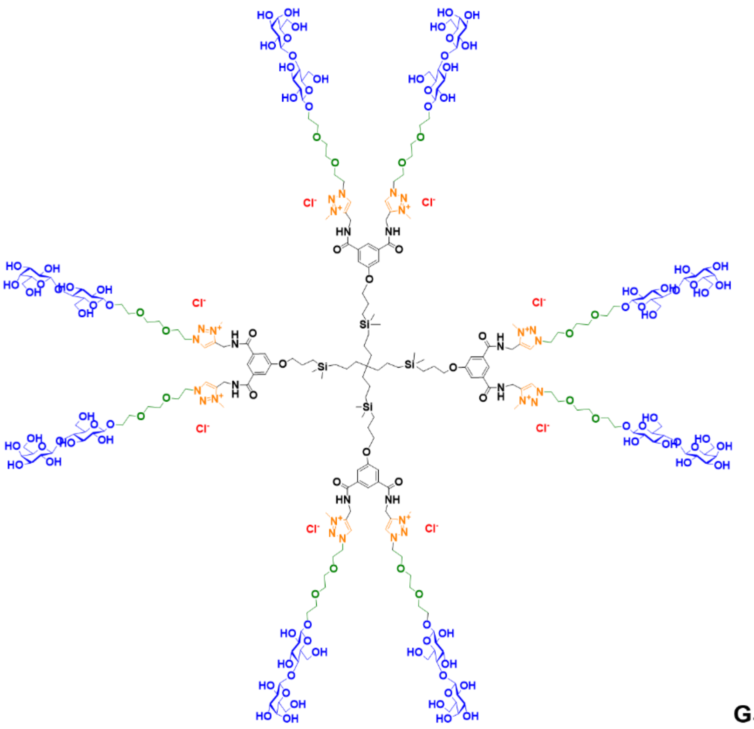

1c
G_1-IPh-OEG-Lac_8Cl

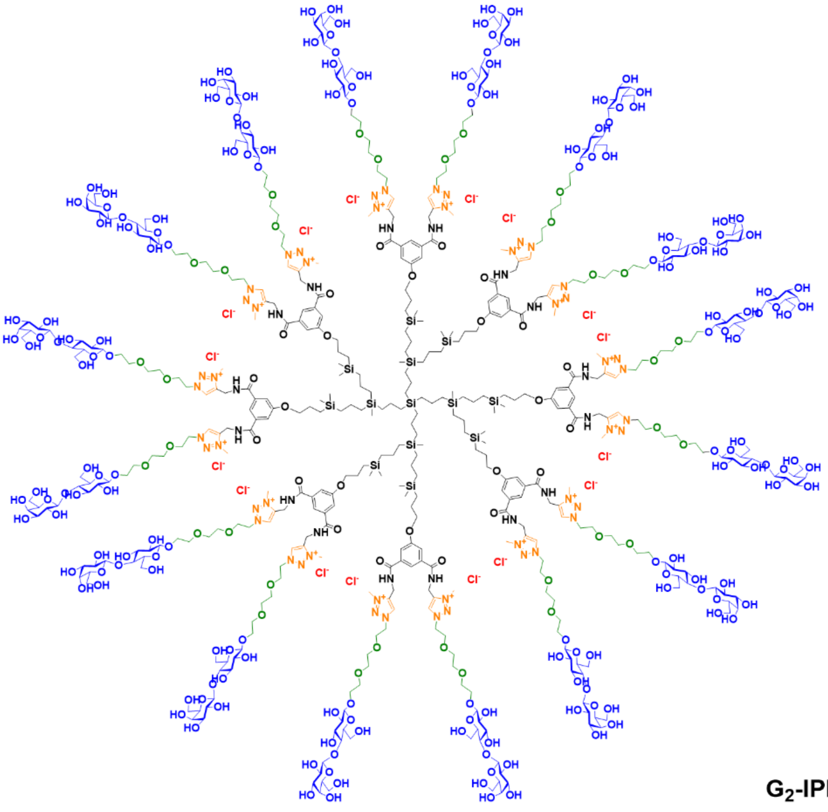

2c
G_2-IPh-OEG-Lac_{16}Cl

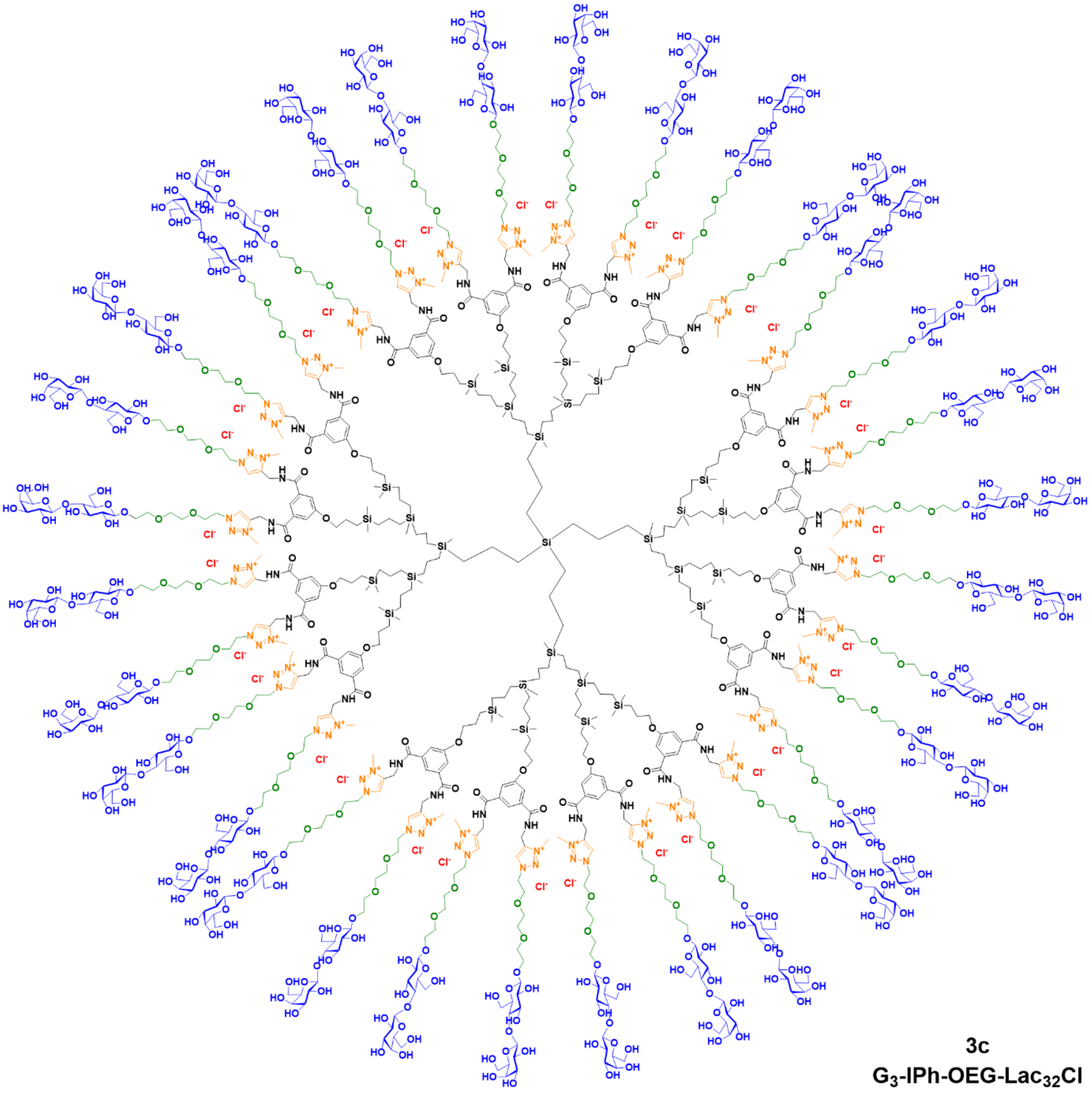

3c
G_3-IPh-OEG-Lac_{32}Cl

CS-N

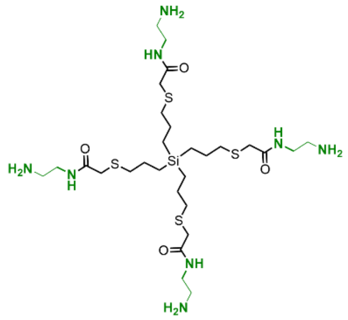

7
CS_0-NH_2

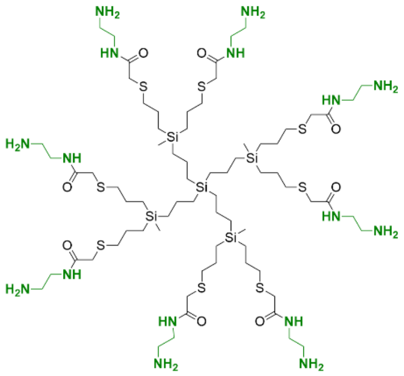

8
CS_1-NH_2

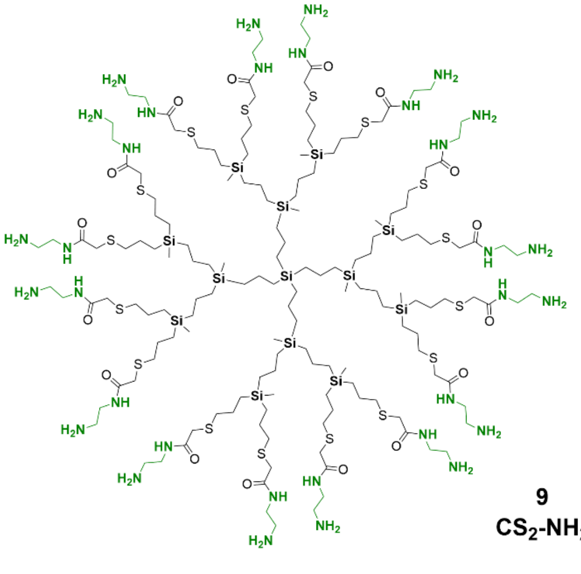

9
CS_2-NH_2

CS-P

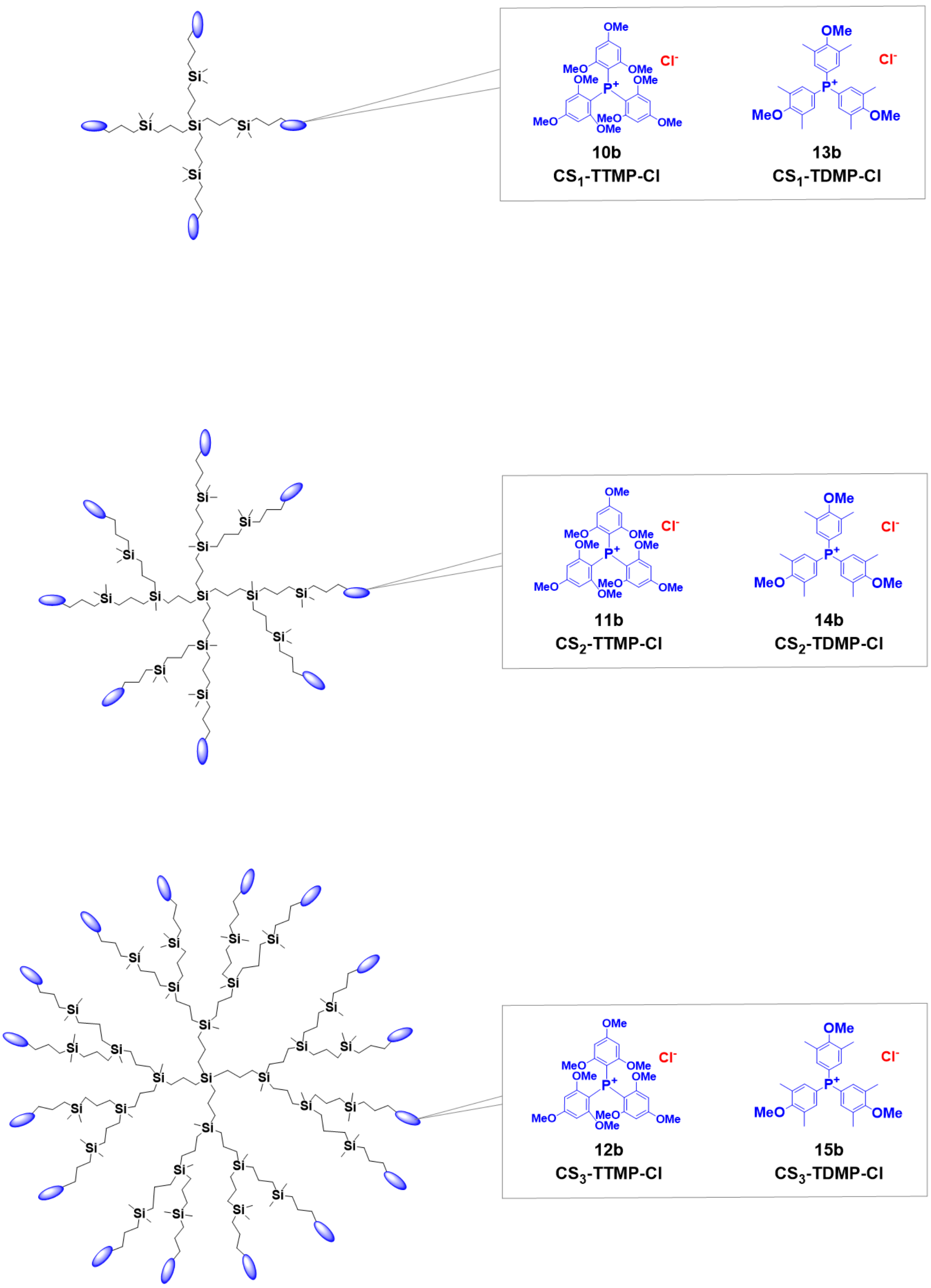

Scheme S1: Chemical structures of compounds **1c – 15b**.

1. Data for compounds 1c – 15b

CS-glyco

Data for **1c**: ^{1}H NMR (400 MHz, DMSO-d_6, H-H COSY): δ 9.81 (br s, 8H, N*H*), 8.94 (br s, 8H, C*H*$_{\text{Tria}}$), 8.25 (br s, 4H, C*H*$_{\text{Ph}}$), 7.63 (br s, 8H, C*H*$_{\text{Ph}}$), 5.11 (d, *J* = 4.8 Hz, 16H, O*H*-2, O*H*-2′), 4.84 (br s, 8H, O*H*-3′), 4.81–4.72 (m, 48H, O*H*-3, O*H*-6, NC*H*$_2$, NHC*H*$_2$), 4.57 (m, 16H, O*H*-4′, O*H*-6′), 4.39 (s, 24H, N*Me*), 4.22–4.20 (m, 16H, H-1, H-1′), 4.00 (br s, 8H, C*H*$_2$OC$_q$), 3.94–3.92 (m, 16H, OC*H*$_2$), 3.83–3.80 (m, 8H, OCH*H*), 3.73 (dd, *J* = 11.1, 5.2 Hz, H-8, H-6′$_a$), 3.62 (br s, 8H, H-4′), 3.55–3.45 (m, 88H, H-5, H-6a/b, H-6′b, 3C*H*$_2$O, OCH*H*), 3.31–3.27 (m, 40H, H-3, H-4, H-2′, H-3′, H-5′), 3.00 (ddd, *J* = 8.3, 7.9, 4.9 Hz, 8H, H-2), 1.69 (br s, 8H, OCH$_2$C*H*$_2$CH$_2$), 1.34 (br s, 8H, Si^{0}CH$_2$C*H*$_2$), 0.58–0.56 (br s, 24H, SiC*H*$_2$), –0.04 (s, 24H, Si*Me*$_2$). ^{13}C {^{1}H} NMR (101 MHz, DMSO-d_6, HSQC, HMBC, HSQC TOSCY): δ 165.9 (*C*O), 158.8 (*C*$_q$O), 141.2 (*C*$_{\text{q(Tria)}}$), 134.6 (*C*$_{\text{q(Ph)}}$), 130.0 (*C*H$_{\text{Tria}}$), 118.9 (*C*H$_{\text{Ph}}$), 116.6 (2*C*H$_{\text{Ph}}$), 103.9 (C-1′), 102.5 (C-1), 80.8 (C-4), 75.5 (C-5), 75.0 (C-3), 74.8 (C-5′), 73.3 (C-2′), 73.1 (C-2), 70.8 (*C*H$_2$OC$_q$), 70.6 (C-3′), 69.64, 69.57, 69.5 (3×O*C*H$_2$), 68.1/68.0 (C-4′, O*C*H$_2$), 67.4 (O*C*H$_2$), 60.44, 60.35 (C-6/6′), 53.1 (CH$_2$*C*H$_2$N), 38.1 (N*Me*), 32.3 (*C*H$_2$NH), 23.3 (CH$_2$*C*H$_2$CH$_2$O), 19.5 (Si^{0}CH$_2$CH$_2$*C*H$_2$), 18.2 (Si^{0}CH$_2$*C*H$_2$), 17.0 (Si^0*C*H$_2$), 11.0 (*C*H$_2$CH$_2$CH$_2$O), –3.4 (Si*Me*$_2$). ^{29}Si {^{1}H} NMR (79 MHz, DMSO-d_6): δ 2.22 (Si^1), not detected (Si^0).

Data for **2c:** ^{1}H NMR (400 MHz, DMSO-d_6, H-H COSY): δ 9.85 (br s, 16H, N*H*), 8.98 (br s, 16H, C*H*$_{\text{Tria}}$), 8.27 (br s, 8H, C*H*$_{\text{Ph}}$), 7.62 (br s, 16H, C*H*$_{\text{Ph}}$), 5.13 (d, *J* = 4.4 Hz, 32H, O*H*-2, O*H*-2′), 4.89 (br s, 16H, O*H*-3′), 4.80 (m, 96H, O*H*-3, O*H*-6, NC*H*$_2$, NHC*H*$_2$), 4.62 (m, 32H, O*H*-4′, O*H*-6′), 4.69 (s, 48H, N*Me*), 4.22–4.20 (m, 32H, H-1, H-1′), 3.99 (br s, 16H, C*H*$_2$OC$_q$), 3.92 (br s, 32H, OC*H*$_2$), 3.82–3.79 (m, 16H, OCH*H*), 3.72 (dd, *J* = 11.1, 4.4 Hz, 16H, H-6′a), 3.62 (br s, 16H, H-4′), 3.54–3.44 (m,176H, H-5, H-6a/b, H-6′b, 3C*H*$_2$O, OCH*H*), 3.31–3.26 (m, 80H, H-3, H-4, H-2′, H-3′, H-5′), 2.99 (ddd, *J* = 8.4, 7.9, 4.4 Hz, 16H, H-2), 1.67 (br s, 16H, OCH$_2$C*H*$_2$CH$_2$), 1.32 (br s, 24H, Si^{0,1}CH$_2$C*H*$_2$), 0.54 (br s, 64H, SiC*H*$_2$), –0.06 (s, 48H, Si^2*Me*$_2$), –0.11 (s, 12H, Si^1*Me*). ^{13}C {^{1}H} NMR (101 MHz, dmso-d_6, HSQC, HMBC, HSQC TOCSY): δ 166.0 (*C*O), 158.8 (*C*$_q$O), 141.2 (*C*$_{\text{q(Tria)}}$), 134.6 (*C*$_{\text{q(Ph)}}$), 130.1 (*C*H$_{\text{Tria}}$), 119.1 (*C*H$_{\text{Ph}}$), 116.7 (2*C*H$_{\text{Ph}}$), 103.8 (C-1′), 102.5 (C-1), 80.7 (C-4), 75.5 (C-5), 75.0 (C-3), 74.9 (C-5′), 73.3 (C-2′), 73.1 (C-2), 70.8 (*C*H$_2$OC$_q$), 70.7 (C-3′), 69.65, 69.58, 69.5 (3×O*C*H$_2$), 68.0 (C-4′, O*C*H$_2$), 67.4 (O*C*H$_2$), 60.4, 60.3 (C-6/6′), 53.1 (CH$_2$*C*H$_2$N), 38.2 (N*Me*), 32.3 (*C*H$_2$NH), 23.4 (CH$_2$*C*H$_2$CH$_2$O), 19.4 (Si^{1}CH$_2$CH$_2$*C*H$_2$Si^2), 18.6 (Si^{0}CH$_2$CH$_2$*C*H$_2$), 18.3 (*C*H$_2$CH$_2$Si^{1}CH$_2$*C*H$_2$), 18.1 (Si^1*C*H$_2$CH$_2$CH$_2$Si^2), 17.1 (Si^0*C*H$_2$), 10.9 (*C*H$_2$CH$_2$CH$_2$O), –3.4

(Si^{2}*Me*$_{2}$), –4.8 (Si^{1}*Me*).^{29}Si {^{1}H} NMR (79 MHz, DMSO-*d*$_{6}$): *δ* 2.17 (Si^{2}), 1.10 (Si^{1}), not detected (Si^{0}).

Data for **3c:** ^{1}H NMR (400 MHz, DMSO-*d*$_{6}$, H-H COSY): *δ* 9.81 (br s, 32H, N*H*), 8.97 (br s, 32H, C*H*$_{\text{Tria}}$), 8.27 (br s, 16H, C*H*$_{\text{Ph}}$), 7.60 (br s, 32H, C*H*$_{\text{Ph}}$), 5.12 (br s, 64H, O*H*-2, O*H*-2′), 4.87 (br s, 32H, O*H*-3′), 4.79 (br s, 64H, NC*H*$_{2}$), 4.73 (br s, 96H, O*H*-6, NHC*H*$_{2}$), 4.60 (br s, 96H, O*H*-3, O*H*-4′, O*H*-6′), 4.38 (s, 96H, N*Me*), 4.22–4.20 (m, 64H, H-1, H-1′), 3.92 (br s, 96H, C*H*$_{2}$OC$_{q}$, OC*H*$_{2}$), 3.83–3.79 (m, 32H, OCH*H*), 3.74–3.69 (m, 32H, H-6′a), 3.62 (br s, 32H, H-4′), 3.54–3.44 (m,352H, H-5, H-6a, H-6b, H-6′b, 3C*H*$_{2}$O, OCH*H*), 3.31–3.26 (m, 160H, H-3, H-4, H-2′, H-3′, H-5′), 3.02–2.97 (m, 32H, H-2), 1.67 (br s, 32H, OCH$_{2}$C*H*$_{2}$CH$_{2}$), 1.32 (br s, 56H, Si^{0,1,2}CH$_{2}$C*H*$_{2}$), 0.55 (br s, 144H, SiC*H*$_{2}$), –0.06 (s, 96H, Si^{3}*Me*$_{2}$), –0.10 (s, 36H, Si^{1,2}*Me*). ^{13}C {^{1}H} NMR (101 MHz, dmso-*d*$_{6}$, HSQC, HMBC, HSQC TOCSY): *δ* 166.0 (*C*O), 158.8 (*C*$_{q}$O), 141.2 (*C*$_{\text{q(Tria)}}$), 134.6 (*C*$_{\text{q(Ph)}}$), 130.1 (*C*H$_{\text{Tria}}$), from HMBC 119.0 (*C*H$_{\text{Ph}}$), 116.7 (2*C*H$_{\text{Ph}}$), 103.8 (C-1′), 102.5 (C-1), 80.7 (C-4), 75.5 (C-5), 75.0 (C-3), 74.9 (C-5′), 73.3 (C-2′), 73.1 (C-2), 70.8 (*C*H$_{2}$OC$_{q}$), 70.7 (C-3′), 69.65, 69.59, 69.5 (3×O*C*H$_{2}$), 68.1, 68.0 (C-4′, O*C*H$_{2}$), 67.4 (O*C*H$_{2}$), 60.4, 60.3 (C-6/6′), 53.1 (CH$_{2}$*C*H$_{2}$N), 38.2 (N*Me*), 32.3 (*C*H$_{2}$NH), 23.3 (CH$_{2}$*C*H$_{2}$CH$_{2}$O), 19.38, 19.36 (Si^{2}CH$_{2}$*C*H$_{2}$*C*H$_{2}$Si^{3}), not detected or overlapped (Si^{0,1}*C*H$_{2}$*C*H$_{2}$*C*H$_{2}$Si^{1,2}), 18.1 (Si^{2}*C*H$_{2}$CH$_{2}$CH$_{2}$Si^{3}), 10.9 (*C*H$_{2}$CH$_{2}$CH$_{2}$O), –3.4 (Si^{3}*Me*$_{2}$), –4.8 (Si^{1,2}*Me*).^{29}Si {^{1}H} NMR (79 MHz, DMSO-*d*$_{6}$): *δ* 2.16 (Si^{3}), 1.07 (Si^{1,2}), not detected (Si^{0}).

CS-N

Data for **4**: ^{1}H NMR (400 MHz, DMSO-*d*$_{6}$, H-H COSY): *δ* 3.63 (s, 12H, *Me*), 3.31 (s, 8H, SC*H*$_{2}$COOMe), 2.58 (t, *J* = 7.2 Hz, 8H, CH$_{2}$C*H*$_{2}$S), 1.56–1.46 (m, 8H, SiCH$_{2}$C*H*$_{2}$), 0.61–0.56 (m, 8H, SiC*H*$_{2}$). ^{13}C {^{1}H} NMR (101 MHz, DMSO-*d*$_{6}$, HSQC, HMBC): *δ* 170.7 (*C*O), 52.0 (*Me*), 35.6 (SiCH$_{2}$CH$_{2}$*C*H$_{2}$), 32.6 (*C*H$_{2}$COOMe), 23.3 (SiCH$_{2}$*C*H$_{2}$), 11.2 (Si*C*H$_{2}$). ^{29}Si {^{1}H} NMR (79 MHz, DMSO-*d*$_{6}$): *δ* 3.66.

HRMS: (ESI): *m/z* $[M + H]^{+}$ calc. for $C_{24}H_{44}O_{8}S_{4}Si$: 617.1761, found: 617.1759

Data for **5**: ^{1}H NMR (400 MHz, DMSO-*d*$_{6}$, H-H COSY): *δ* 3.62 (s, 24H, COO*Me*), 3.29 (s, 16H, SC*H*$_{2}$COOMe), 2.56 (t, *J* = 7.2 Hz, 16H, CH$_{2}$C*H*$_{2}$S), 1.54–1.46 (m, 16H, SCH$_{2}$C*H*$_{2}$), 1.34–1.26 (m, 8H, Si^{0}CH$_{2}$C*H*$_{2}$), 0.57–0.53 (m, 32H, Si^{0}C*H*$_{2}$CH$_{2}$C*H*$_{2}$Si^{1}C*H*$_{2}$), –0.07 (s, 12H, Si*Me*). ^{13}C {^{1}H} NMR (101 MHz, DMSO-*d*$_{6}$, HSQC, HMBC): *δ* 170.7 (*C*O), 52.0 (COO*Me*),

35.6 (CH_2CH_2S), 32.7 (*C*H_2COOMe), 23.4 (*C*H_2CH_2S), 18.2 ($SiCH_2$*C*H_2), 18.1 ($Si^0CH_2CH_2$*C*H_2), 17.1 (Si^0*C*H_2), 12.8 (*C*$H_2CH_2CH_2S$), –5.2 (Si*Me*). ^{29}Si {^{1}H} NMR (79 MHz, DMSO-d_6): δ 2.61 (Si^1), 0.87 (Si^0).

HRMS: (ESI): *m/z* [M + H]$^+$ calc. for $C_{64}H_{124}O_{16}S_8Si_5$: 1547.5573, found: 1547.5578.

Data for **6**: ^{1}H NMR (400 MHz, DMSO-d_6, H-H COSY): δ 3.62 (s, 48H, COO*Me*), 3.27 (s, 32H, SC*H*$_2$CO), 2.56 (t, J = 7.2 Hz, 32H, CH_2C*H*$_2$S), 1.54–1.46 (m, 32H, SCH_2C*H*$_2$), 1.34–1.26 (m, 24H, $Si^{0,1}CH_2$C*H*$_2$), 0.57–0.51 (m, 80H, SiC*H*$_2$), –0.07 (s, 24H, Si^2*Me*), –0.10 (s, 12H, Si^1*Me*). ^{13}C {^{1}H} NMR (101 MHz, DMSO-d_6, HSQC, HMBC): δ 170.6 (*C*O), 51.9 (COO*Me*), 35.6 (CH_2*C*H_2S), 32.6 (*C*H_2COOMe), 23.4 (*C*H_2CH_2S), 18.5 ($Si^0CH_2CH_2$*C*H_2), 18.3 (*C*$H_2CH_2Si^1$*C*H_2), 18.0 (Si^1CH_2*C*H_2*C*H_2Si^2), 17.1 (Si^0*C*H_2), 12.8 (*C*$H_2CH_2CH_2S$), –4.9 (Si^1*Me*), –5.2 (Si^2*Me*). ^{29}Si {^{1}H} NMR (79 MHz, DMSO-d_6): δ 2.60 (Si^2), 1.11 (Si^1), not detected (Si^0).

HRMS: (ESI+): *m/z* [M + NH_4]$^+$ calc. for [$C_{144}H_{284}O_{32}S_{16}Si_{13}NH_4$]$^+$: 3428.3037, found: 3428.3033.

Data for **7**: ^{1}H NMR (400 MHz, DMSO-d_6, H-H COSY): δ 8.00 (t, J = 5.7 Hz, 4H, N*H*), 3.08 (s, 8H, C*H*$_2$CO), 3.04 (td, J = 6.2, 5.7 Hz, 8H, NHC*H*$_2$), 2.57 (t, J = 6.2 Hz, 8H, C*H*$_2$NH$_2$), 2.55 (t, J = 7.5 Hz, 8H, $SiCH_2CH_2$C*H*$_2$), 1.53–1.45 (m, 8H, $SiCH_2$C*H*$_2$), 0.59–0.55 (m, 8H, SiC*H*$_2$). ^{13}C {^{1}H} NMR (101 MHz, DMSO-d_6, HSQC, HMBC): δ 169.2 (*C*O), 42.3 (*C*H_2NH_2), 41.2 (*C*H_2NH), 35.6 ($SiCH_2CH_2$*C*H_2), 34.3 (CO*C*H_2), 23.5 ($SiCH_2$*C*H_2), 11.3 (Si*C*H_2). ^{29}Si {^{1}H} NMR (79 MHz, DMSO-d_6): δ 3.56.

HRMS: (ESI): *m/z* [M + H]$^+$ calc. for $C_{28}H_{60}N_8O_4S_4Si$: 729.3462, found 729.3462.

Data for **8**: ^{1}H NMR (400 MHz, DMSO-d_6, H-H COSY): δ 8.02 (t, J = 5.5 Hz, 8H, N*H*), 3.07 (br s, 32H, C*H*$_2$CONHC*H*$_2$), 2.72 (br s, 16H, C*H*$_2$NH$_2$), 2.57 (t, J = 6.8 Hz, 16H, CH_2C*H*$_2$S), 1.51–1.47 (m, 16H, SCH_2C*H*$_2$), 1.32–1.27 (m, 8H, $SiCH_2$C*H*$_2$), 0.56–0.51 (m, 32H, SiC*H*$_2$),

0.08(s, 12H, Si*Me*). ^{13}C {^{1}H} NMR (101 MHz, DMSO-d_6, HSQC): δ 169.2 (*C*O), 42.1 (*C*H_2NH), 41.1 (*C*H_2NH_2), 35.6 (S*C*$H_2CH_2CH_2$), 34.6 (CO*C*H_2), 23.6 (SCH_2*C*H_2), 18.21, 18.17 (Si$^0CH_2$*C*H_2*C*H_2), 17.1 (Si^0*C*H_2), 13.0 (SCH_2CH_2*C*H_2), –5.1 (Si*Me*). ^{29}Si {^{1}H} NMR (79 MHz, DMSO-d_6): δ 2.55 (Si^1), 0.84 (Si^0).

HRMS: (ESI+): *m/z* $[M + H]^+$ calc. for $[C_{72}H_{157}N_{16}O_8S_8Si_5]^+$: 1771.8983, found 1771.8975, $[M + 2H]^{2+}$ calc. for $[C_{72}H_{158}N_{16}O_8S_8Si_5]^{2+}$: 886.4528, found 886.4520.

Data for **9**: ^{1}H NMR (400 MHz, DMSO-d_6, H-H COSY): δ 7.98 (br s, 16H, N*H*), 3.07 (br s, 64H, C*H*$_2$CONHC*H*$_2$), 2.59–2.57 (m, 32H, C*H*$_2$NH$_2$), 2.54 (t, J = 6.8 Hz, 32H, CH$_2$C*H*$_2$S), 1.53–1.47 (m, 32H, SCH$_2$C*H*$_2$), 1.30 (br s, 24H, Si^{0,1}CH$_2$C*H*$_2$), 0.54 (br s, 80H, SiC*H*$_2$), –0.07 (s, 24H, Si^2*Me*), –0.10 (s, 12H, Si^1*Me*). ^{13}C {^{1}H} NMR (101 MHz, DMSO-d_6, HSQC, HMBC): δ 169.1 (*C*O), 42.3 (*C*H$_2$NH), 41.1 (*C*H$_2$NH$_2$),35.6 (S*C*H$_2$CH$_2$CH$_2$), 34.6 (*C*H$_2$CO), 23.6 (SCH$_2$*C*H$_2$), 18.3 (Si^1*C*H$_2$CH$_2$*C*H$_2$), 18.1 (Si^{1}CH$_2$*C*H$_2$CH$_2$), not detected (Si^0*C*H$_2$*C*H$_2$*C*H$_2$), 12.9(Si*C*H$_2$CH$_2$CH$_2$), –4.7 (Si^1*Me*), –5.1 (Si^2*Me*). ^{29}Si {^{1}H} NMR (79 MHz, DMSO-d_6): δ 2.55 (Si^2), 1.10 (Si^1), 0.85 (Si^0).

HRMS: (ESI+): *m/z* $[M + 2H]^{2+}$ calc. for $[C_{160}H_{350}N_{32}O_{16}S_{16}Si_{13}]^{2+}$ 1928.0049, found 1927.9992, $[M + 3H]^{3+}$ calc. for $[C_{160}H_{351}N_{32}O_{16}S_{16}Si_{13}]^{3+}$ 1285.6724 found 1285.6738.

CS-P

Data for **(10a)**: ^{1}H NMR (400 MHz, CDCl$_3$, H-H COSY): δ 6.13 (d, J = 4.6 Hz, 24H, C*H*$_{Ph}$), 3.91 (s, 36H, O*Me*), 3.62 (s, 72H, O*Me*), 3.12–2.86 (m, 8H, C*H*$_2$P), 1.37–1.26 (m, 8H, PCH$_2$C*H*$_2$), 1.25–1.15 (m, 8H, Si^{0}CH$_2$C*H*$_2$), 0.60–0.50 (m, 8H, C*H*$_2$CH$_2$CH$_2$P), 0.50–0.45 (m, 16H, Si^{0}C*H*$_2$CH$_2$C*H*$_2$), –0.15 (s, 24H, Si*Me*$_2$). ^{13}C {^{1}H} NMR (101 MHz, CDCl$_3$, HSQC, HMBC): δ 165.7 (d, $^4J_{(C-P)}$ = 2.4 Hz, *C*$_q$OMe), 163.8 (d, $^2J_{(C-P)}$ = 1.0 Hz, 2*C*$_q$OMe), 92.4 (d, $^1J_{(C-P)}$ = 103.8 Hz, P*C*$_q$), 91.3 (d, $^3J_{(C-P)}$ = 7.2 Hz, 2*C*H$_{Ph}$), 56.5 (2O*Me*), 56.4 (O*Me*), 32.0 (d, $^1J_{(C-P)}$ = 54.8 Hz, P*C*H$_2$), 20.3 (Si^{0}CH$_2$CH$_2$*C*H$_2$), 18.9 (d, $^2J_{(C-P)}$ = 5.7 Hz, *C*H$_2$CH$_2$P), 18.6

($Si^0CH_2CH_2$), 17.7 (d, $^3J_{(C\text{-}P)}$ = 17.6 Hz, $CH_2CH_2CH_2P$), 17.6 (Si^0CH_2), –3.3 ($SiMe_2$). ^{29}Si {^{1}H} NMR (79 MHz, $CDCl_3$): δ 1.43 (d, $^4J_{(Si\text{-}P)}$ = 2.9 Hz, Si^1), Si^0 was not detected. ^{31}P {^{1}H} NMR (162 MHz, $CDCl_3$): δ 5.07.

HRMS: (ESI+): *m/z* calc. for $[C_{140}H_{204}P_4Si_5O_{36}I_2]^{2+}$ 1490.5028, found 1490.5021, $[C_{140}H_{204}P_4Si_5O_{36}I]^{3+}$ 951.3669, found 951.3672, $[C_{140}H_{204}P_4Si_5O_{36}]^{4+}$ 681.7989, found 681.7972.

Data for **(10b)**: ^{1}H NMR (500 MHz, DMSO-d_6, H-H COSY): δ 6.28 (d, J = 4.7 Hz, 24H, CH_{Ph}), 3.83 (s, 36H, O*Me*), 3.55 (s, 72H, O*Me*), 3.05–2.89 (m, 8H, CH_2P), 1.33–1.22 (m, 8H, CH_2CH_2P), 1.20–1.11 (m, 8H, $Si^0CH_2CH_2$), 0.58–0.51 (m, 8H, $CH_2CH_2CH_2P$), 0.42–0.37 (m, 16H, $Si^0CH_2CH_2CH_2$), –0.20 (s, 24H, $SiMe_2$). ^{13}C {^{1}H} NMR (126 MHz, DMSO-d_6): δ 165.1 (d, $^4J_{(C\text{-}P)}$ = 1.4 Hz, C_qOMe), 163.4 (d, $^2J_{(C\text{-}P)}$ = 1.1 Hz, 2C_qOMe), 91.8 (d, $^1J_{(C\text{-}P)}$ = 103.9 Hz, PC_q), 91.3 (d, $^3J_{(C\text{-}P)}$ = 7.0 Hz, 2CH_{Ph}), 56.0 (2O*Me*), 55.7 (O*Me*), 30.8 (d, $^1J_{(C\text{-}P)}$ = 54.9 Hz, PCH_2), 19.3 ($Si^0CH_2CH_2CH_2$), 18.4 (d, $^2J_{(C\text{-}P)}$ = 3.1 Hz, CH_2CH_2P), 18.0 ($Si^0CH_2CH_2$), 16.8 (Si^0CH_2), 16.5 (d, $^3J_{(C\text{-}P)}$ = 17.2 Hz, $CH_2CH_2CH_2P$), –3.6 ($SiMe_2$). ^{29}Si {^{1}H} NMR (79 MHz, DMSO-d_6): δ 1.49 (d, $^4J_{(Si\text{-}P)}$ = 2.6 Hz, Si^1), 0.66 (Si^0). ^{31}P {^{1}H} NMR (162 MHz, DMSO-d_6): δ 5.17.

HRMS: (ESI+): *m/z* calc. for $[C_{140}H_{204}O_{36}P_4Si_5Cl_3]^+$ 2833.1018, found 2833.1134, $[C_{140}H_{204}O_{36}P_4Si_5Cl_2]^{2+}$ 1399.0665, found 1399.0670, $[C_{140}H_{204}O_{36}P_4Si_5Cl]^{3+}$ 920.7213, found 920.7215, $[C_{140}H_{204}O_{36}P_4Si_5]^{4+}$ 681.7989, found 681.7997.

Data for **(11a)**: ^{1}H NMR (400 MHz, $CDCl_3$, H-H COSY): δ 6.13 (d, J = 4.6 Hz, 48H, CH_{Ph}), 3.90 (s, 72H, O*Me*), 3.62 (s, 144H, O*Me*), 3.02–2.95 (m, 16H, CH_2P), 1.37–1.26 (m, 16H, CH_2CH_2P), 1.27–1.21 (m, 24H, $Si^1CH_2CH_2$), 0.56–0.52 (m, 16H, $CH_2CH_2CH_2P$), 0.48–0.43 (m, 48H, $SiCH_2$), –0.14 (s, 12H, Si^1Me), –0.16 (s, 48H, Si^2Me_2). ^{13}C {^{1}H} NMR (101 MHz, $CDCl_3$, HSQC, HMBC): δ 165.7 (d, $^4J_{(C\text{-}P)}$ = 1.3 Hz, C_qOMe), 163.8 (d, $^2J_{(C\text{-}P)}$ = 1.1 Hz,

2C_qOMe), 92.4 (d, $^1J_{(C\text{-}P)}$ = 103.6 Hz, PC_q), 91.3 (d, $^3J_{(C\text{-}P)}$ = 7.0 Hz, 2CH$_{Ph}$), 56.6 (2O*Me*), 56.5 (O*Me*), 32.0 (d, $^1J_{(C\text{-}P)}$ = 55.4 Hz, PCH$_2$), 20.2 (Si^{1}CH$_2$CH$_2$$CH_2$Si^2), 19.3 (Si^{0}CH$_2CH_2$$CH_2$), 18.9 (Si^1$CH_2CH_2CH_2$Si^2), 18.9 (d, $^2J_{(C\text{-}P)}$ = 4.8 Hz, CH$_2$CH$_2$P), 18.7 (Si^{0}CH$_2$$CH_2$), 18.5 (Si^{1}CH$_2$$CH_2CH_2$Si^2), 17.7 (d, $^3J_{(C\text{-}P)}$ = 16.6 Hz, CH$_2$CH$_2$CH$_2$P), overlapped (Si^0CH$_2$), –3.2 (Si^2Me_2), –4.9 (Si^1*Me*). ^{29}Si {^{1}H} NMR (79 MHz, CDCl$_3$): δ 1.42 (d, $^4J_{(Si\text{-}P)}$ = 3.2 Hz, Si^2), 0.84 (Si^1), not detected (Si^0). ^{31}P {^{1}H} NMR (162 MHz, CDCl$_3$): δ 5.08.

HRMS: (ESI+): *m/z* calc. for [$C_{296}H_{444}$ $O_{72}P_8Si_{13}I_5$]$^{3+}$ 2133.709, found 2133.710, [$C_{296}H_{444}$ $O_{72}P_8Si_{13}I_4$]$^{4+}$ 1568.556, found 1568.555, [$C_{296}H_{444}$ $O_{72}P_8Si_{13}I_3$]$^{5+}$ 1229.4636, found 1229.4653, [$C_{296}H_{444}$ $O_{72}P_8Si_{13}I_2$]$^{6+}$ 1003.4021, found 1003.4018, [$C_{296}H_{444}$ $O_{72}P_8Si_{13}I$]$^{7+}$ 841.9296, found 841.9296.

Data for **(11b)**: ^{1}H NMR (400 MHz, DMSO-d_6, H-H COSY): δ 6.28 (d, J = 4.6 Hz, 48H, CH_{Ph}), 3.83 (s, 72H, O*Me*), 3.55 (s, 144H, O*Me*), 3.00–2.94 (m, 16H, CH_2P), 1.27–1.15 (m, 40H,SiCH$_2$CH_2), 0.56–0.52 (m, 16H, CH_2CH$_2$CH$_2$P), 0.43–0.36 (m, 48H, SiCH_2), –0.21 (s, 12H, Si^1*Me*), –0.22 (s, 48H, Si^2Me_2). ^{13}C {^{1}H} NMR (101 MHz, DMSO-d_6, HSQC, HMBC): δ 165.1 (d, $^4J_{(C\text{-}P)}$ = 1.8 Hz, C_qOMe), 163.4 (d, $^2J_{(C\text{-}P)}$ = 1.6 Hz, 2C_qOMe), 91.8 (d, $^1J_{(C\text{-}P)}$ = 101.7 Hz, PC_q), 91.3 (d, $^3J_{(C\text{-}P)}$ = 7.4 Hz, 2CH$_{Ph}$), 56.0 (2O*Me*), 55.7 (O*Me*), 30.8 (d, $^1J_{(C\text{-}P)}$ = 55.1 z, PCH$_2$), 19.3 (Si^{1}CH$_2$CH$_2$$CH_2$Si^2), not detected or overlapped (Si^0CH$_2$$CH_2$$CH_2$), 18.4 (d, $^2J_{(C\text{-}P)}$ = 5.7 Hz, CH$_2$CH$_2$P), 18.2 (Si^1CH$_2$CH$_2$CH$_2$Si^2), 17.9 (Si^{1}CH$_2$$CH_2CH_2$Si^2), 16.5 (d, $^3J_{(C\text{-}P)}$ = 17.6 Hz, CH$_2$CH$_2$CH$_2$P), –3.6 (Si^2Me_2), –5.1 (Si^1*Me*). ^{29}Si {^{1}H} NMR (79 MHz, DMSO-d_6): δ 1.42 (d, $^4J_{(Si\text{-}P)}$ = 3.0 Hz, Si^2), 0.97 (Si^1), 0.73 (Si^0). ^{31}P {^{1}H} NMR (162 MHz, DMSO-d_6): δ 5.17.

HRMS: (ESI+): *m/z* calc. for [$C_{296}H_{444}O_{72}P_8Si_{13}Cl_5$]$^{3+}$ 1981.4824, found 1981.4870, [$C_{296}H_{444}O_{72}P_8Si_{13}Cl_4$]$^{4+}$ 1477.1196, found 1477.1182, [$C_{296}H_{444}O_{72}P_8Si_{13}Cl_3$]$^{5+}$ 1174.1719, found 1174.1721, [$C_{296}H_{444}O_{72}P_8Si_{13}Cl_2$]$^{6+}$ 972.9234, found 972.9244, [$C_{296}H_{444}O_{72}P_8Si_{13}Cl$]$^{7+}$ 826.9389, found 826.9384, [$C_{296}H_{444}O_{72}P_8Si_{13}$]$^{8+}$ 720.8253, found 720.8253.

Data for **(12a)**: ^{1}H NMR (400 MHz, acetonitrile-d_3, H-H COSY): δ 6.21 (d, J = 4.6 Hz, 96H, CH_{Ph}), 3.84 (s, 144H, O*Me*), 3.57 (s, 288H, O*Me*), 3.07–2.99 (m, 32H, CH_2P), 1.37–1.22 (m, 88H, SiCH$_2$CH_2), 0.59–0.55 (m, 32H, CH_2CH$_2$CH$_2$P), 0.48–0.40 (m, 112H, SiCH_2), –0.18 (br s, 132H, $Si^{1,2}$*Me*, Si^3*Me*$_2$). ^{13}C {^{1}H} NMR (101 MHz, acetonitrile-d_3, HSQC, HMBC): δ 166.6 (d, $^4J_{(C\text{-}P)}$ = 1.8 Hz, C_qOMe), 164.8 (d, $^2J_{(C\text{-}P)}$ = 1.3 Hz, 2C_qOMe), 93.3 (d, $^1J_{(C\text{-}P)}$ = 103.5 Hz, PC_q), 92.2 (d, $^3J_{(C\text{-}P)}$ = 6.9 Hz, 2CH_{Ph}), 57.0 (2O*Me*), 56.8 O*Me*), 32.4 (d, $^1J_{(C\text{-}P)}$ = 55.0 Hz, PCH$_2$), 20.6 (Si^2CH$_2$CH$_2$$CH_2$$Si^3$), overlapped or not detected ($Si^0$$CH_2$$CH_2$$CH_2$), 19.8 ($CH_2CH_2$P), 19.7 ($Si^2$$CH_2CH_2CH_2$$Si^3$), 19.5 ($Si^2CH_2$$CH_2CH_2$$Si^3$), 19.4 ($Si^1CH_2$$CH_2CH_2$$Si^2$), 19.3 ($Si^1$$CH_2CH_2$$CH_2$$Si^2$), 17.8 (d, $^3J_{(C\text{-}P)}$ = 18.3 Hz, CH$_2$CH$_2$CH$_2$P), –3.0 ($Si^{2,3}$*Me*), –4.3 (Si^1*Me*). ^{29}Si {^{1}H} NMR (79 MHz, acetonitrile-d_3): δ 1.35 (d, $^4J_{(Si\text{-}P)}$ = 3.1 Hz, Si^3), 0.89 ($Si^{1,2}$), not detected (Si^0). ^{31}P {^{1}H} NMR (162 MHz, acetonitrile-d_3): δ 5.08.

HRMS: (ESI+): *m/z* calc. for $[C_{608}H_{924}O_{144}P_{16}Si_{29}I_{11}]^{5+}$ 2648.6751, found 2648.6842, $[C_{608}H_{924}O_{144}P_{16}Si_{29}I_{10}]^{6+}$ 2186.0784, found 2185.98804, $[C_{608}H_{924}O_{144}P_{16}Si_{29}I_{9}]^{7+}$ 1855.6523, found 1855.6511, $[C_{608}H_{924}O_{144}P_{16}Si_{29}I_{8}]^{8+}$ 1607.8326, found 1607.7982, $[C_{608}H_{924}O_{144}P_{16}Si_{29}I_{7}]^{9+}$ 1415.0839, found 1415.0792, $[C_{608}H_{924}O_{144}P_{16}Si_{29}I_{6}]^{10+}$ 1260.8851, found 1260.8124.

Data for **(12b)**: ^{1}H NMR (400 MHz, DMSO-d_6, H-H COSY): δ 6.28 (d, J = 4.6 Hz, 96H, CH_{Ph}), 3.83 (s, 144H, O*Me*), 3.55 (s, 288H, O*Me*), 2.97 (br s, 32H, CH_2P), 1.37–1.22 (m, 88H, SiCH$_2$CH_2), 0.53 (br s, 32H, CH_2CH$_2$CH$_2$P), 0.39 (br s, 112H, SiCH_2), –0.22 (br s, 132H, $Si^{1,2}$*Me*, Si^3*Me*$_2$). ^{13}C {^{1}H} NMR (101 MHz, DMSO-d_6, HSQC, HMBC): δ 165.1 d, $^4J_{(C\text{-}P)}$ = 1.8 Hz, C_qOMe), 163.4 (d, $^2J_{(C\text{-}P)}$ = 1.5 Hz, 2C_qOMe), 91.7 (d, $^1J_{(C\text{-}P)}$ = 102.0 Hz, PC_q), 91.3 (d, $^3J_{(C\text{-}P)}$ = 6.9 Hz, 2CH_{Ph}), 56.0 (s, 2O*Me*), 55.7 (m, O*Me*), 30.9 (d, $^1J_{(C\text{-}P)}$ = 53.8 Hz, PCH$_2$), 19.3 (Si^2CH$_2$CH$_2$$CH_2$$Si^3$), not detected ($Si^0$$CH_2$$CH_2$$CH_2$), 18.3 ($Si^2$$CH_2CH_2CH_2$$Si^3CH_2$$CH_2$), 18.1 ($CH_2CH_2$$Si^2CH_2$$CH_2$), 17.9 ($Si^1$$CH_2CH_2$$CH_2$$Si^2$), 16.5 (d, $^3J_{(C\text{-}P)}$ = 17.3 Hz, CH$_2$CH$_2$CH$_2$P), –

3.6 (Si^{2,3}*Me*), –5.1 (Si^{1}*Me*). ^{29}Si {^{1}H} NMR (79 MHz, DMSO-d_6): δ 1.39 (d, $^{4}J_{(Si\text{-}P)}$ = 2.2 Hz, Si^{3}), 0.94 (Si^{1,2}), not detected (Si^{0}). ^{31}P {^{1}H} NMR (162 MHz, DMSO-d_6): δ 5.16.

HRMS: (ESI+): *m/z* calc. for $[C_{608}H_{924}O_{144}P_{16}Si_{29}Cl_8]^{8+}$ 1516.3963, found 1516.2778, $[C_{608}H_{924}O_{144}P_{16}Si_{29}Cl_7]^{9+}$ 1343.9113, found 1343.9554, $[C_{608}H_{924}O_{144}P_{16}Si_{29}Cl_3]^{13+}$ 919.4866, found 919.4220.

Data for **(13a)**: ^{1}H NMR (400 MHz, $CDCl_3$, H-H COSY): δ 7.37 (d, $^{3}J_{(H\text{-}P)}$ = 12.5 Hz, 24H, CH_{Ph}), 3.82 (s, 36H, O*Me*), 3.49–3.40 (m, 8H, CH_2P), 2.37 (s, 72H, CH_3), 1.60–1.55 (m, 8H, CH_2CH$_2$P), 1.23–1.16 (m, 8H, Si^{0}CH$_2$CH_2), 0.87–0.83 (m, 8H, CH_2CH$_2$CH$_2$P), 0.50–0.42 (m, 16H, Si^{0}CH_2CH$_2$CH_2), –0.14 (s, 24H, Si^{1}*Me*$_2$). ^{13}C {^{1}H} NMR (101 MHz, $CDCl_3$, HSQC, HMBC): δ 162.6 (d, $^{4}J_{(C\text{-}P)}$ = 3.4 Hz, C_qOMe), 134.1 (d, $^{3}J_{(C\text{-}P)}$ = 15.3 Hz, 2C_q), 134.0 (d, $^{2}J_{(C\text{-}P)}$ = 12.0 Hz,CH$_{Ph}$), 113.2 (d, $^{1}J_{(C\text{-}P)}$ = 87.8 Hz, PC_q), 60.2 (O*Me*), 26.9 (d, $^{1}J_{(C\text{-}P)}$ = 48.3 Hz, CH$_2$P), 20.1 (Si^{0}CH$_2$CH$_2$$CH_2$), 18.4 (Si^{0}CH$_2$$CH_2$), 17.7 (d, $^{2}J_{(C\text{-}P)}$ = 4.4 Hz, CH$_2$CH$_2$P), 17.5 (Si^{0}CH$_2$), 17.2 (d, $^{3}J_{(C\text{-}P)}$ = 9.8 Hz, CH$_2$CH$_2$CH$_2$P), 16.7 (*Me*), –3.4 (Si*Me*$_2$). ^{29}Si {^{1}H} NMR (79 MHz, DMSO-d_6): δ 1.59 (d, $^{4}J_{(Si\text{-}P)}$ = 2.4 Hz, Si^{1}), 0.46 (Si^{0}). ^{31}P {^{1}H} NMR (162 MHz, $CDCl_3$): δ 3.84.

HRMS: (ESI+): *m/z* calc. for $[C_{140}H_{204}O_{12}P_4Si_5I_3]^{+}$ 2723.0306, found 2723.0303, $[C_{140}H_{204}O_{12}P_4Si_5I_2]^{2+}$ 1298.0628, found 1298.0629, $[C_{140}H_{204}O_{12}P_4Si_5I]^{3+}$ 823.0735, found 823.0746, $[C_{140}H_{204}O_{12}P_4Si_5]^{4+}$ 585.5789, found 585.5780.

Data for **(13b)**: ^{1}H NMR (400 MHz, DMSO-d_6, H-H COSY): δ 7.49 (d, $^{3}J_{(H\text{-}P)}$ = 12.6 Hz, 24H, CH_{Ph}), 3.74 (s, 36H, O*Me*), 3.40–3.31 (m, 8H, CH_2P), 2.27 (s, 72H, *Me*), 1.45–1.40 (m, 8H, CH_2CH$_2$P), 1.18–1.10 (m, 8H, Si^{0}CH$_2$CH_2), 0.64–0.60 (m, 8H, CH_2CH$_2$CH$_2$P), 0.39–0.36 (m, 16H, SiCH_2), –0.19 (s, 24H, Si*Me*$_2$). ^{13}C {^{1}H} NMR (101 MHz, DMSO-d_6, HSQC, HMBC): δ 161.7 (d, $^{4}J_{(C\text{-}P)}$ = 3.4 Hz, C_qOMe), 133.9 (d, $^{2}J_{(C\text{-}P)}$ = 11.0 Hz, 2CH$_{Ph}$), 133.0 (d, $^{3}J_{(C\text{-}P)}$ = 14.3 Hz, $C_{q(Ph)}$), 113.5 (d, $^{1}J_{(C\text{-}P)}$ = 87.7 Hz, PC_q), 59.6 (O*Me*), 23.7 (d, $^{1}J_{(C\text{-}P)}$ = 47.6 Hz, PCH$_2$),

19.2 ($Si^0CH_2CH_2\mathit{C}H_2$), 18.0 ($Si^0CH_2\mathit{C}H_2$), 16.9 ($Si^0\mathit{C}H_2$), 16.8 (d, $^2J_{(C\text{-}P)}$ = 3.6 Hz, $\mathit{C}H_2CH_2P$), 16.3 (d, $^3J_{(C\text{-}P)}$ = 13.1 Hz, $\mathit{C}H_2CH_2CH_2P$), 16.0 (*Me*), –3.8 ($Si^1\mathit{Me}_2$). ^{29}Si {^{1}H} NMR (79 MHz, DMSO-d_6): δ 1.60 (d, $^4J_{(Si\text{-}P)}$ = 2.6 Hz, Si^1), 0.60 (Si^0). ^{31}P {^{1}H} NMR (162 MHz, DMSO-d_6): δ 3.37.

HRMS: (ESI+): *m/z* calc. for $[C_{140}H_{204}O_{12}P_4Si_5Cl_2]^{2+}$ 1206.6272, found 1206.6270, $[C_{140}H_{204}O_{12}P_4Si_5Cl_1]^{3+}$ 792.7619, found 792.7617, $[C_{140}H_{204}O_{12}P_4Si_5]^{4+}$ 585.5789, found 585.5777.

Data for **(14a)**: ^{1}H NMR (400 MHz, $CDCl_3$, H-H COSY): δ 7.37 (d, $^3J_{(H\text{-}P)}$ = 12.5 Hz, 48H, $C\mathit{H}_{Ph}$), 3.81 (s, 72H, O*Me*), 3.47–3.39 (m, 16H, $C\mathit{H}_2P$), 2.36 (s, 144H, *Me*), 1.62–1.51 (m, 16H, $C\mathit{H}_2CH_2P$), 1.23–1.16 (m, 24H, $SiCH_2C\mathit{H}_2$), 0.86–0.82 (m, 16H, $C\mathit{H}_2CH_2CH_2P$), 0.47–0.42 (m, 48H, $SiC\mathit{H}_2$), –0.15 (s, 48H, $Si^2\mathit{Me}_2$), –0.17 (s, 12H, $Si^1\mathit{Me}$). ^{13}C {^{1}H} NMR (101 MHz, $CDCl_3$, HSQC, HMBC): δ 162.6 (d, $^4J_{(C\text{-}P)}$ = 3.4 Hz, C_qOMe), 134.1 (d, $^3J_{(C\text{-}P)}$ = 14.2 Hz, $\mathit{C}_{q(Ph)}$), 134.0 (d, $^2J_{(C\text{-}P)}$ = 11.2 Hz, $\mathit{C}H_{Ph}$), 113.2 (d, $^1J_{(C\text{-}P)}$ = 81.5 Hz, $P\mathit{C}_q$), 60.2 (O*Me*), 26.9 (d, $^1J_{(C\text{-}P)}$ = 48.6 Hz, $P\mathit{C}H_2$), 20.0 ($Si^1CH_2CH_2\mathit{C}H_2Si^2$), 19.3 ($Si^0CH_2CH_2\mathit{C}H_2$), 18.8 ($Si^1\mathit{C}H_2CH_2CH_2Si^2$), 18.6 ($Si^0CH_2\mathit{C}H_2$), 18.4 ($Si^1CH_2\mathit{C}H_2CH_2Si^2$), 17.7 (d, $^2J_{(C\text{-}P)}$ = 4.5 Hz, $\mathit{C}H_2CH_2P$) overlapped ($Si^0\mathit{C}H_2$), 17.2 (d, $^3J_{(C\text{-}P)}$ = 12.5 Hz, $\mathit{C}H_2CH_2CH_2P$), 16.8 (*Me*), –3.3 ($Si^2\mathit{Me}_2$), –4.9 ($Si^1\mathit{Me}$). ^{29}Si {^{1}H} NMR (79 MHz, DMSO-d_6): δ 1.62 (d, $^4J_{(Si\text{-}P)}$ = 2.6 Hz, Si^2), 0.85 (Si^1), not detected Si^0. ^{31}P {^{1}H} NMR (162 MHz, $CDCl_3$): δ 3.88.

HRMS: (ESI+): *m/z* calc. for $[C_{296}H_{444}O_{24}P_8Si_{13}I_5]^{3+}$ 1877.7906, found 1877.7913, $[C_{296}H_{444}O_{24}P_8Si_{13}I_4]^{4+}$ 1376.6167, found 1376.6149, $[C_{296}H_{444}O_{24}P_8Si_{13}I_3]^{5+}$ 1075.9123, found 1075.9122, $[C_{296}H_{444}O_{24}P_8Si_{13}I_2]^{6+}$ 857.4428, found 857.4445, $[C_{296}H_{444}O_{24}P_8Si_{13}I]^{8+}$ 732.2502, found 732.2495.

Data for **(14b)**: ^{1}H NMR (400 MHz, DMSO-d_6, H-H COSY): δ 7.52 (d, $^3J_{(H\text{-}P)}$ = 12.5 Hz, 48H, $C\mathit{H}_{Ph}$), 3.74 (s, 72H, O*Me*), 3.44–3.41 (m, 16H, $C\mathit{H}_2P$), 2.26 (s, 144H, *Me*), 1.42 (br s, 16H,

CH_2CH$_2$P), 1.19–1.14 (m, 24H, SiCH$_2$CH_2), 0.66–0.62 (m, 16H, CH_2CH$_2$CH$_2$P), 0.41–0.36 (m, 48H, SiCH_2), –0.20 (s, 48H, Si^2*Me*$_2$), –0.25 (s, 12H, Si^1*Me*). ^{13}C {^{1}H} NMR (101 MHz, DMSO-d_6, HSQC, HMBC): δ 161.7 (d, $^4J_{(C\text{-}P)}$ = 3.4 Hz, C_qOMe), 133.9 (d, $^2J_{(C\text{-}P)}$ = 11.0 Hz, CH$_{Ph}$), 133.0 (d, $^3J_{(C\text{-}P)}$ = 14.3 Hz, $C_{q(Ph)}$), 113.6 (d, $^1J_{(C\text{-}P)}$ = 87.6 Hz, PC_q), 59.6 (O*Me*), 23.8 (d, $^1J_{(C\text{-}P)}$ = 47.9 Hz, PCH$_2$), 19.1 (Si^{1}CH$_2$CH$_2$$CH_2$Si^2), 18.4 (Si^{0}CH$_2CH_2$$CH_2$), 18.2 (Si^1$CH_2CH_2CH_2$Si^2), overlapped (Si^{0}CH$_2$$CH_2$), 17.9 (Si^{1}CH$_2$$CH_2CH_2$Si^2), 17.0 (Si^0$CH_2$), 16.8 (d, $^2J_{CP}$ = 3.6 Hz, CH$_2$CH$_2$P), 16.2 (d, $^3J_{CP}$ = 13.2 Hz, CH$_2$CH$_2$CH$_2$P), 16.0 (*Me*), –3.8 (Si^2*Me*$_2$), –5.2 (Si^1*Me*). ^{29}Si {^{1}H} NMR (79 MHz, DMSO-d_6): δ 1.52 (d, $^4J_{(Si\text{-}P)}$ = 2.7 Hz, Si^2), 0.93 (Si^1), 0.73 (Si^0). ^{31}P {^{1}H} NMR (162 MHz, DMSO-d_6): δ 3.44.

HRMS: (ESI+): *m/z* calc. for $[C_{296}H_{444}O_{24}P_8Si_{13}Cl_6]^{2+}$ 2605.8299, found 2605.8178, $[C_{296}H_{444}O_{24}P_8Si_{13}Cl_5]^{3+}$ 1725.5637, found 1725.5648, $[C_{296}H_{444}O_{24}P_8Si_{13}Cl_4]^{4+}$ 1285.1806, found 1285.1816, $[C_{296}H_{444}O_{24}P_8Si_{13}Cl_3]^{5+}$ 1021.1508, found 1021.1523, $[C_{296}H_{444}O_{24}P_8Si_{13}Cl_2]^{6+}$ 844.9641, found 844.9657.

Data for **(15a)**: ^{1}H NMR (400 MHz, CDCl$_3$, H-H COSY): δ 7.36 (d, $^3J_{(H\text{-}P)}$ = 12.6 Hz, 96H, CH_{Ph}), 3.80 (s, 144H, O*Me*), 3.46–3.39 (m, 32H, CH_2P), 2.34 (s, 288H, *Me*), 1.61–1.50 (m, 32H, CH_2CH$_2$P), 1.24–1.15 (m, 56H, SiCH$_2$CH_2), 0.88–0.81 (m, 32H, CH_2CH$_2$CH$_2$P), 0.47–0.41 (m, 112H, Si), –0.16 (s, 120H, Si^{2,3}*Me*), –0.18 (s, 12H, Si^1*Me*). ^{13}C {^{1}H} NMR (101 MHz, CDCl$_3$, HSQC, HMBC): δ 162.6 (d, $^4J_{(C\text{-}P)}$ = 3.4 Hz, C_qOMe), 134.1 (d, $^3J_{(C\text{-}P)}$ = 13.7 Hz, $C_{q(Ph)}$), 134.0 (d, $^2J_{(C\text{-}P)}$ = 10.5 Hz, CH$_{Ph}$), 113.2 (d, $^1J_{(C\text{-}P)}$ = 87.8 Hz, PC_q), 60.2 (O*Me*), 26.9 (d, $^1J_{(C\text{-}P)}$ = 48.2 Hz, PCH$_2$), 19.9 (Si^{2}CH$_2$CH$_2$$CH_2$Si^3), 18.8 (Si^2$CH_2CH_2CH_2$Si^3), 18.6 (Si^{1}CH$_2$$CH_2CH_2$Si^2), 18.4 (Si^1$CH_2CH_2$$CH_2$Si^{2}CH$_2$$CH_2CH_2$Si^3), 17.7 (d, $^2J_{(C\text{-}P)}$ = 4.5 Hz, CH$_2$CH$_2$P), 17.2 (d, $^3J_{(C\text{-}P)}$ = 12.1 Hz, CH$_2$CH$_2$CH$_2$P), not detected (Si^0CH$_2$$CH_2$$CH_2$), 16.8 (*Me*), –3.4 (Si^{3,2}*Me*), –4.9 (Si^1*Me*). ^{29}Si {^{1}H} NMR (79 MHz, DMSO-d_6): δ 1.61 (d, $^4J_{(Si\text{-}P)}$ = 2.5 Hz, Si^3), 0.85 (Si^{1,2}), not detected Si^0. ^{31}P {^{1}H} NMR (162 MHz, CDCl$_3$): δ 3.90.

HRMS: (ESI+): *m/z* calc. for $[C_{608}H_{924}O_{48}P_{16}Si_{29}I_{11}]^{5+}$ 2341.3724, found 2341.3745, $[C_{608}H_{924}O_{48}P_{16}Si_{29}I_{10}]^{6+}$ 1929.9928, found 1930.0001, $[C_{608}H_{924}O_{48}P_{16}Si_{29}I_{9}]^{7+}$ 1636.1503, found 1636.1493.

Data for **(15b)**: ^{1}H NMR (400 MHz, DMSO-d_6, H-H COSY): δ 7.54 (d, $^{3}J_{(H\text{-}P)}$ = 12.5 Hz, 96H, CH_{Ph}), 3.71 (s, 144H, O*Me*), 3.56–3.51 (m, 32H, CH_2P), 2.26 (s, 288H, *Me*), 1.41 (br s, 32H, CH_2CH$_2$P), 1.21–1.13 (m, 56H, SiCH$_2$CH_2), 0.68–0.63 (m, 32H, CH_2CH$_2$CH$_2$P), 0.43–0.36 (m, 112H, SiCH_2), –0.22 (br s, 132H, Si^{1,2,3}*Me*). ^{13}C {^{1}H} NMR (101 MHz, DMSO-d_6, HSQC, HMBC): δ 161.6 (d, $^{4}J_{(C\text{-}P)}$ = 3.3 Hz, C_qOMe), 133.9 (d, $^{2}J_{(C\text{-}P)}$ = 11.0 Hz, CH$_{Ph}$), 132.9 (d, $^{3}J_{(C\text{-}P)}$ = 13.8 Hz, $C_{q(Ph)}$), 113.6 (d, $^{1}J_{(C\text{-}P)}$ = 87.7 Hz, PC_q), 59.5 (O*Me*), 23.8 (d, $^{1}J_{(C\text{-}P)}$ = 47.6 Hz, PCH$_2$), 19.2 (Si^{2}CH$_2$CH$_2$$CH_2$Si^{3}), 18.3/18.01, (Si^{1}CH$_2$$CH_2$$CH_2$Si^{2}), 18.13 (Si^{2}$CH_2CH_2CH_2$Si^{3}), not detected (Si^{0}CH$_2$$CH_2$$CH_2$), 17.9 (Si^{2}CH$_2$$CH_2CH_2$Si^{3}), 16.8 (d, $^{2}J_{(C\text{-}P)}$ = 3.6 Hz, CH$_2$CH$_2$P), 16.2 (d, $^{3}J_{(C\text{-}P)}$ = 13.2 Hz, CH$_2$CH$_2$CH$_2$P), 15.9 (*Me*), –3.8 (Si^{2,3}*Me*), –5.2 (Si^{1}*Me*). ^{29}Si {^{1}H} NMR (79 MHz, DMSO-d_6): δ 1.48 (br s, Si^{3}), 0.91 (Si^{1,2}), 0.61 (Si^{0}). ^{31}P {^{1}H} NMR (162 MHz, DMSO-d_6): δ 3.48.

2. NMR spectra of the compounds 1c – 15b

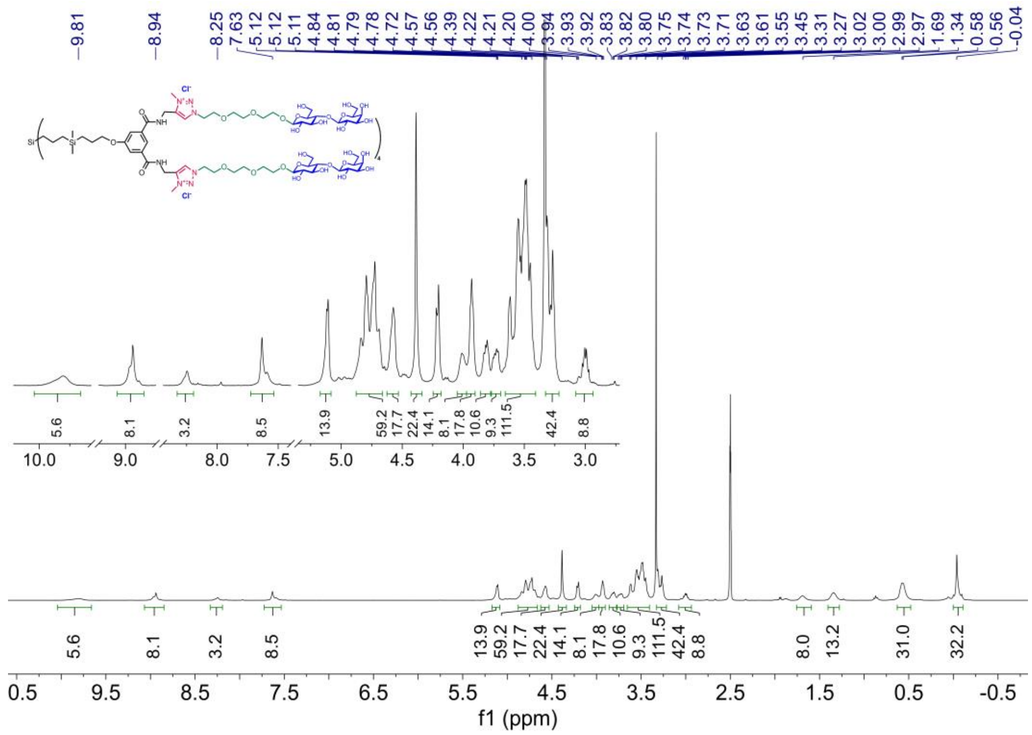

Figure S1: ^{1}H NMR (400 MHz, DMSO-d_6) **1c**

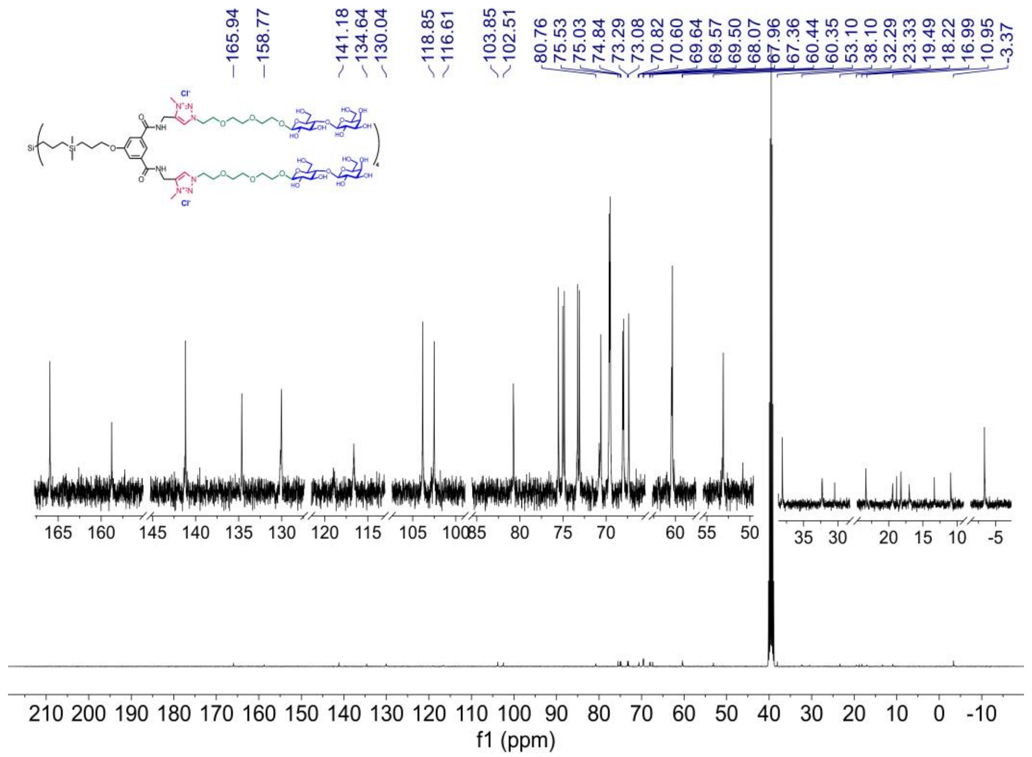

Figure S2: ^{13}C {^{1}H} NMR (101 MHz, DMSO-d_6) **1c**

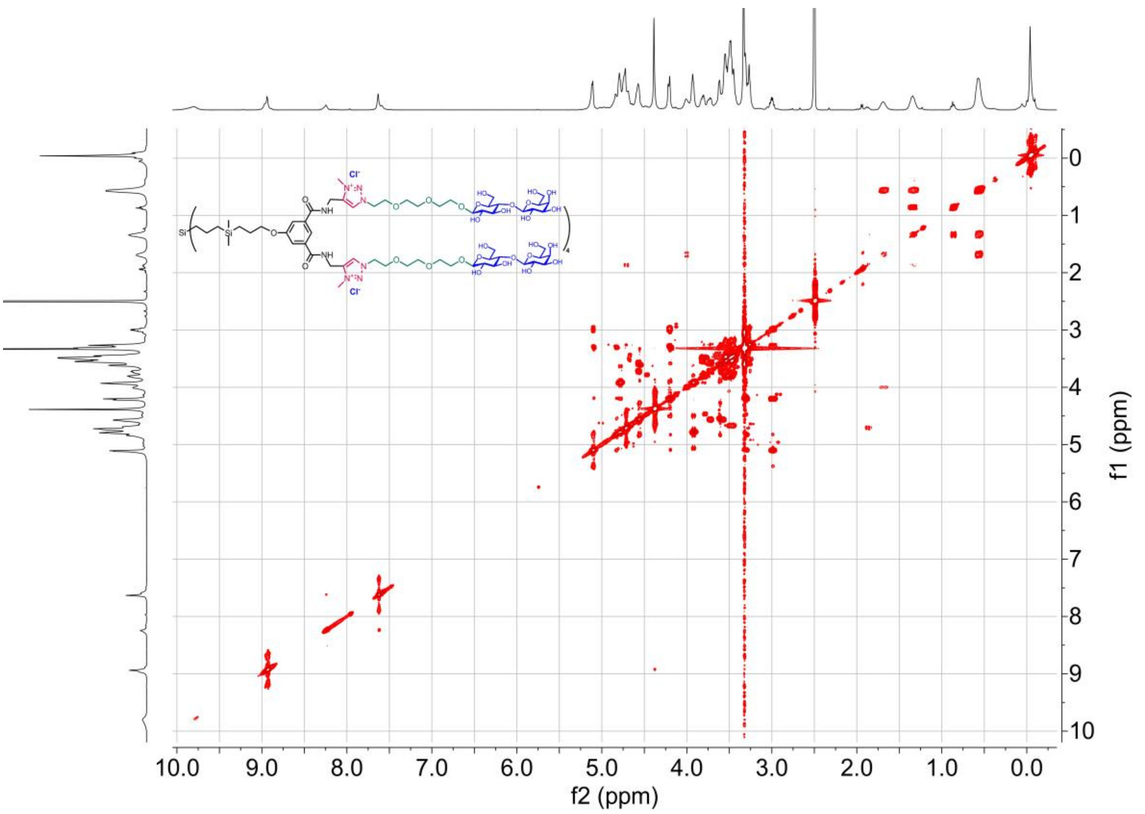

Figure S3: ^{1}H-^{1}H COSY NMR (DMSO-d_6) **1c**

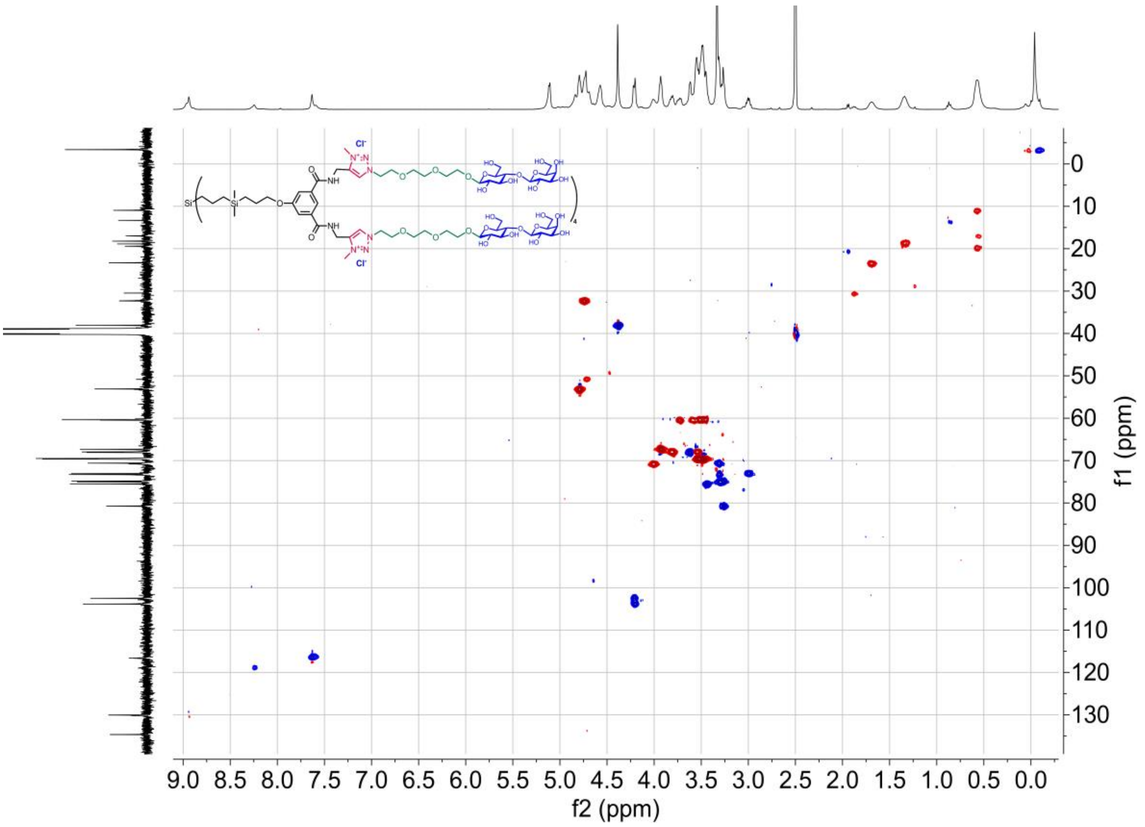

Figure S4: ^{1}H-^{13}C HSQC NMR (DMSO-d_6) **1c**

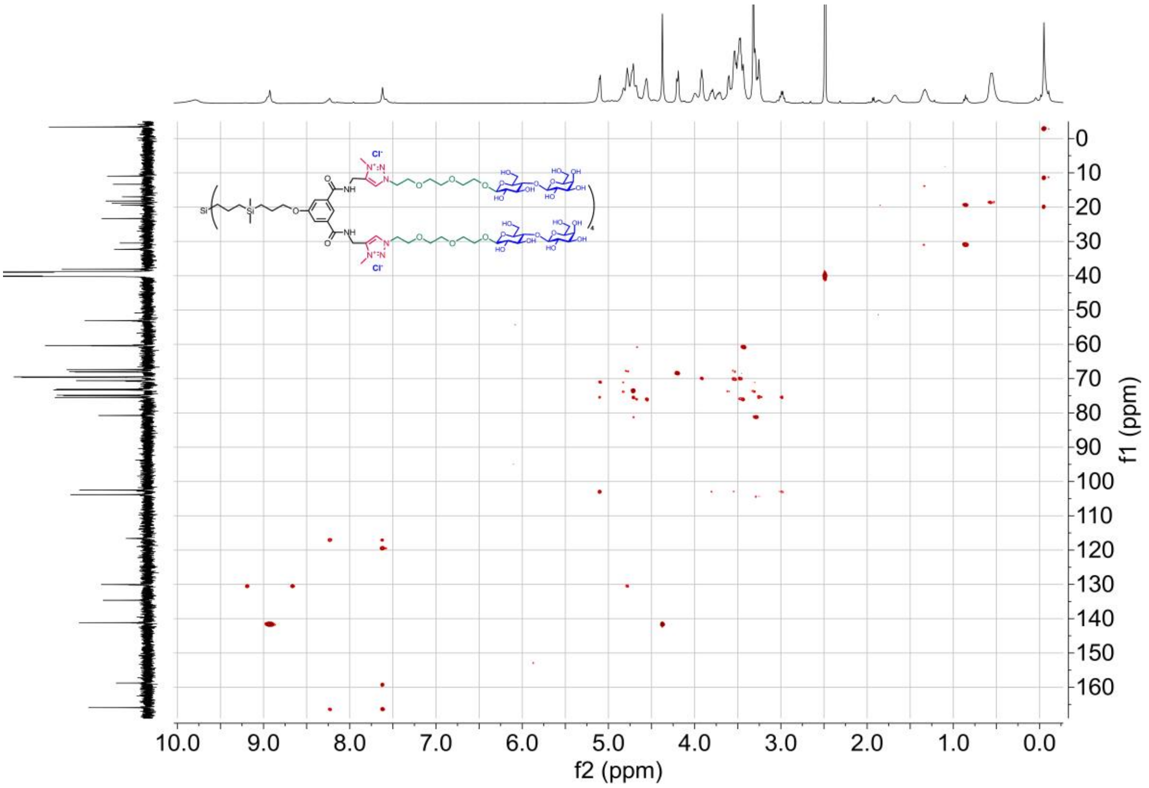

Figure S5: ^{1}H-^{13}C HMBC NMR (DMSO-d_6) **1c**

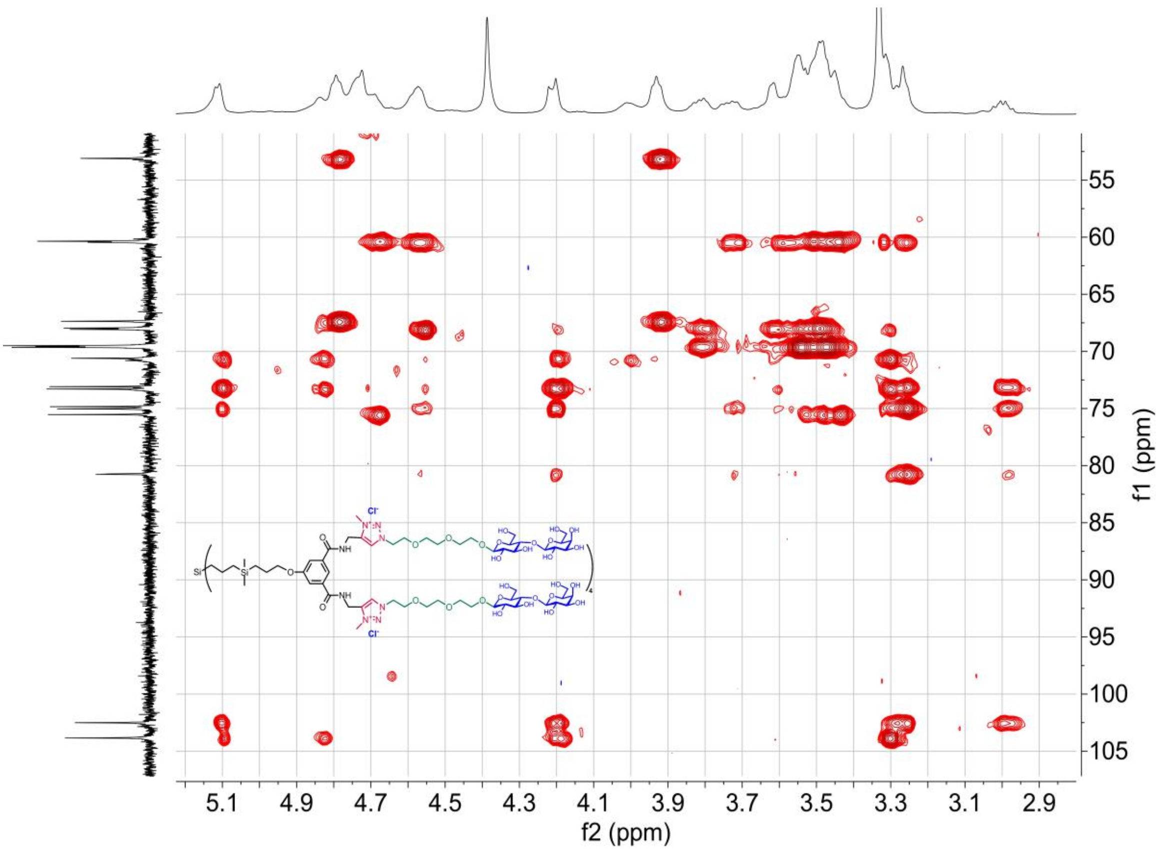

Figure S6: ^{1}H-^{13}C HSQC TOCSY NMR (DMSO-d_6) **1c**

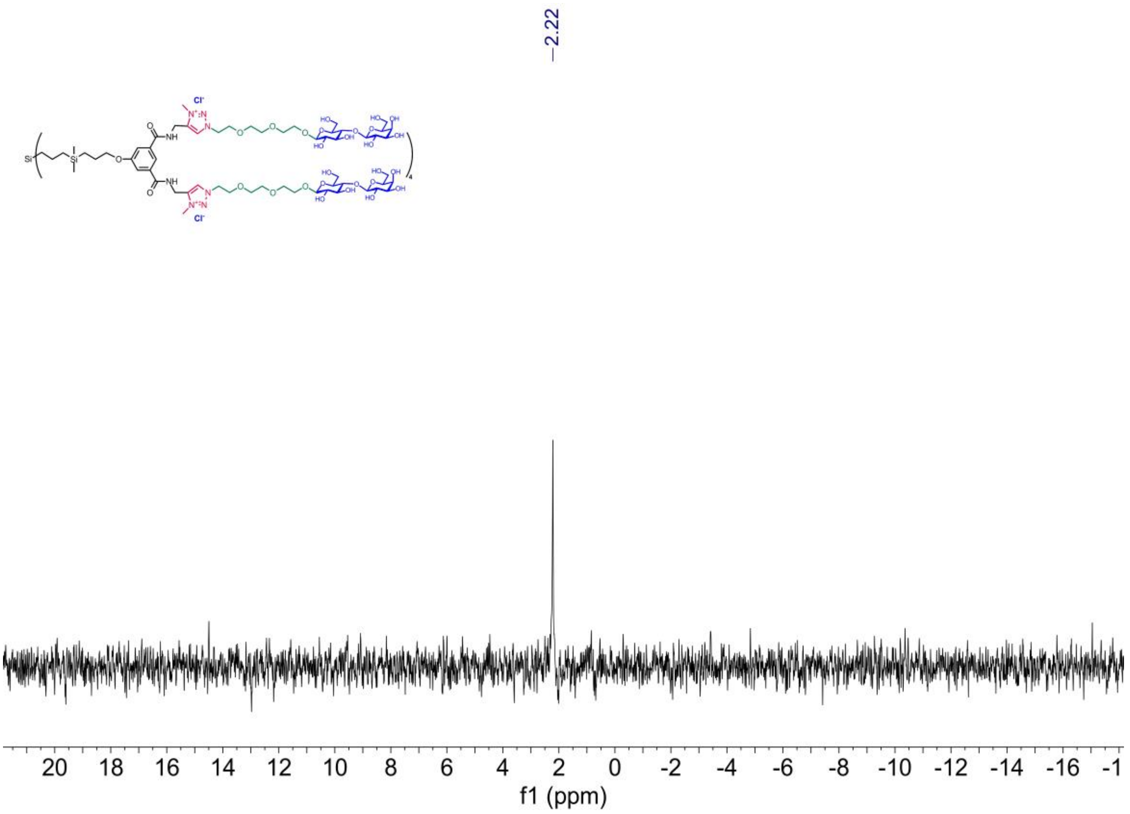

Figure S7: ^{29}Si {^{1}H} NMR (79 MHz, DMSO-d_6) **1c**

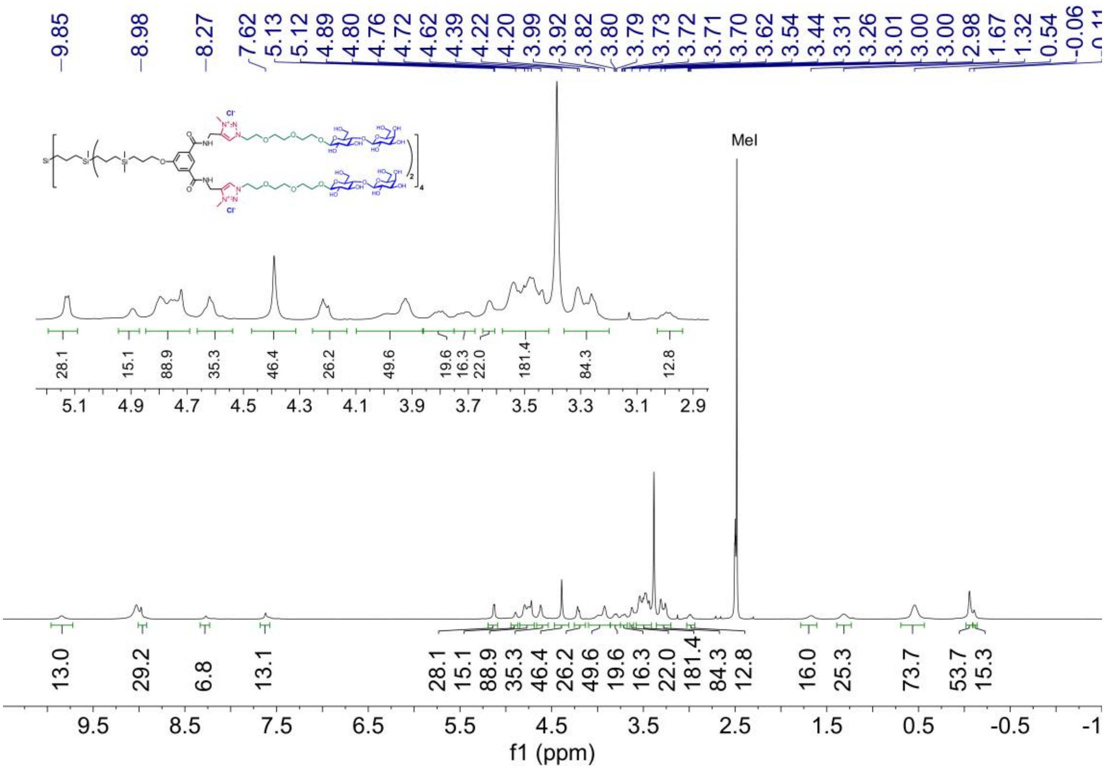

Figure S8: ^{1}H NMR (101 MHz, DMSO-d_6) **2c**

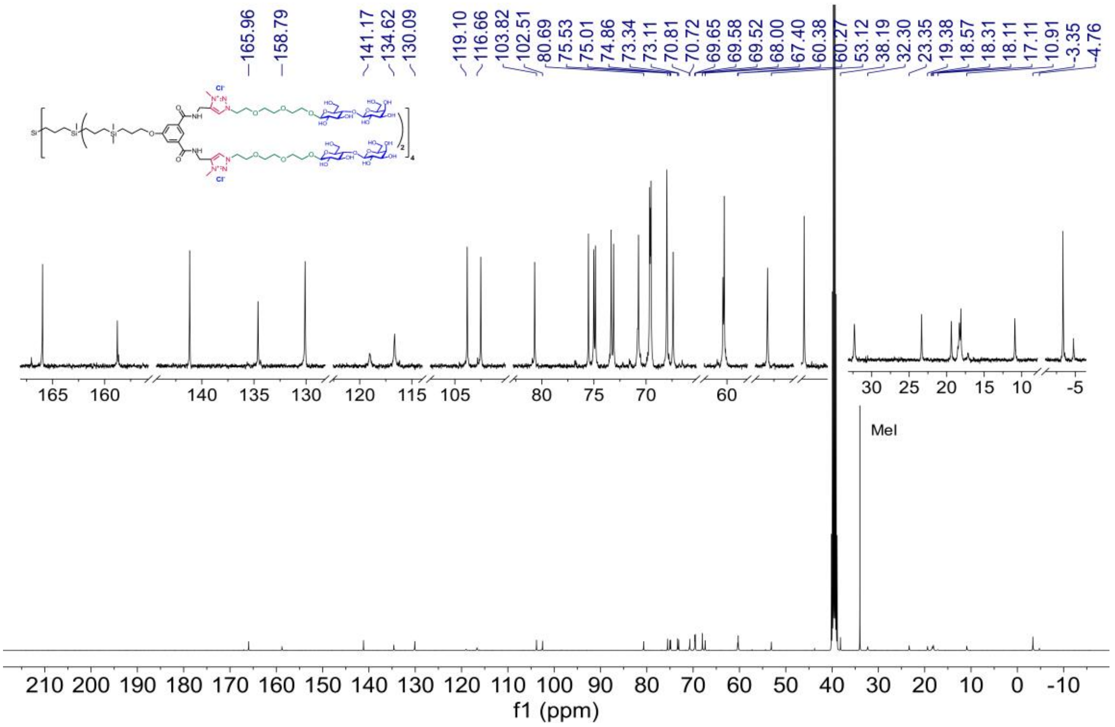

Figure S9: ^{13}C {^{1}H} NMR (101 MHz, DMSO-d_6) **2c**

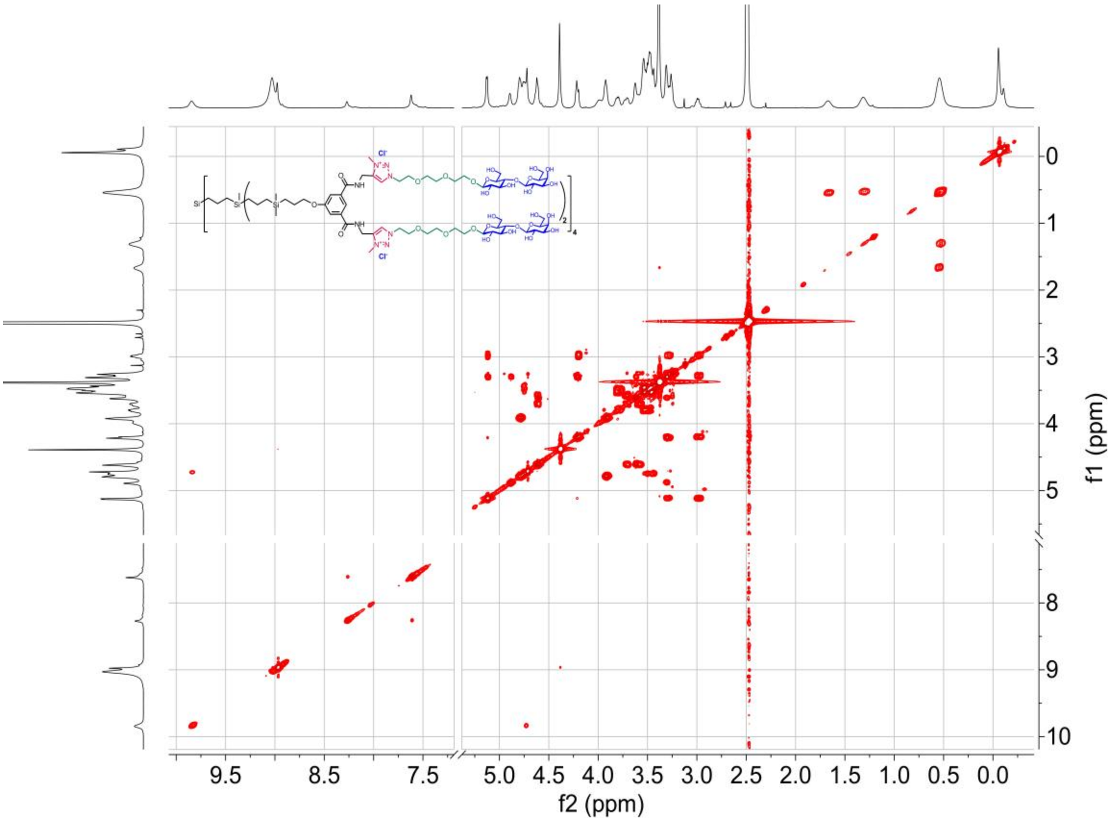

Figure S10: ^{1}H-^{1}H COSY NMR (DMSO-d_6) **2c**

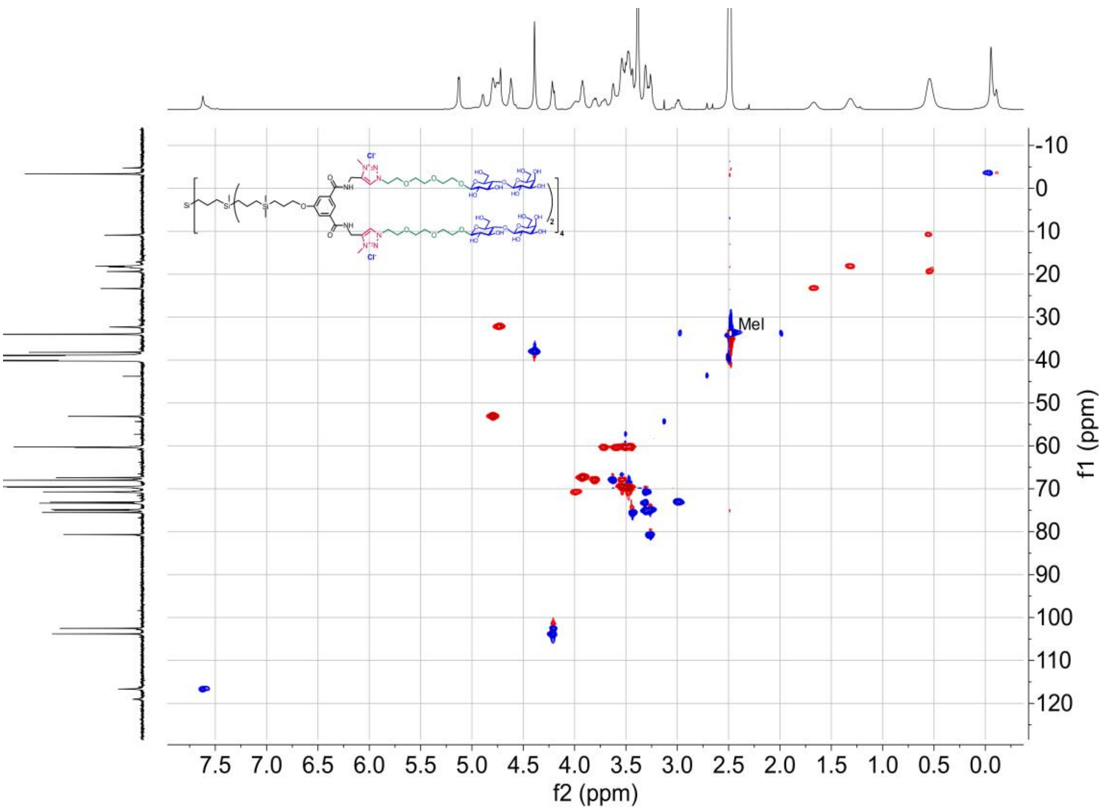

Figure S11:^{1}H-^{13}C HSQC NMR (DMSO-d_6) **2c**

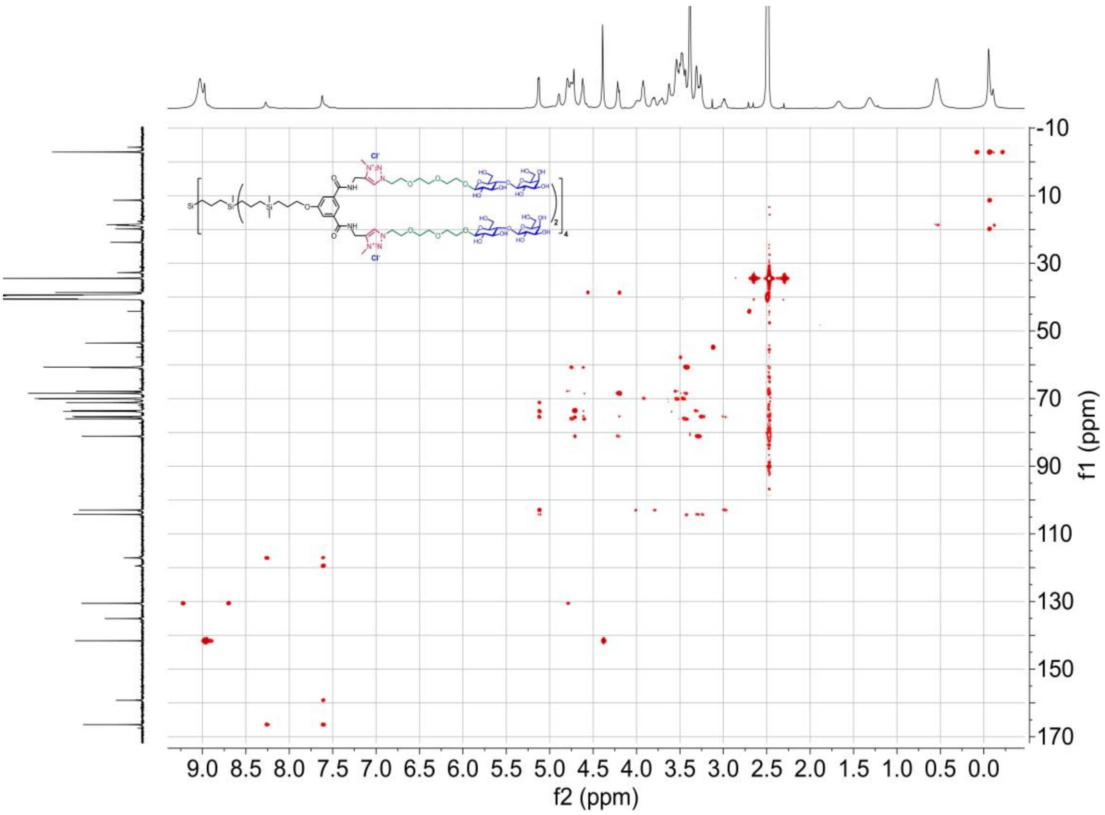

Figure S12:^{1}H-^{13}C HMBC NMR (DMSO-d_6) **2c**

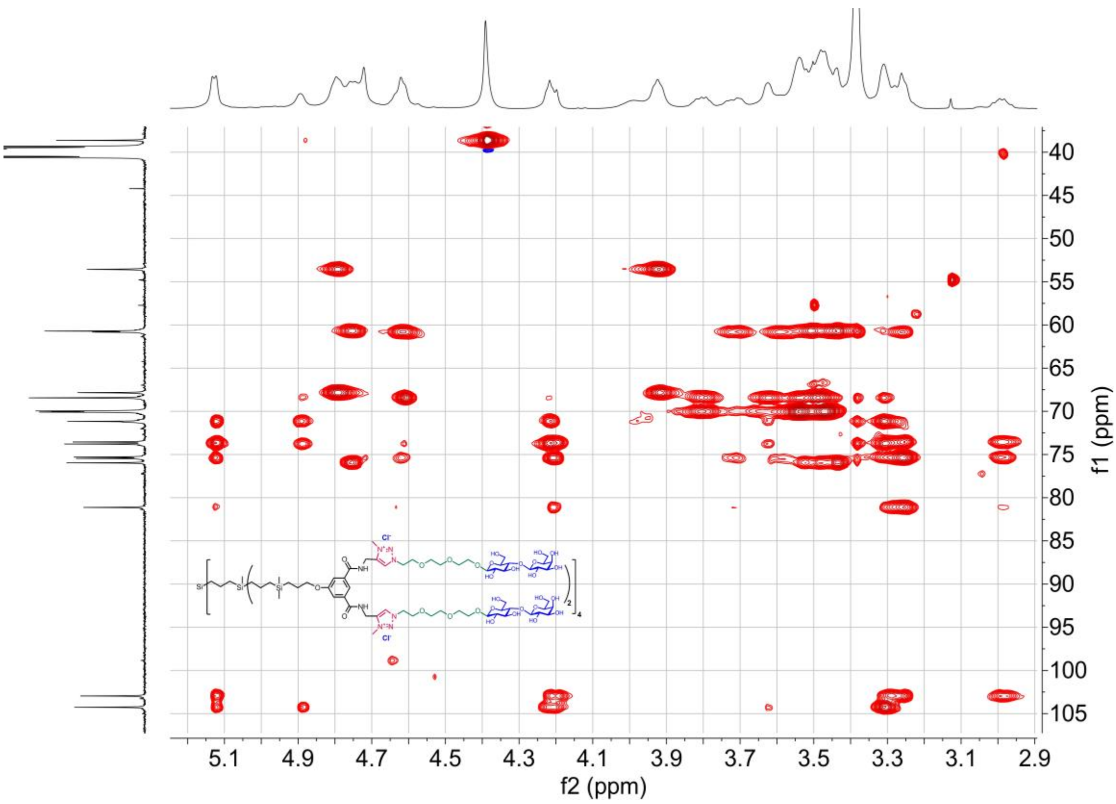

Figure S13:^{1}H-^{13}C HSQC TOCSY NMR (DMSO-d_6) **2c**

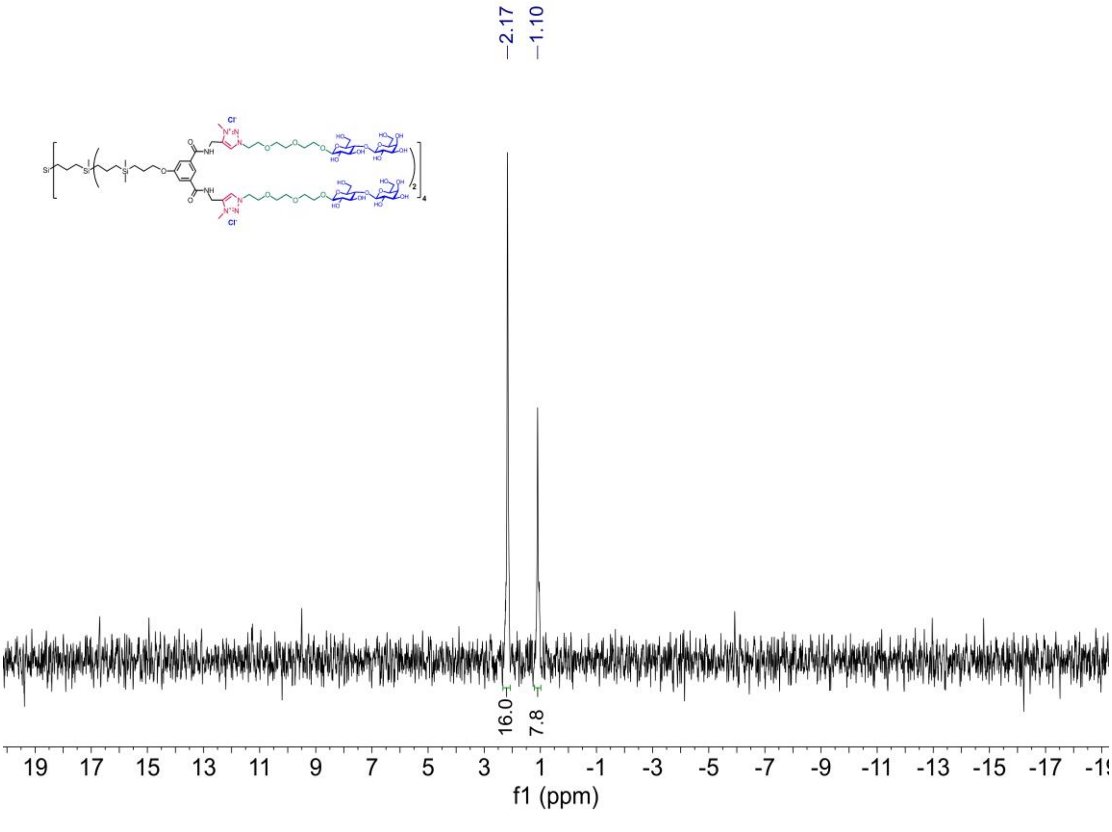

Figure S14:^{29}Si {^{1}H} NMR (79 MHz, DMSO-d_6) **2c**

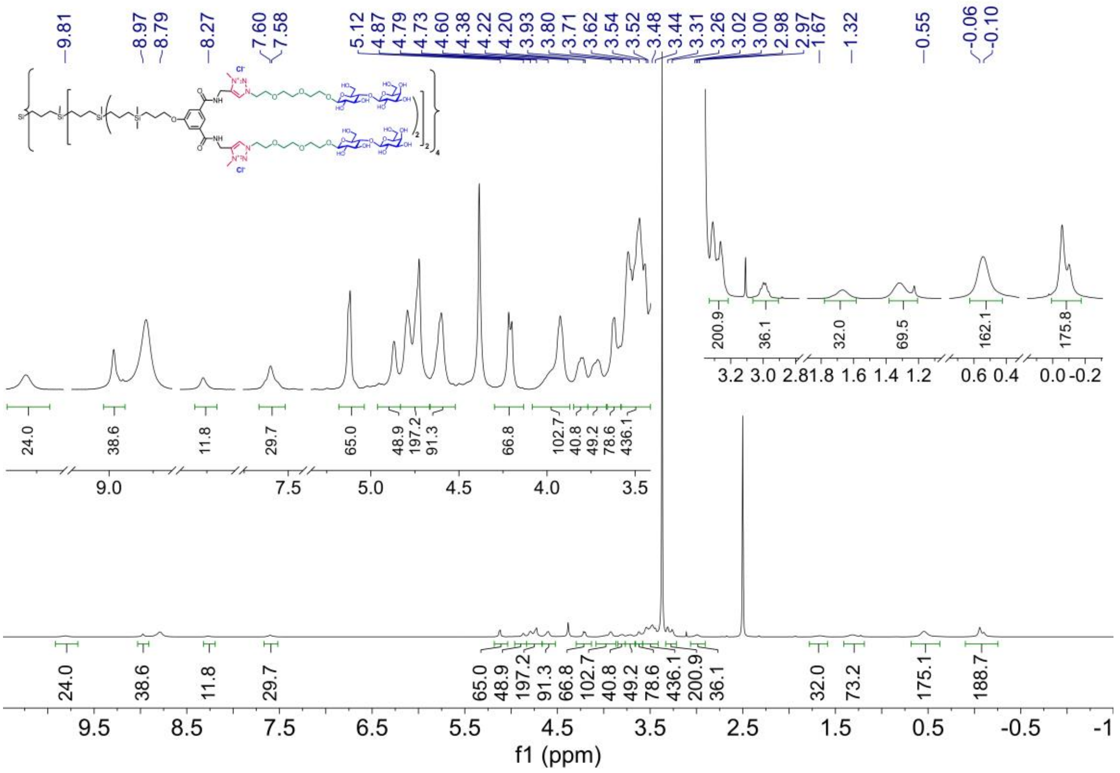

Figure S15:^{1}H NMR (101 MHz, DMSO-d_6) **3c**

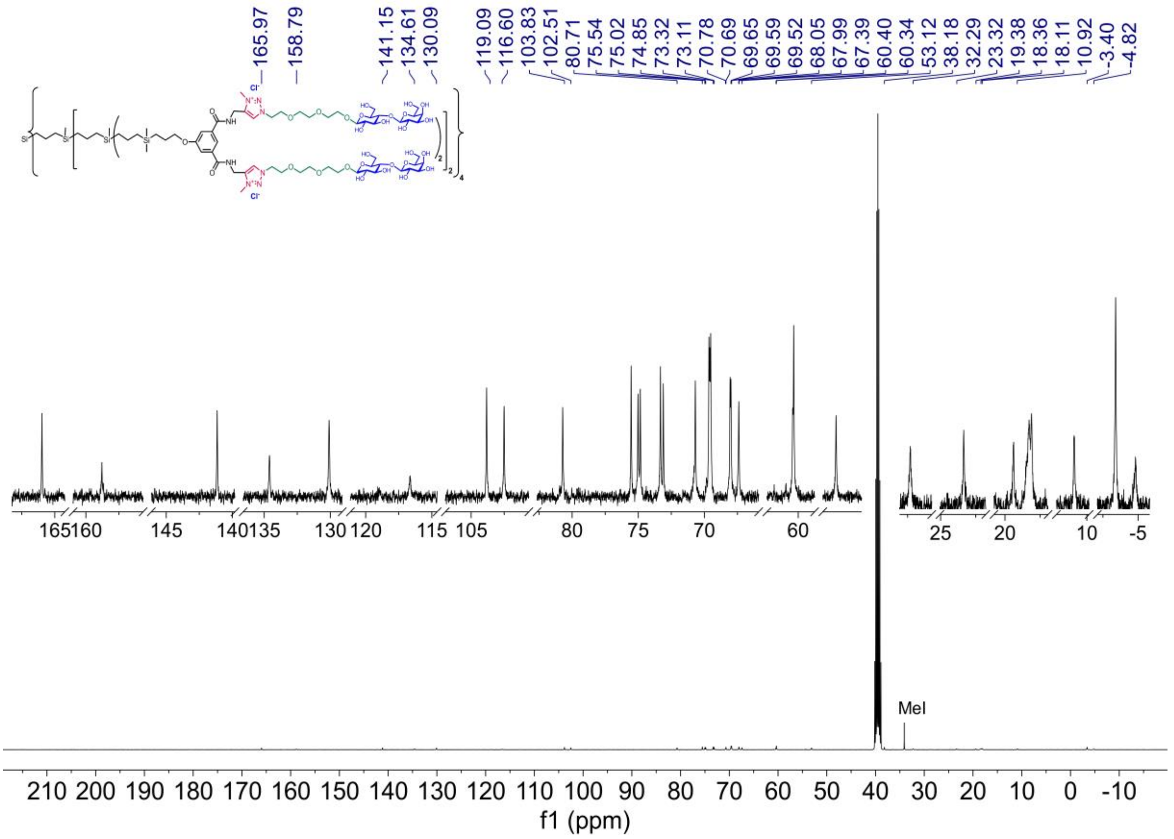

Figure S16:^{13}C {^{1}H} NMR (101 MHz, DMSO-d_6) **3c**

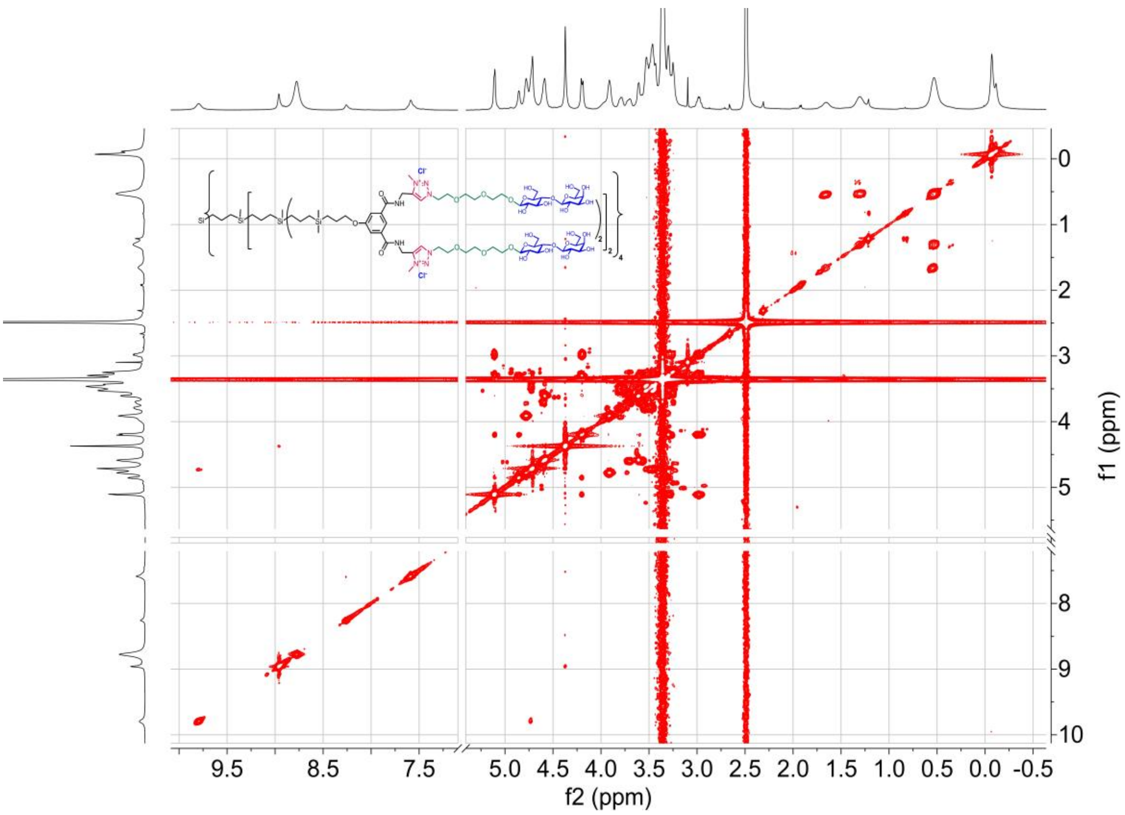

Figure S17:^{1}H-^{1}H COSY NMR (DMSO-d_6) **3c**

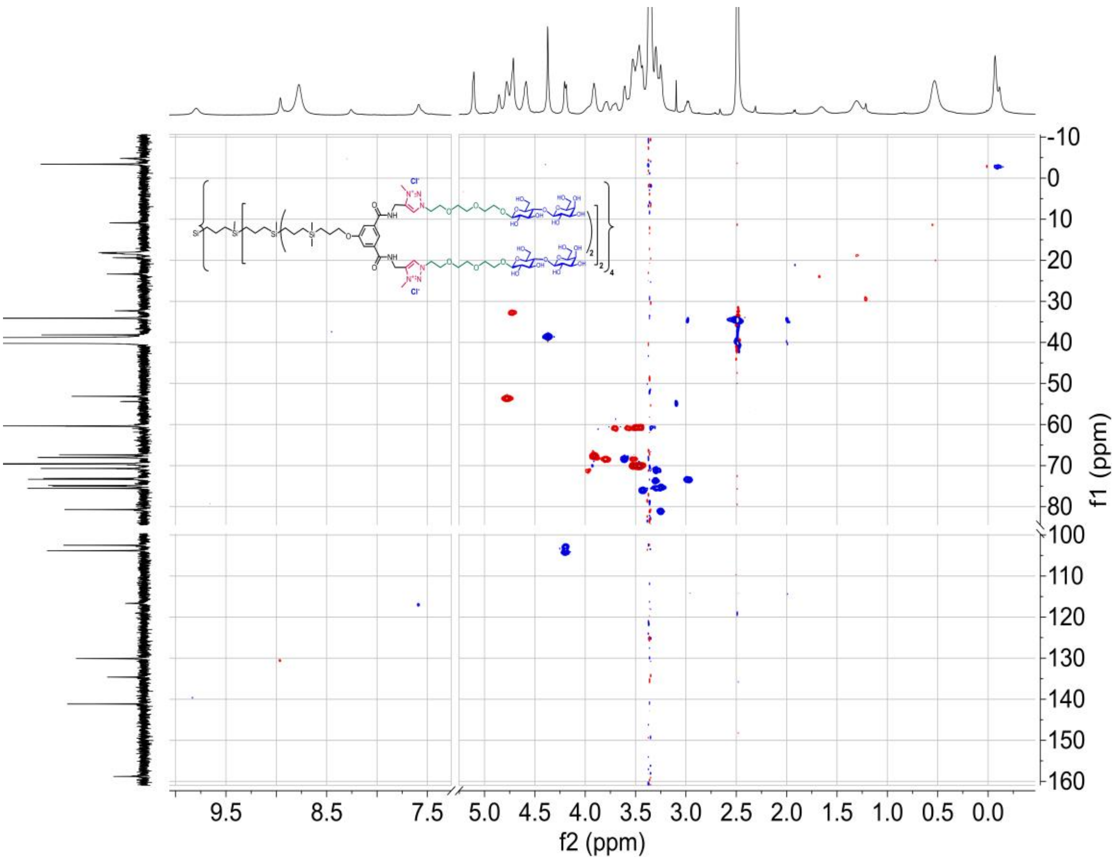

Figure S18: ^{1}H-^{13}C HSQC NMR (DMSO-d_6) **3c**

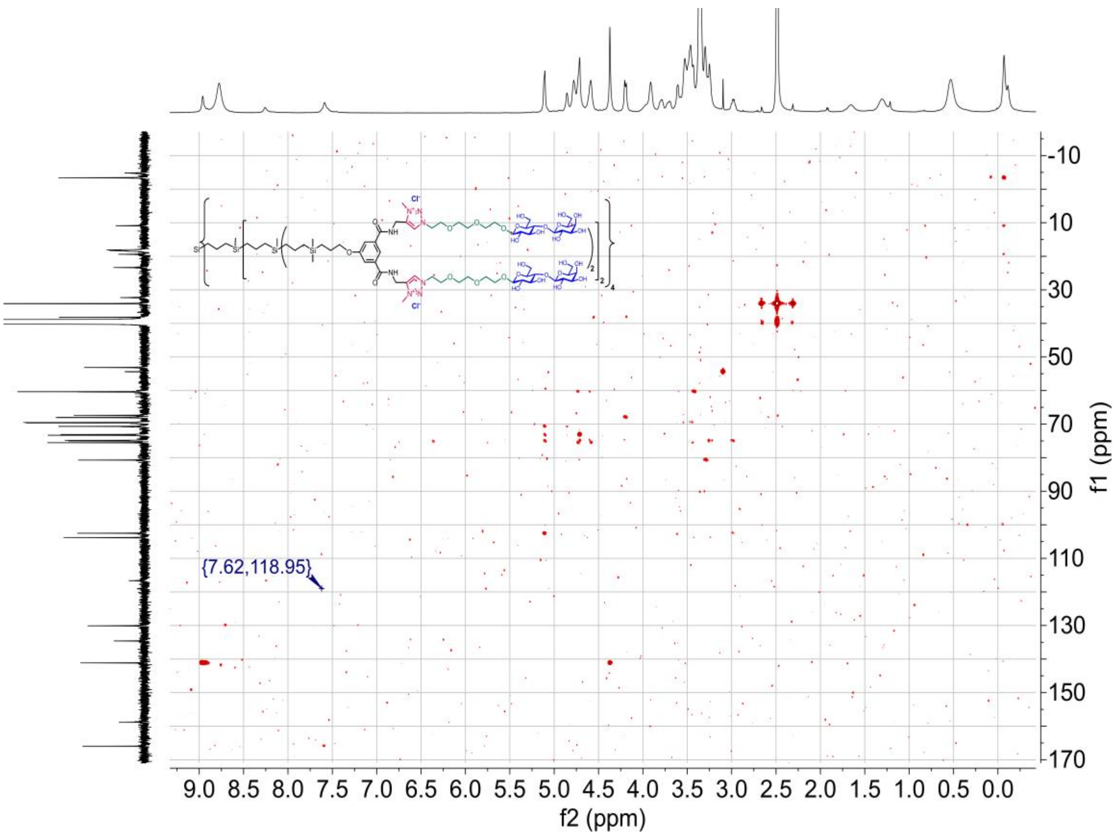

Figure S19: ^{1}H-^{13}C HMBC NMR (DMSO-d_6) **3c**

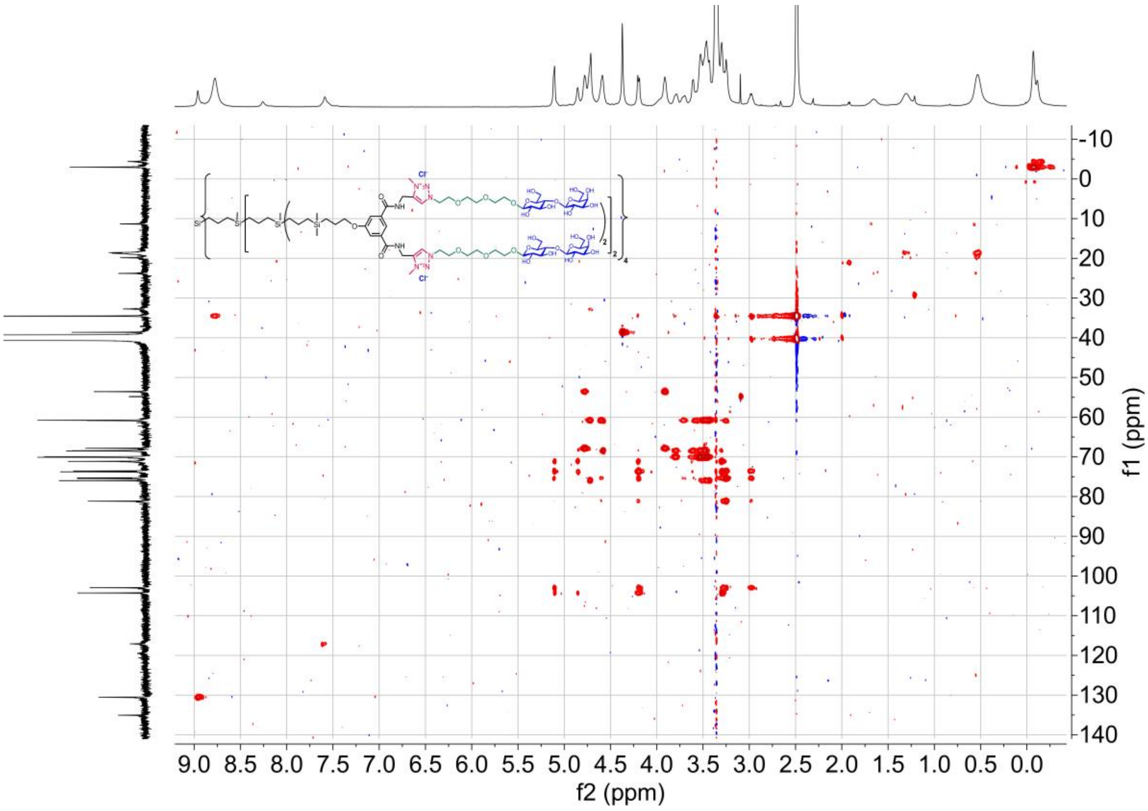

Figure S20: ^{1}H-^{13}C HSQC TOCSY NMR (DMSO-d_6) **3c**

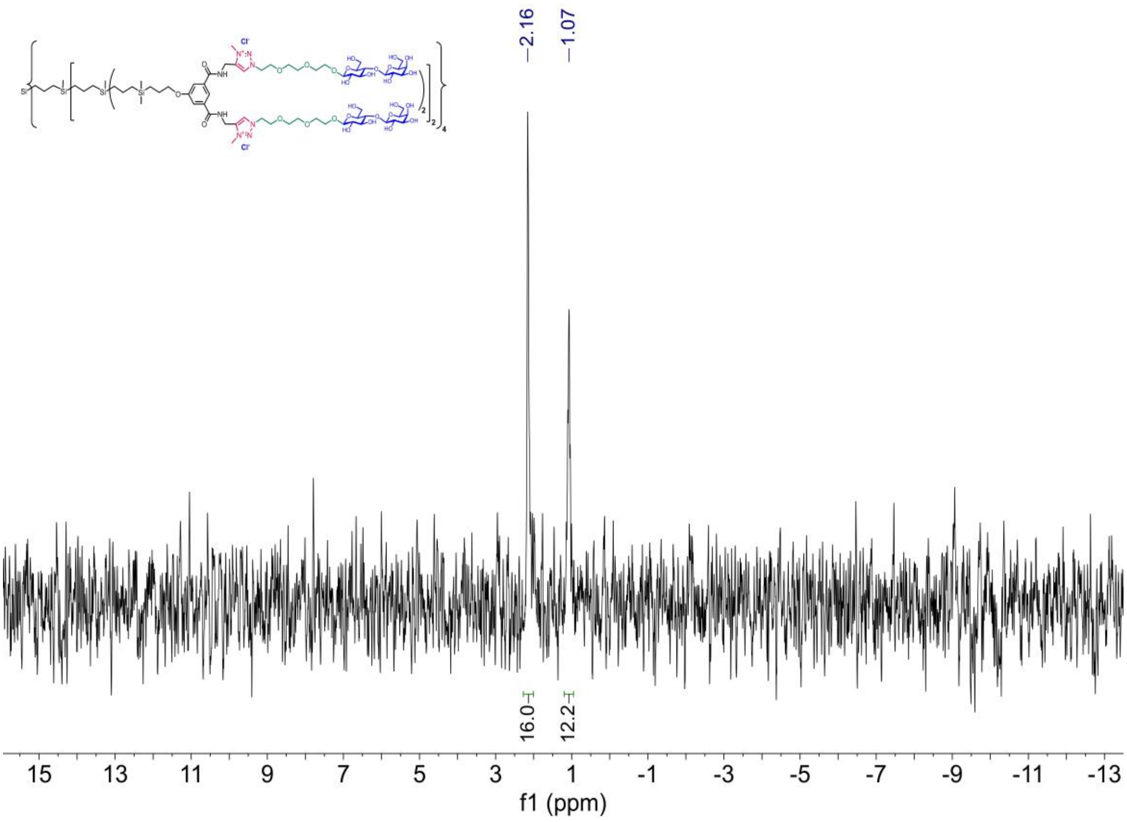

Figure S21: ^{29}Si {^{1}H} NMR (79 MHz, DMSO-d_6) **3c**

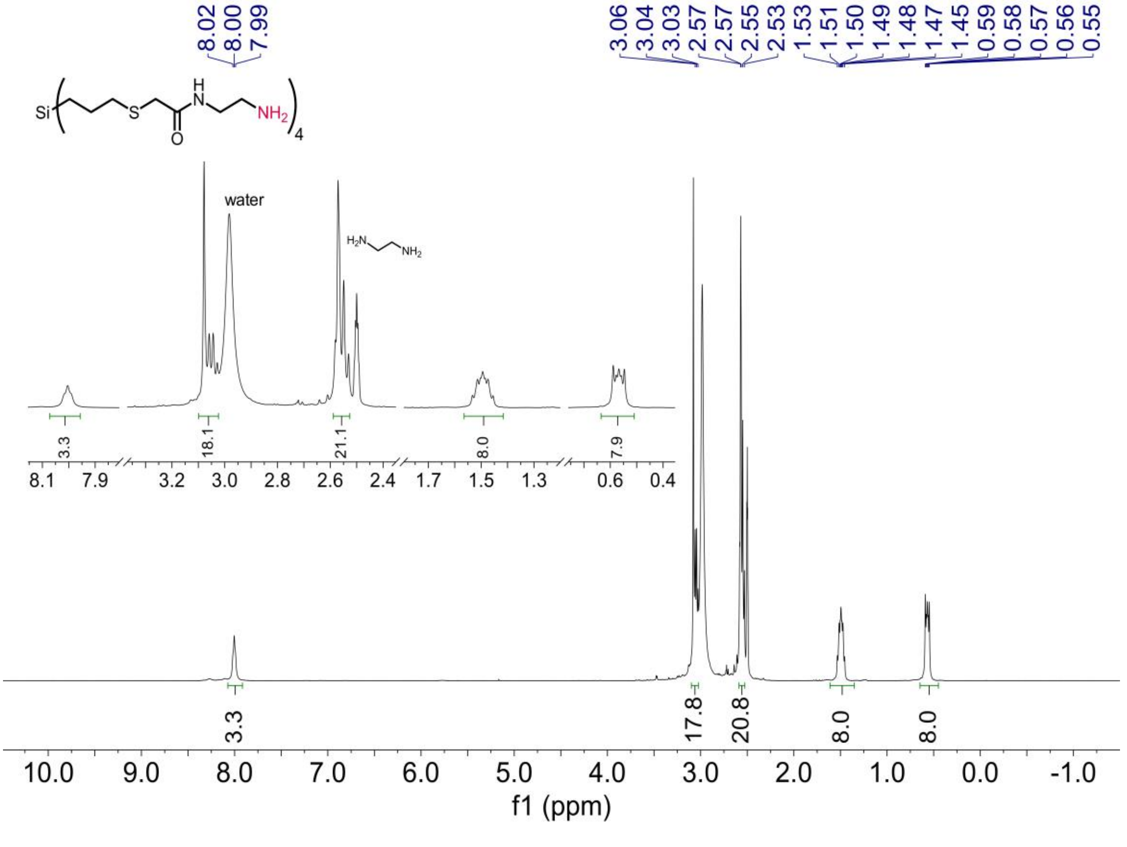

Figure S22: ^{1}H NMR (101 MHz, DMSO-d_6) **7**

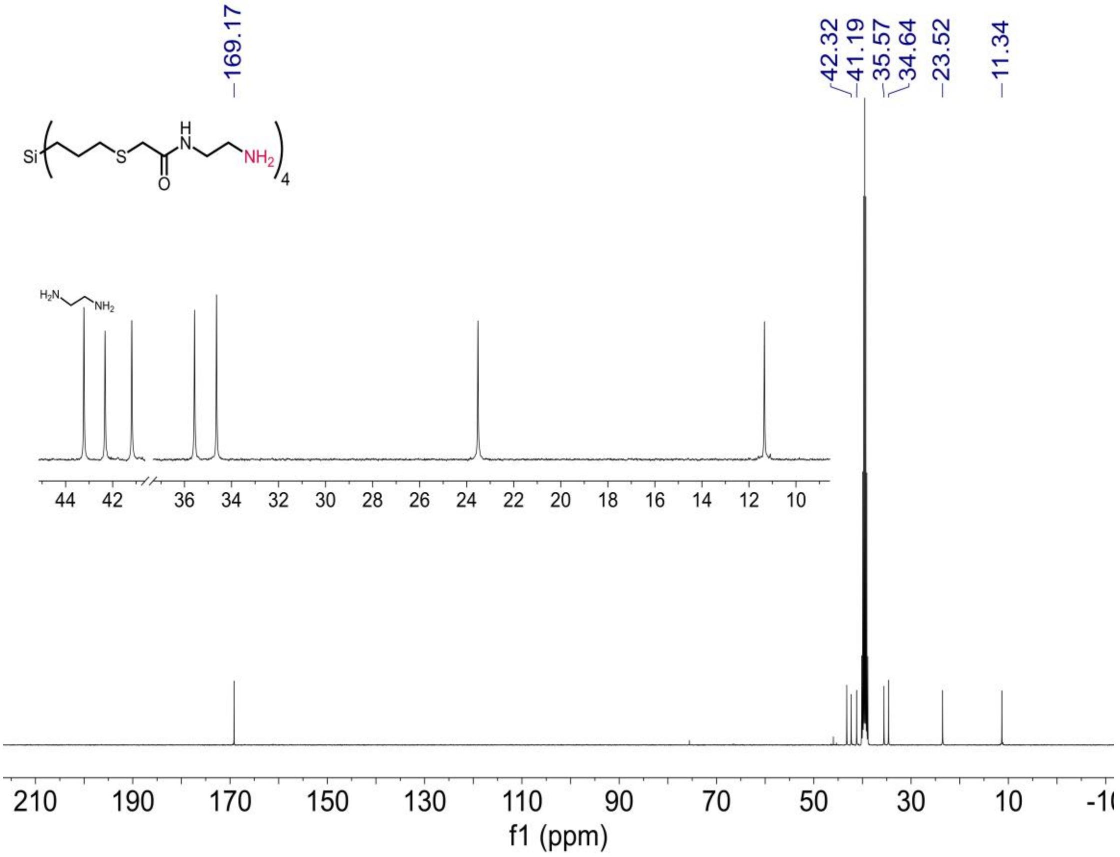

Figure S23: ^{13}C {^{1}H} NMR (101 MHz, DMSO-d_6) **7**

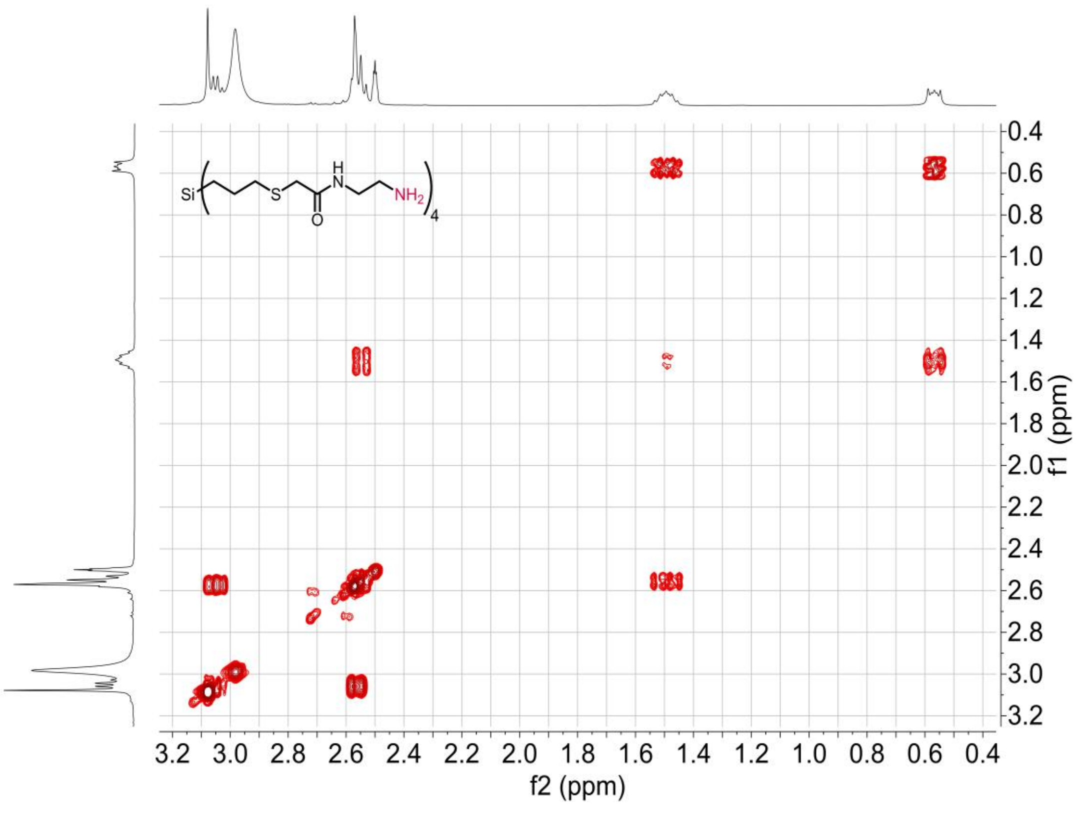

Figure S24: ^{1}H-^{1}H COSY NMR (DMSO-d_6) **7**

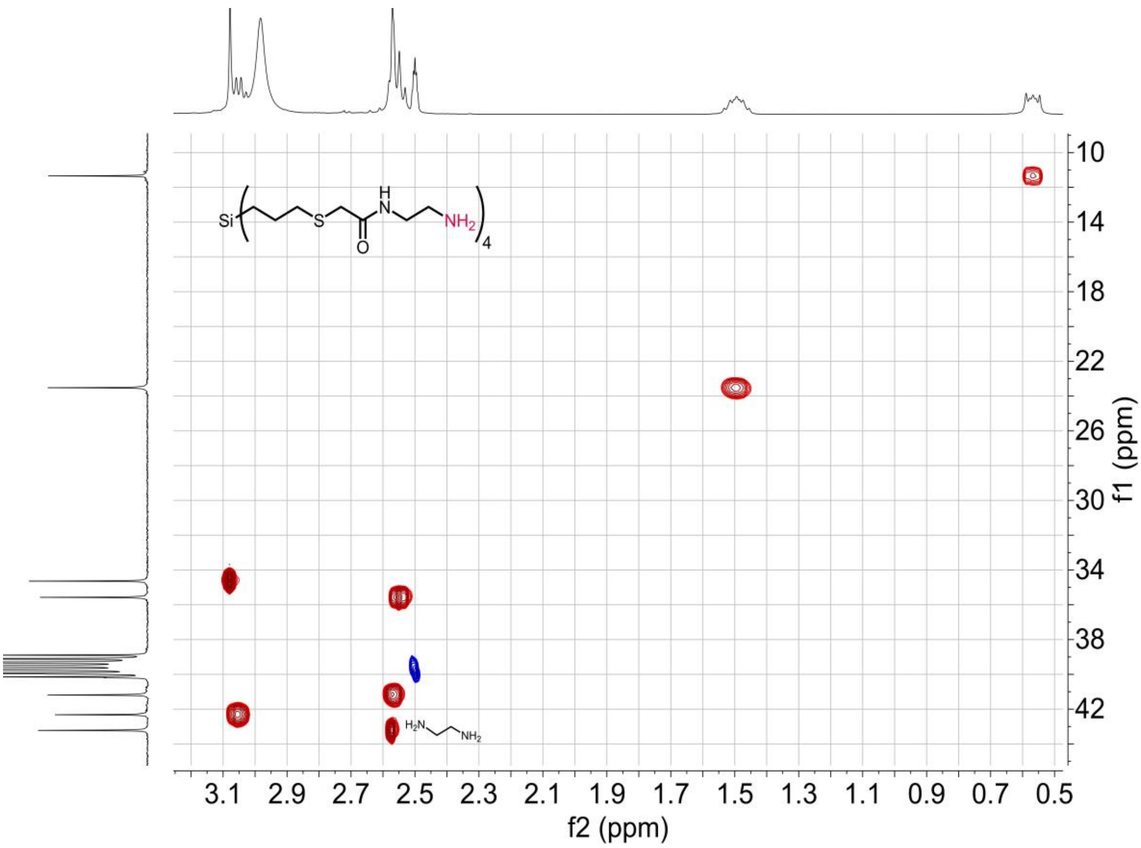

Figure S25: ^{1}H-^{13}C HSQC NMR (DMSO-d_6) **7**

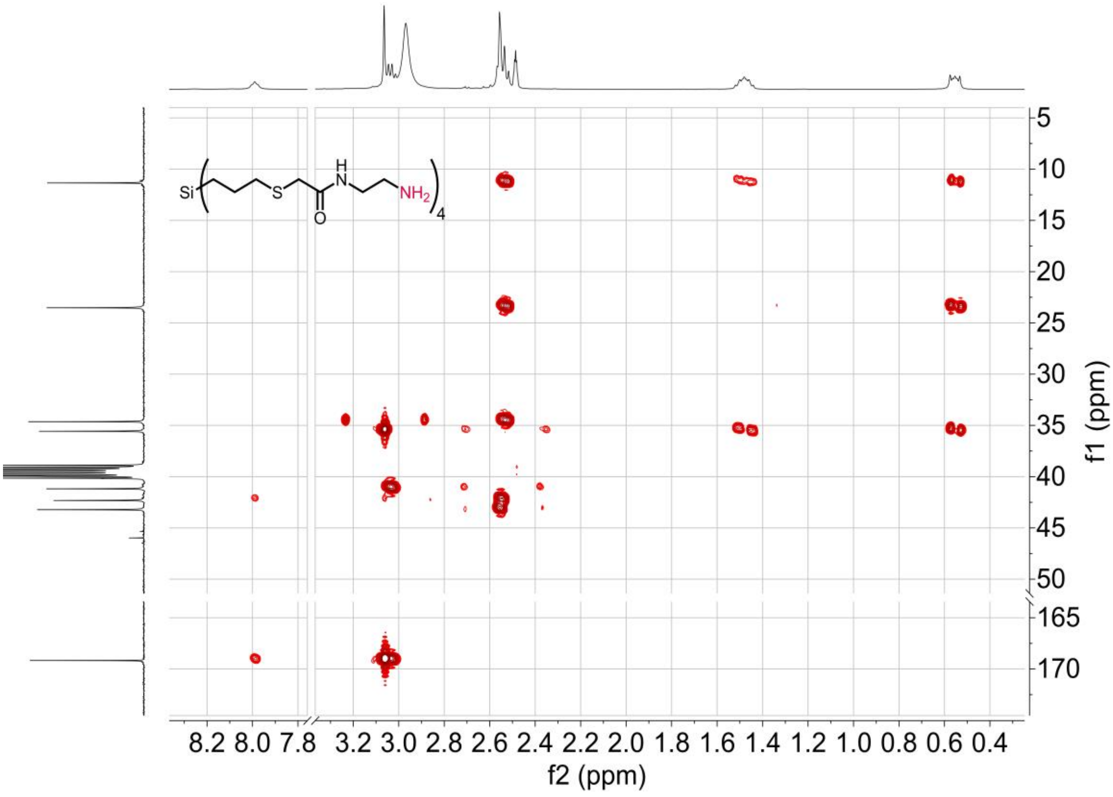

Figure S26: ^{1}H-^{13}C HMBC NMR (DMSO-d_6) **7**

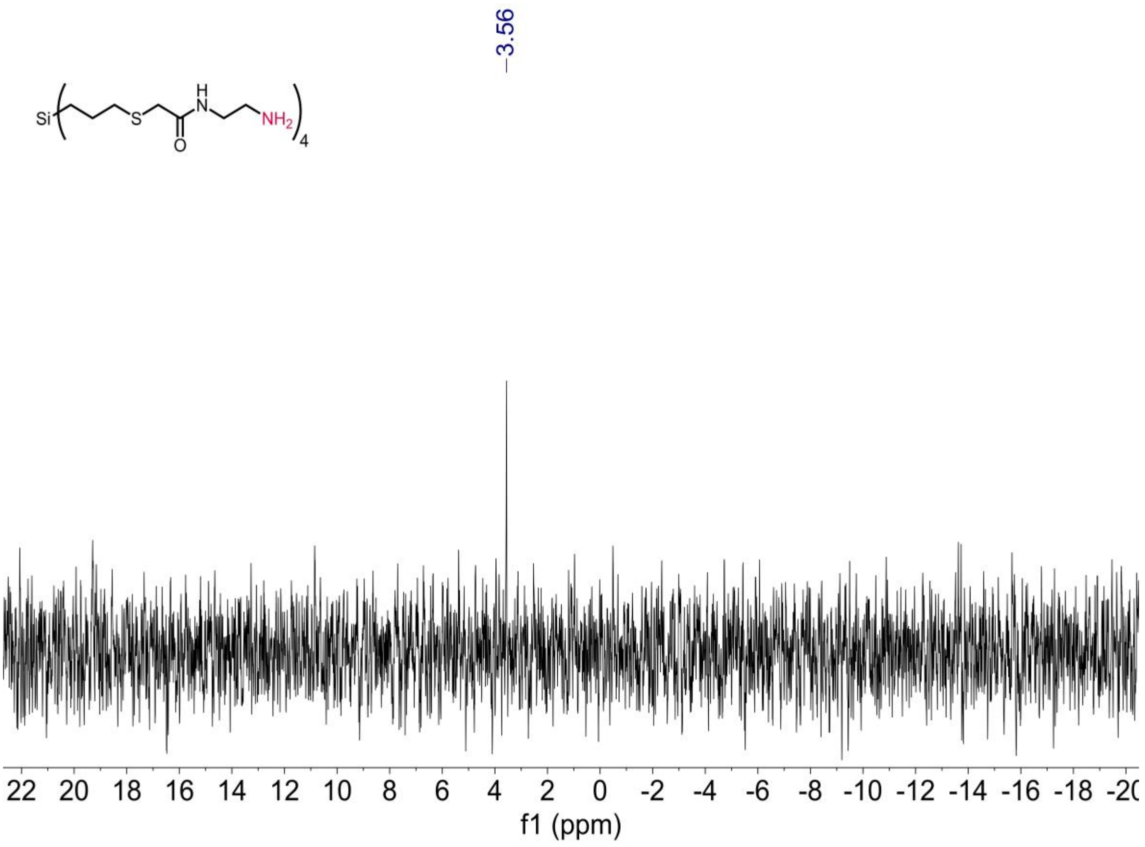

Figure S27: ^{29}Si {^{1}H} NMR (79 MHz, DMSO-d_6) **7**

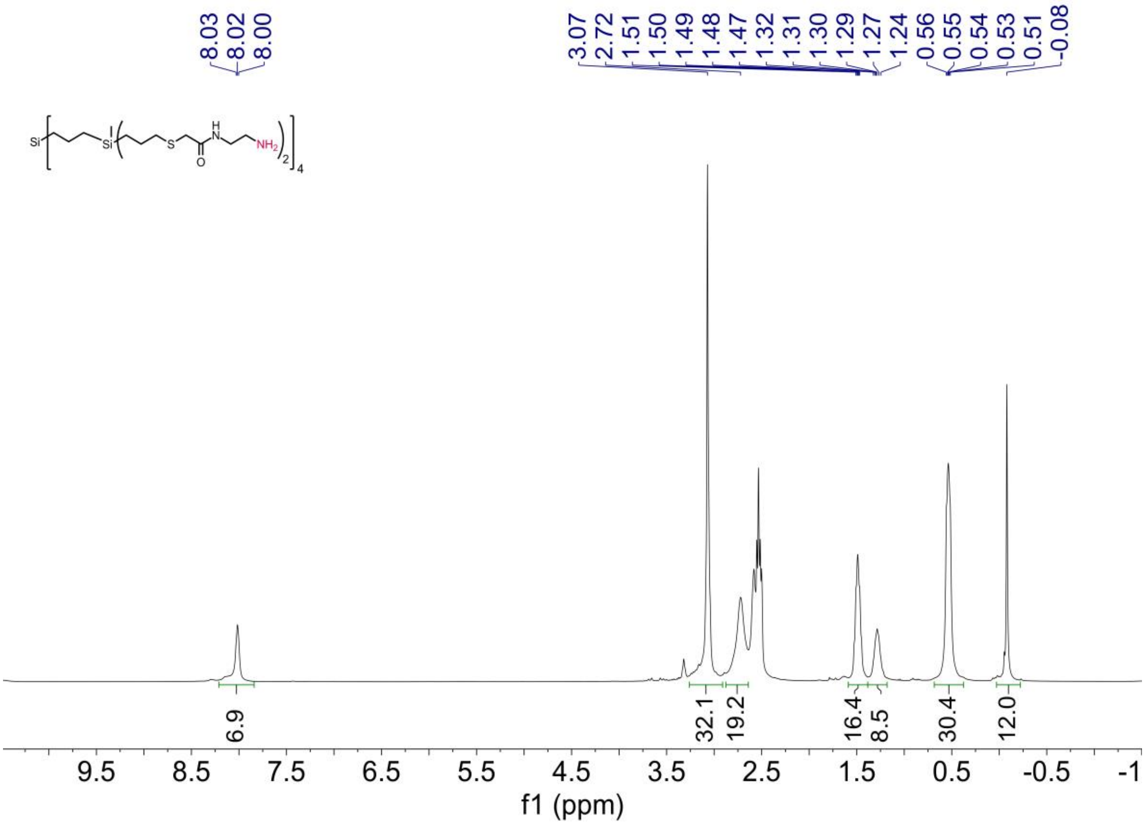

Figure S28: ^{1}H NMR (101 MHz, DMSO-d_6) **8**

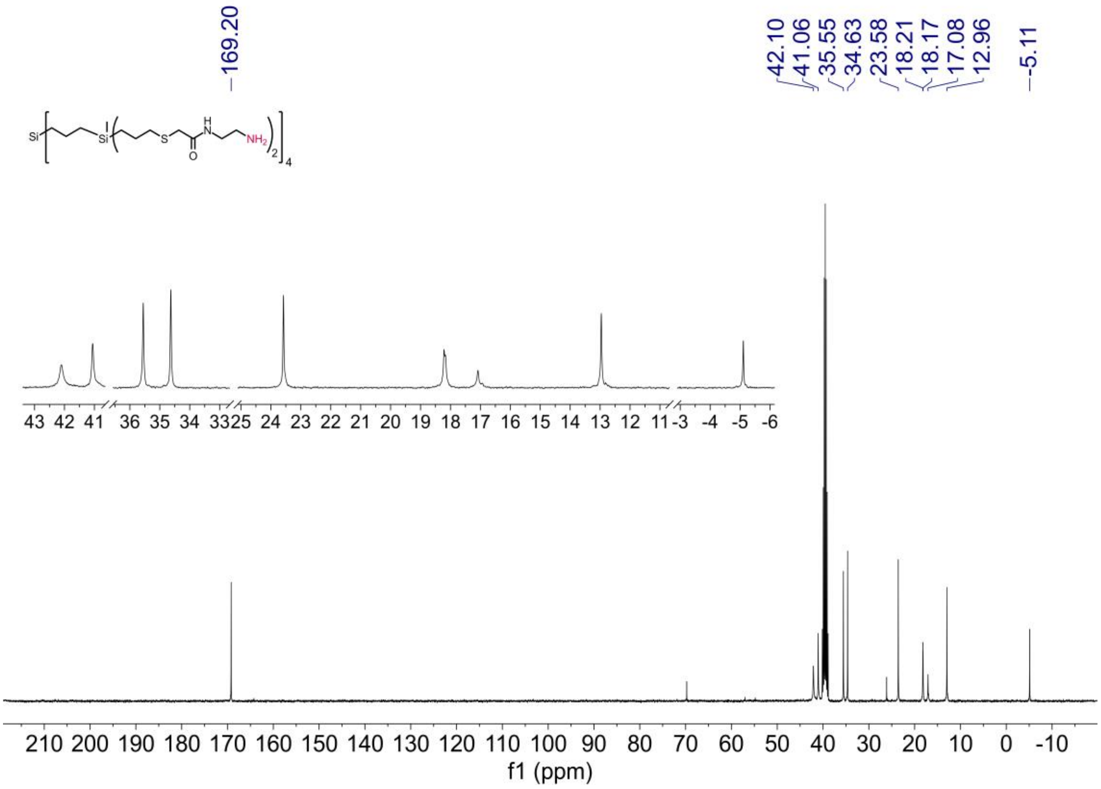

Figure S29: $^{13}C\ \{^{1}H\}$ NMR (101 MHz, DMSO-d_6) **8**

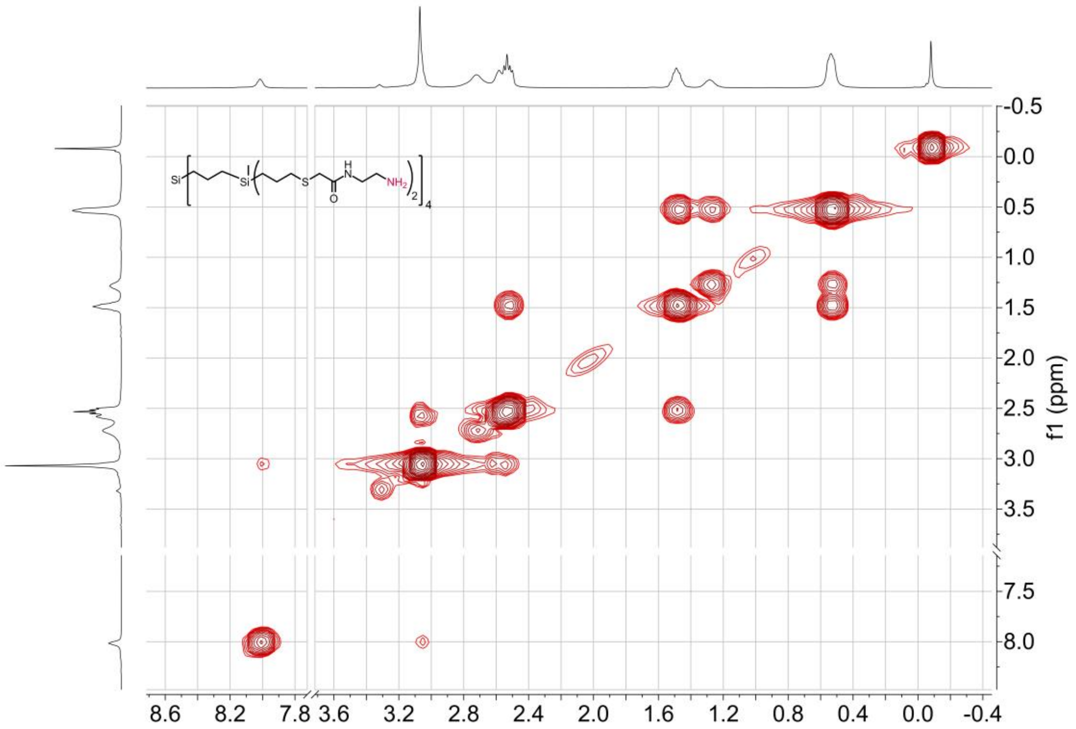

Figure S30: ^{1}H-^{1}H COSY NMR (DMSO-d_6) **8**

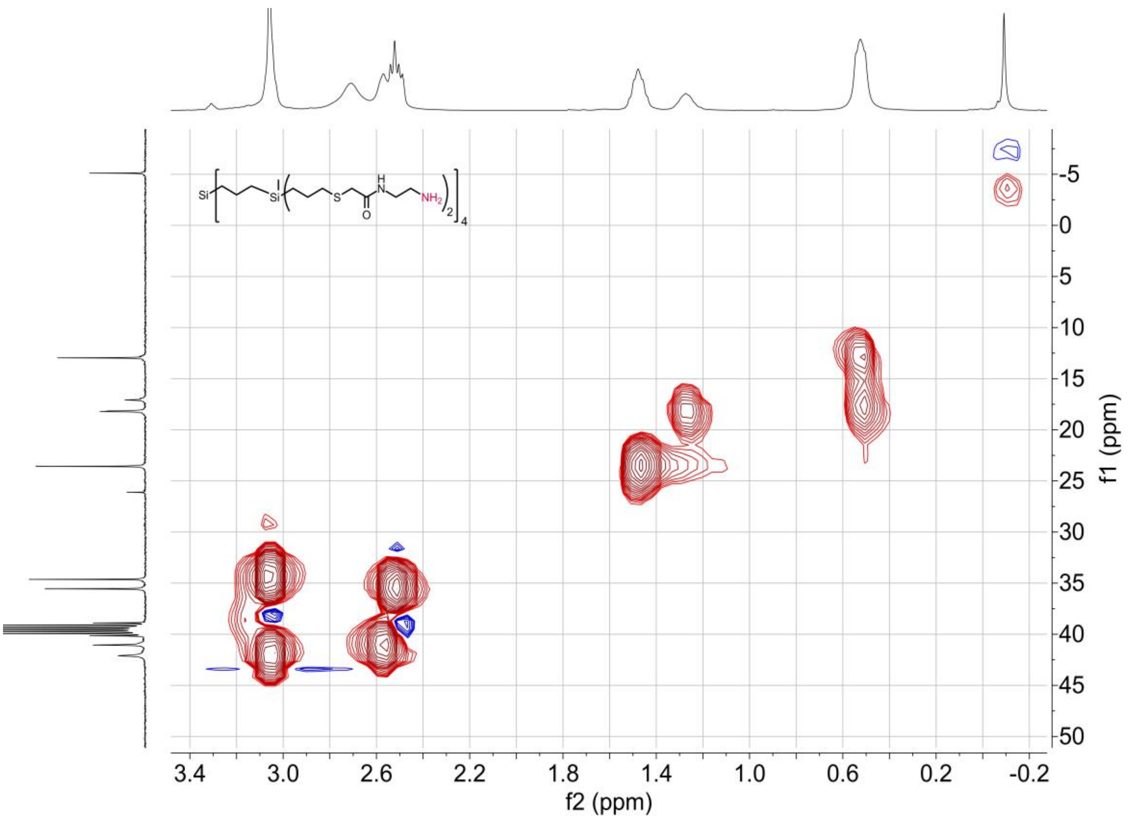

Figure S31: ^{1}H-^{13}C HSQC NMR (DMSO-d_6) **8**

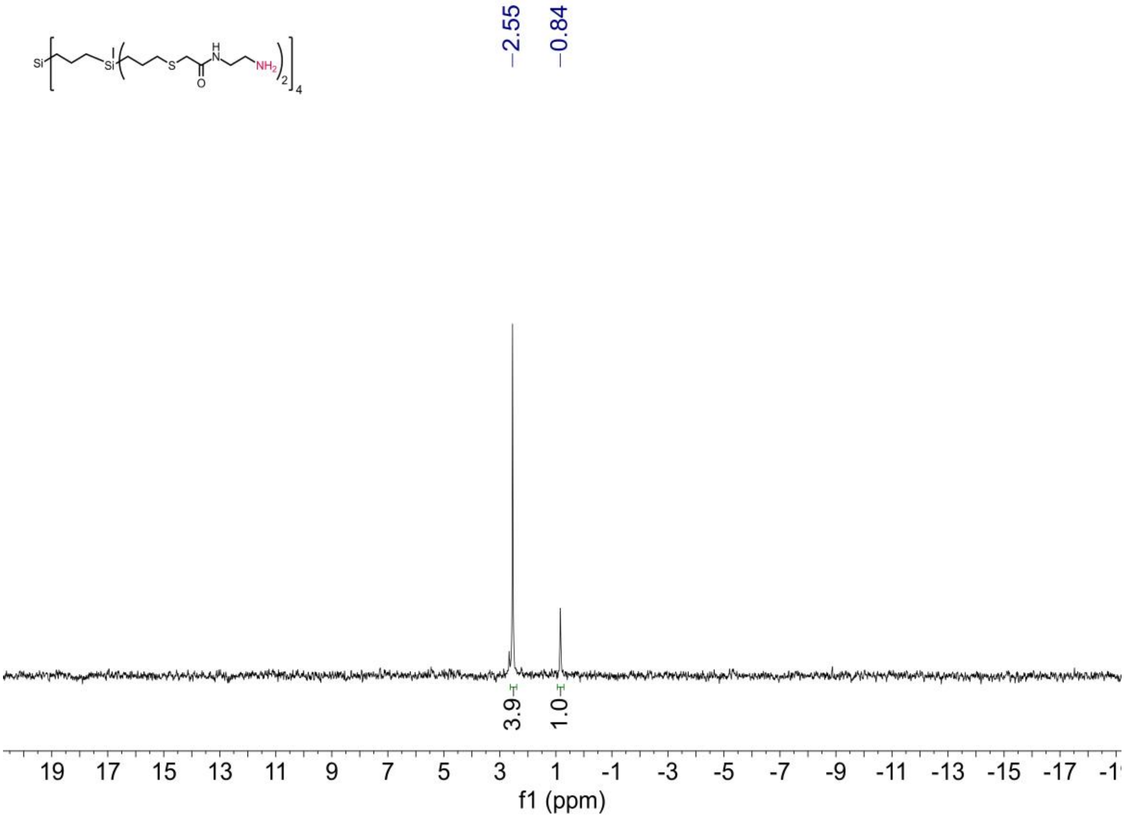

Figure S32: ^{29}Si {^{1}H} NMR (79 MHz, DMSO-d_6) **8**

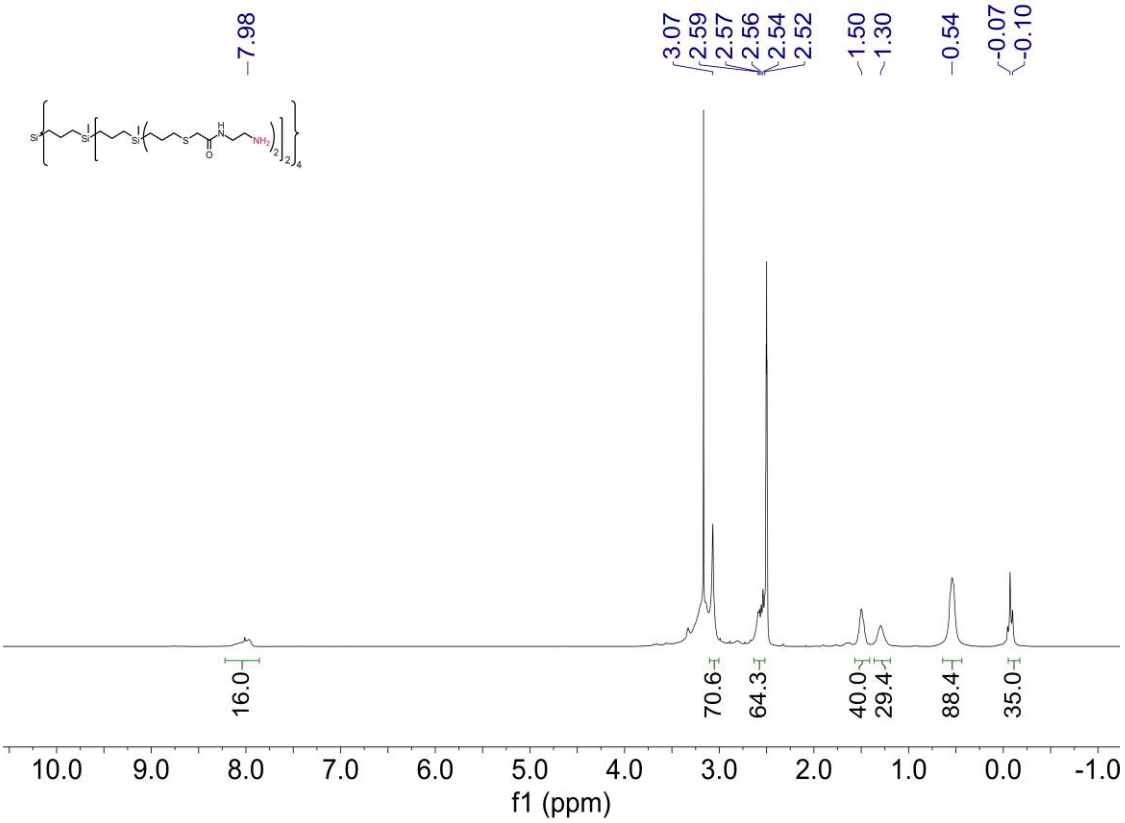

Figure S33: ^{1}H NMR (101 MHz, DMSO-d_6) **9**

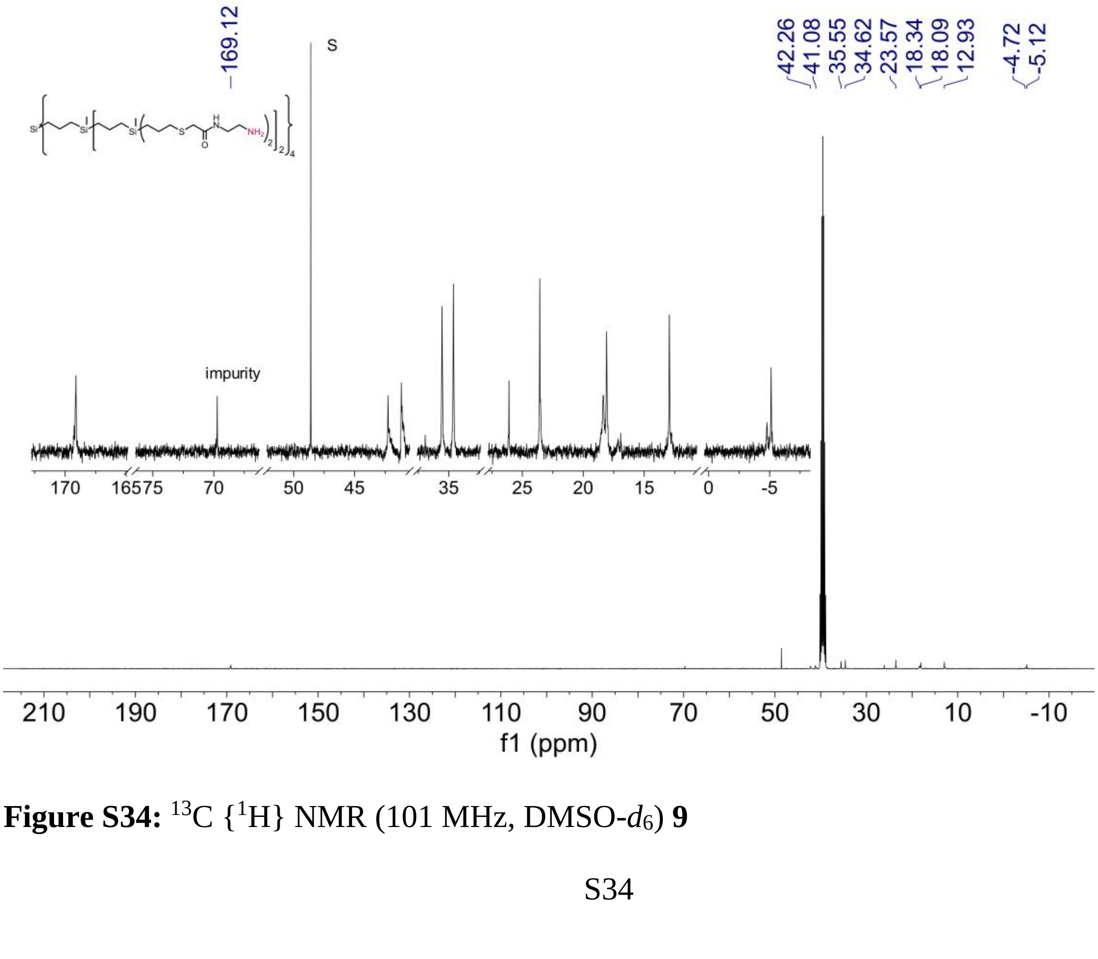

Figure S34: ^{13}C {^{1}H} NMR (101 MHz, DMSO-d_6) **9**

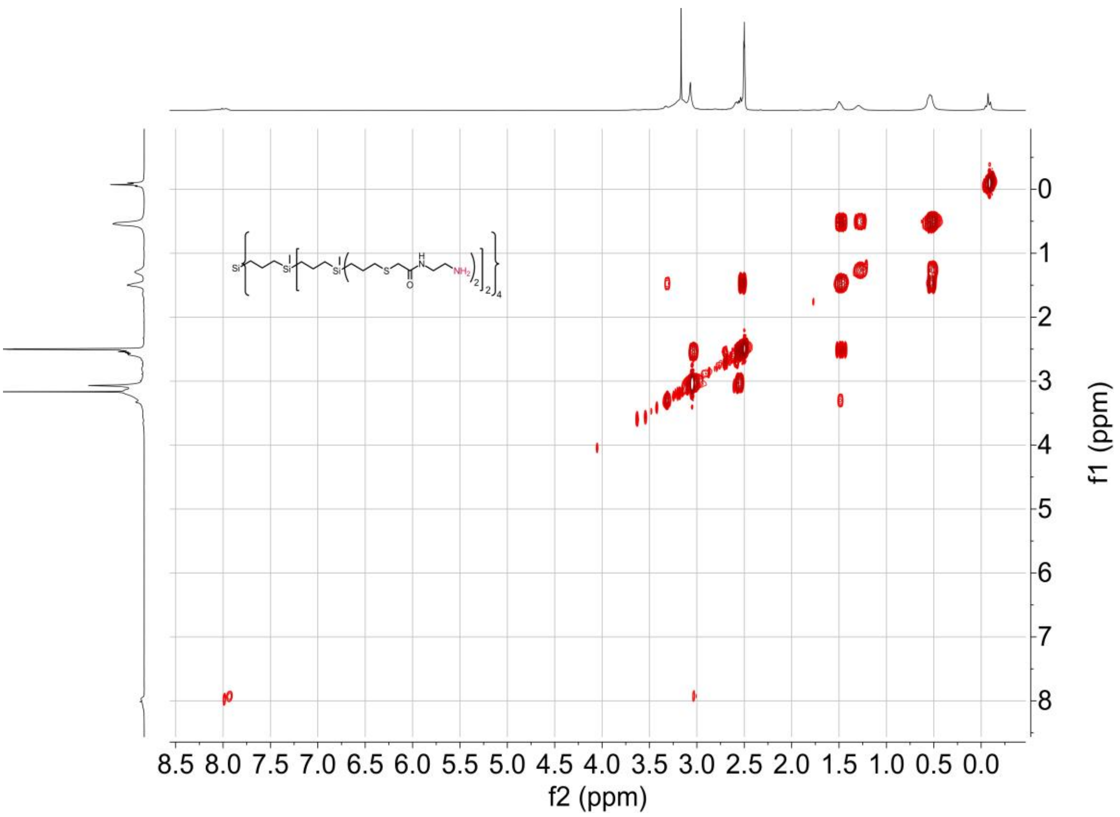

Figure S35: ^{1}H-^{1}H COSY NMR (DMSO-d_6) **9**

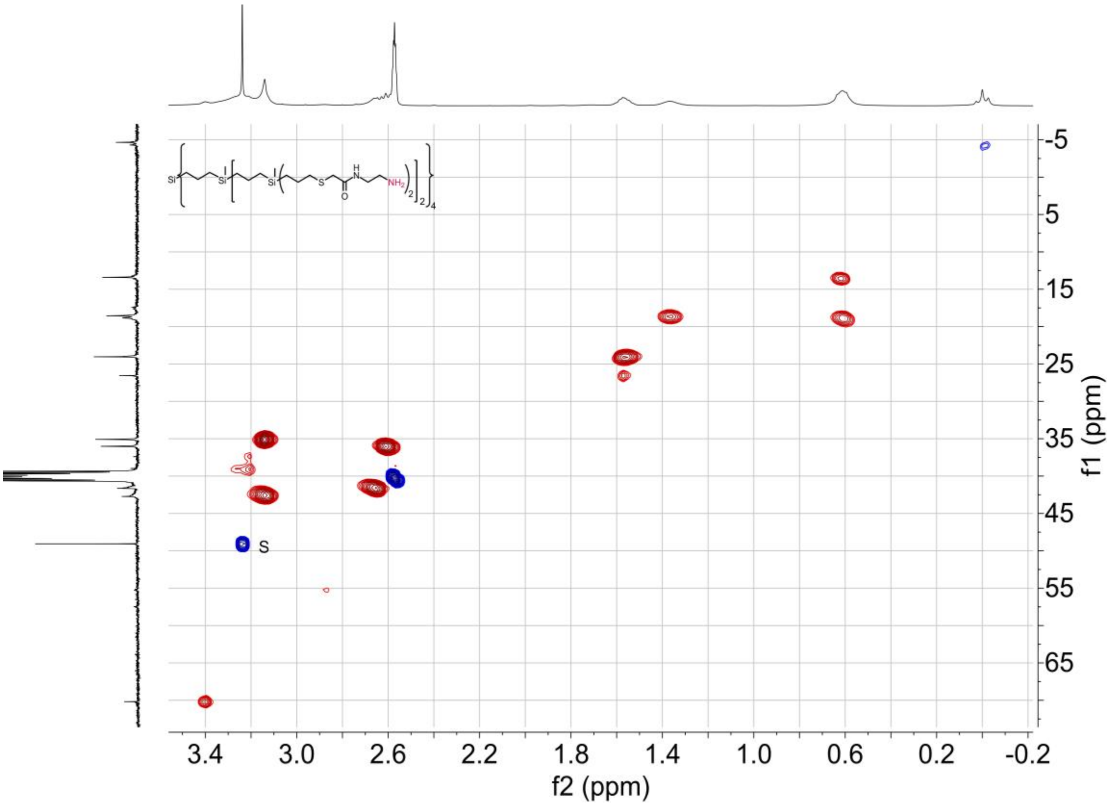

Figure S36: ^{1}H-^{13}C HSQC NMR (DMSO-d_6) **9**

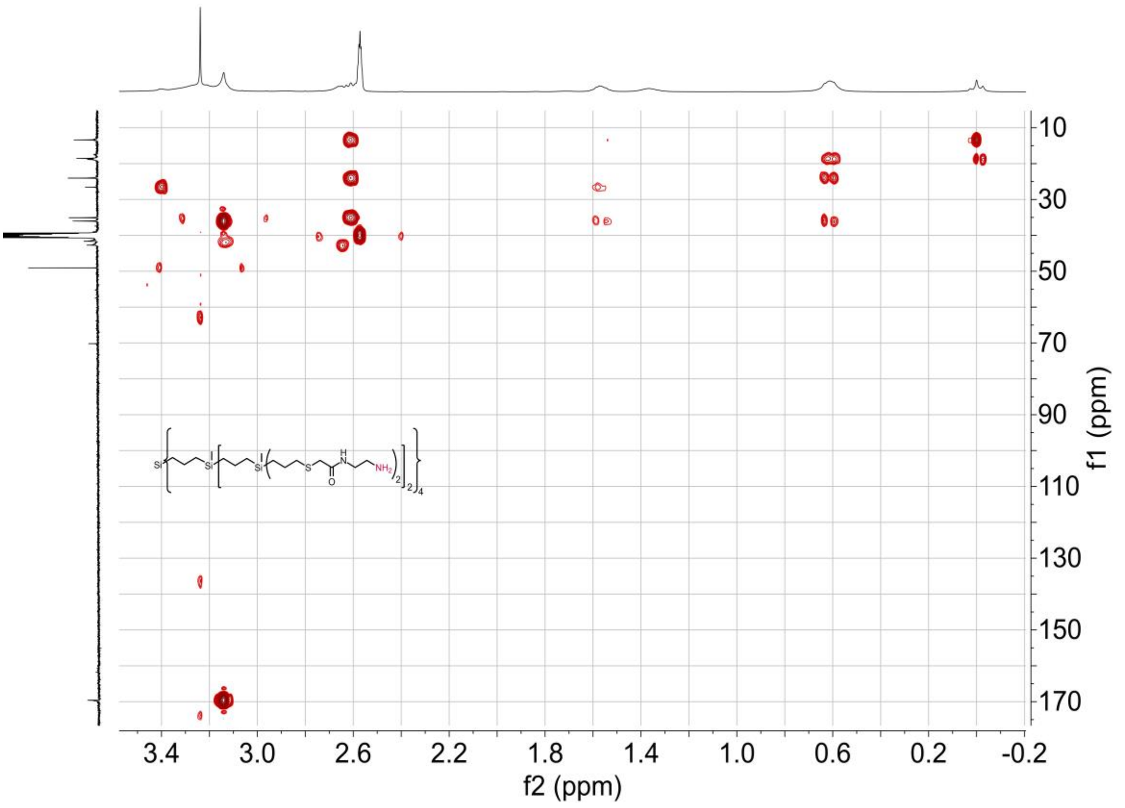

Figure S37: ^{1}H-^{13}C HMBC NMR (DMSO-d_6) **9**

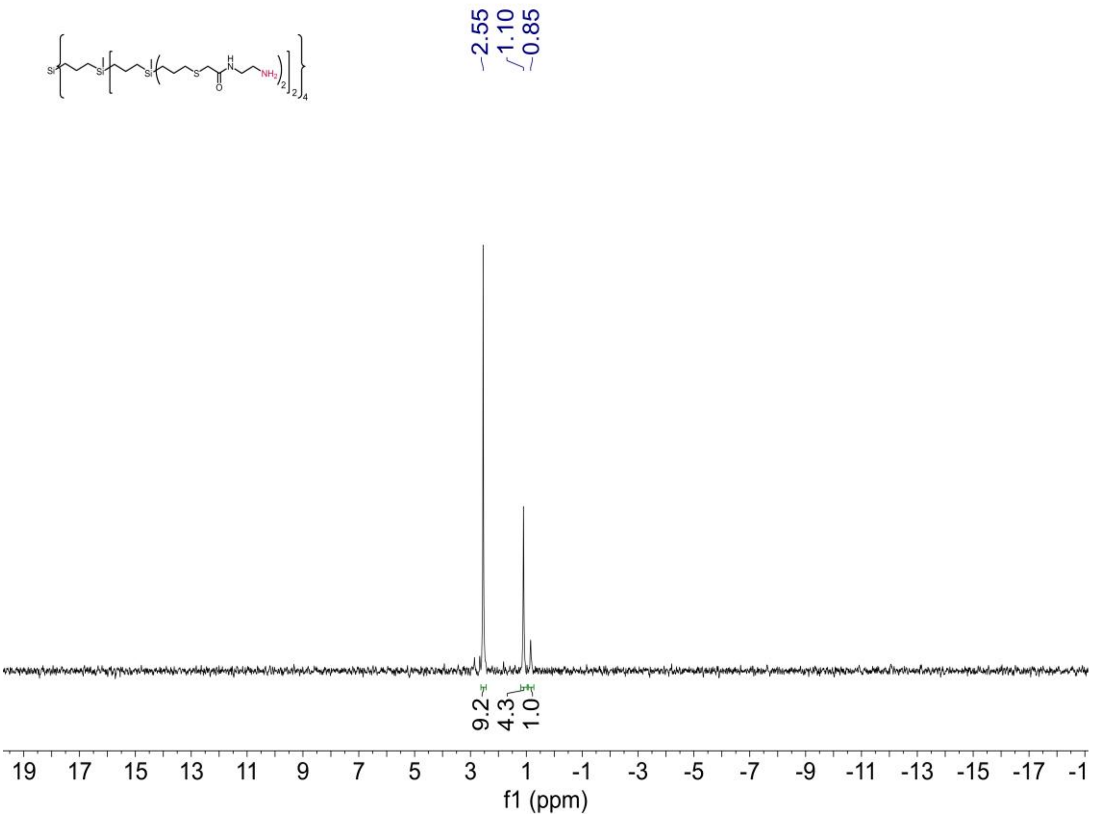

Figure S38: ^{29}Si {^{1}H} NMR (79 MHz, DMSO-d_6) **9**

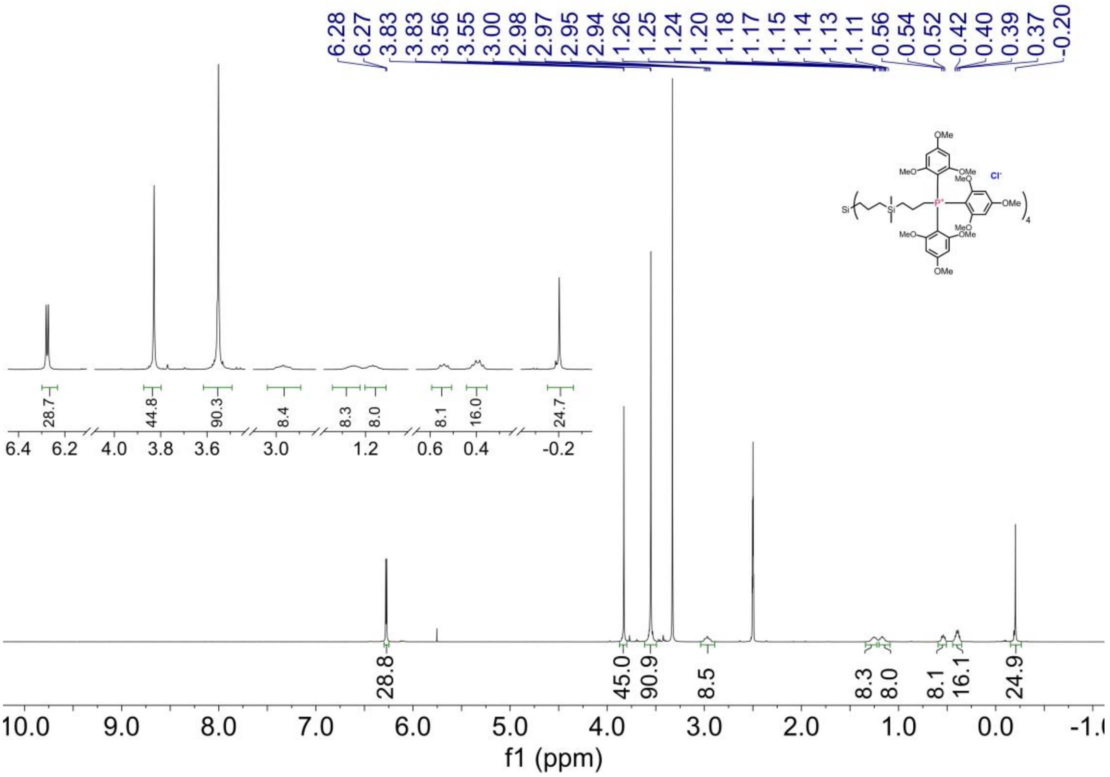

Figure S39: ^{1}H NMR (101 MHz, DMSO-d_6) **10b**

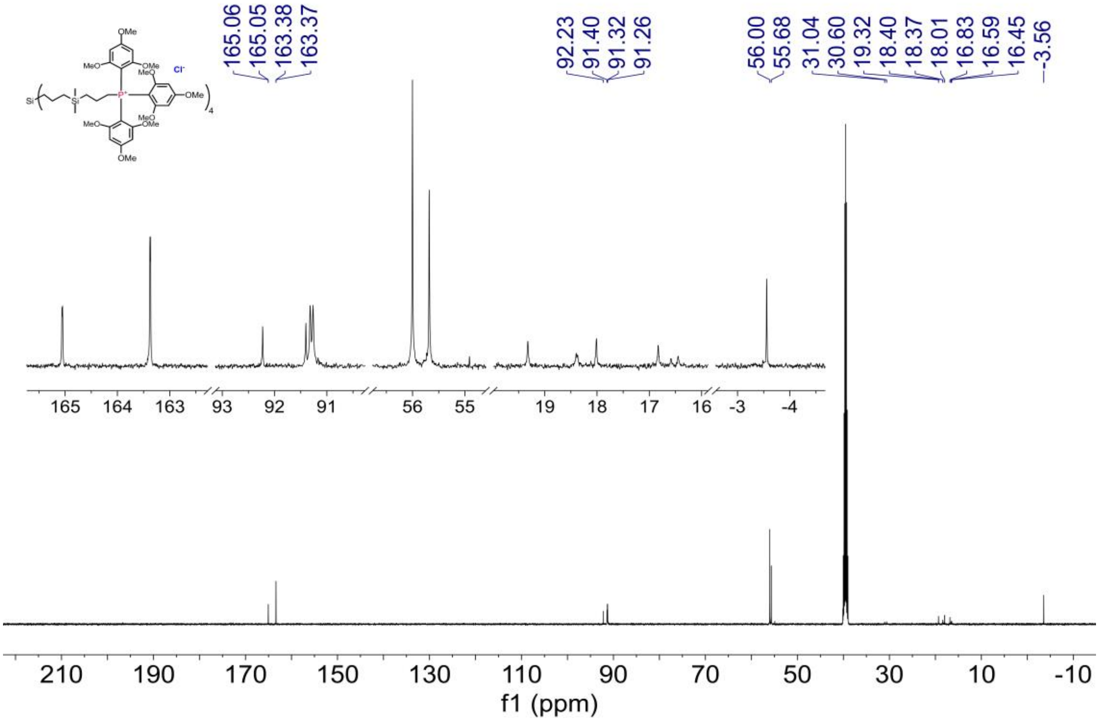

Figure S40: ^{13}C {^{1}H} NMR (101 MHz, DMSO-d_6) **10b**

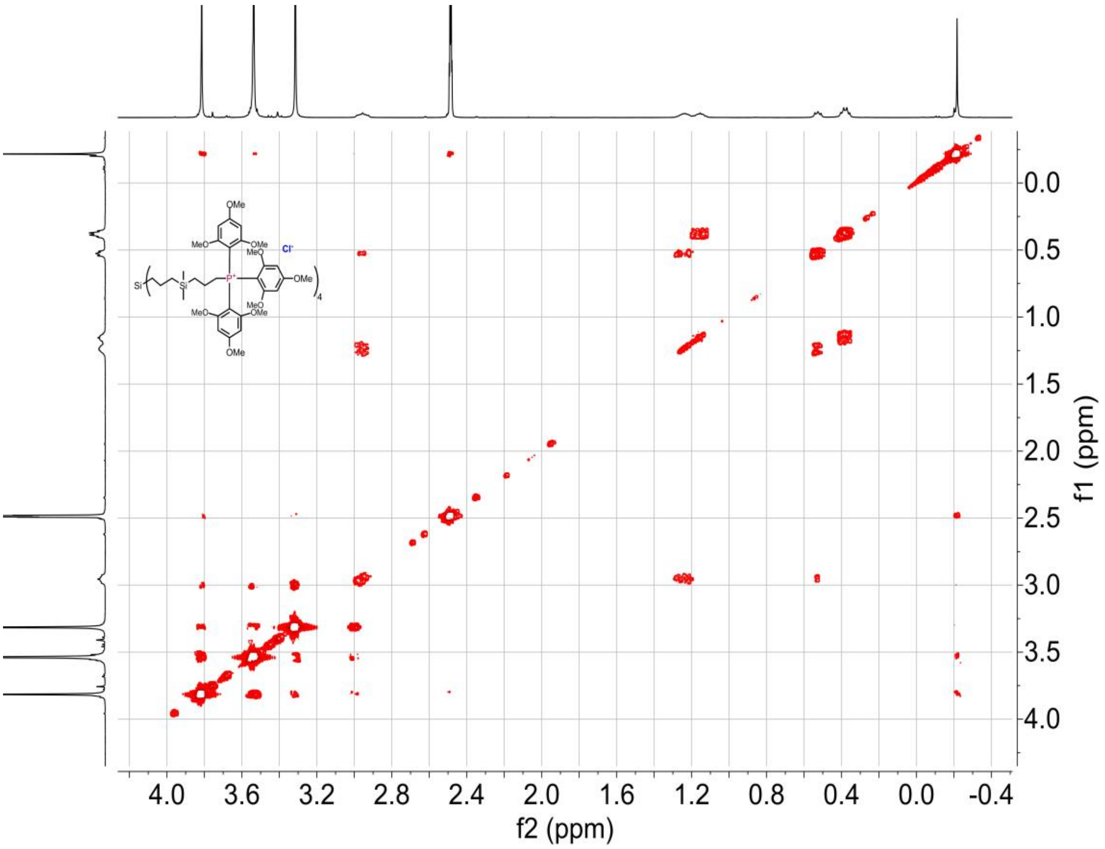

Figure S41: ^{1}H-^{1}H COSY NMR (DMSO-d_6) **10b**

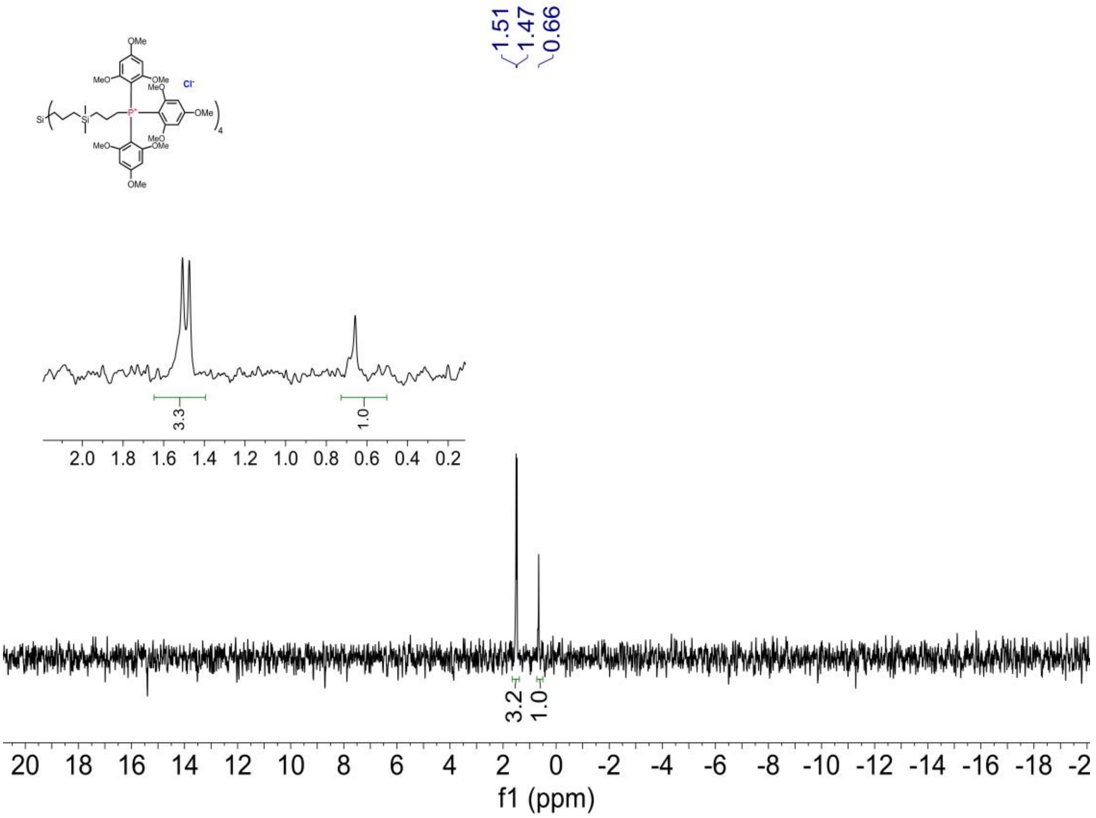

Figure S42: ^{29}Si {^{1}H} NMR (79 MHz, DMSO-d_6) **10b**

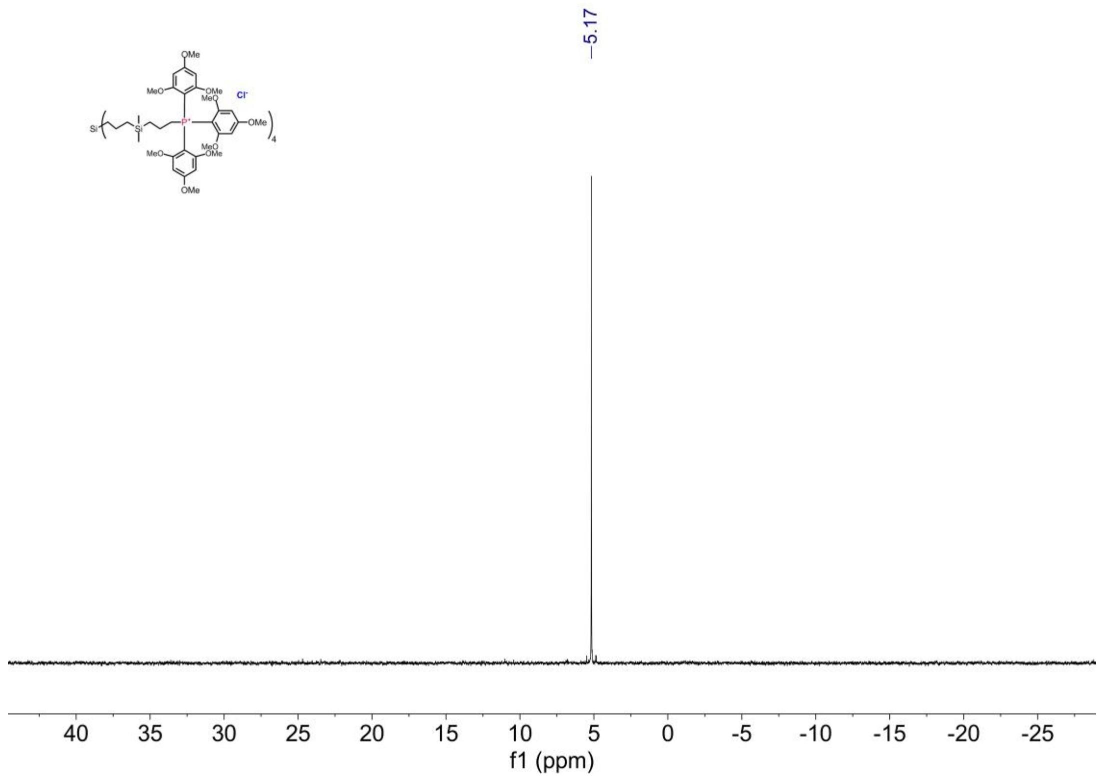

Figure S43: ^{31}P {^{1}H} NMR (162 MHz, DMSO-d_6) **10b**

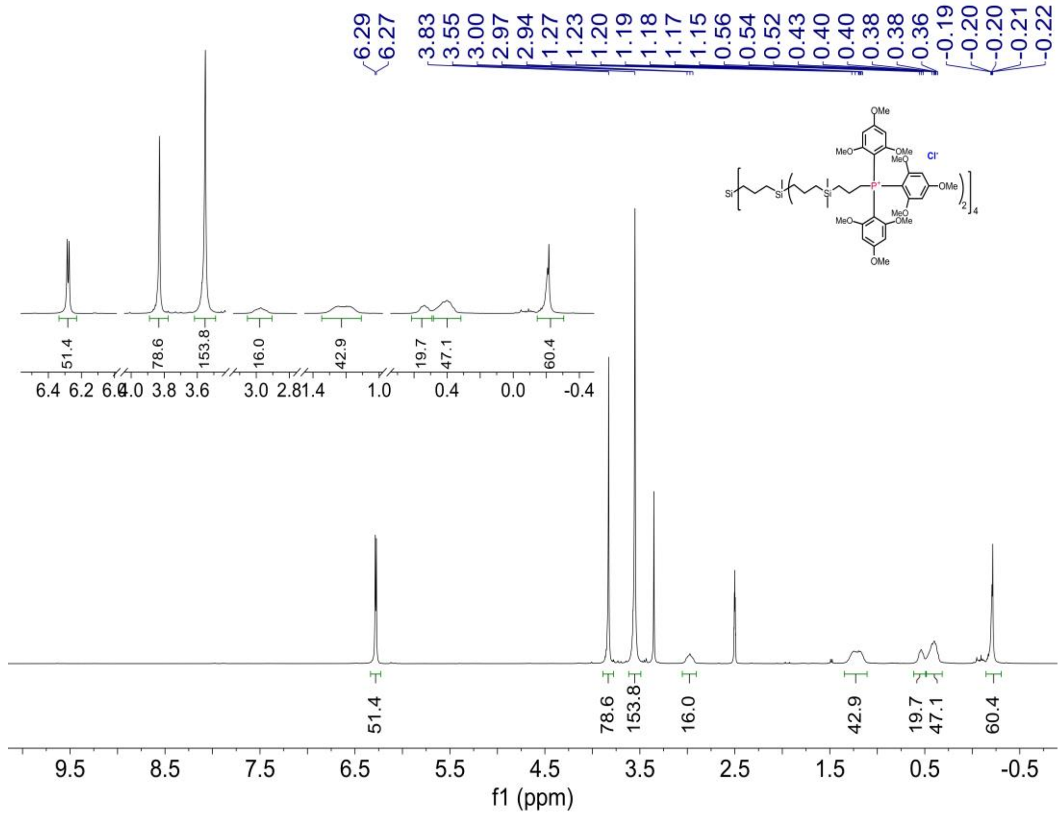

Figure S44: ^{1}H NMR (101 MHz, DMSO-d_6) **11b**

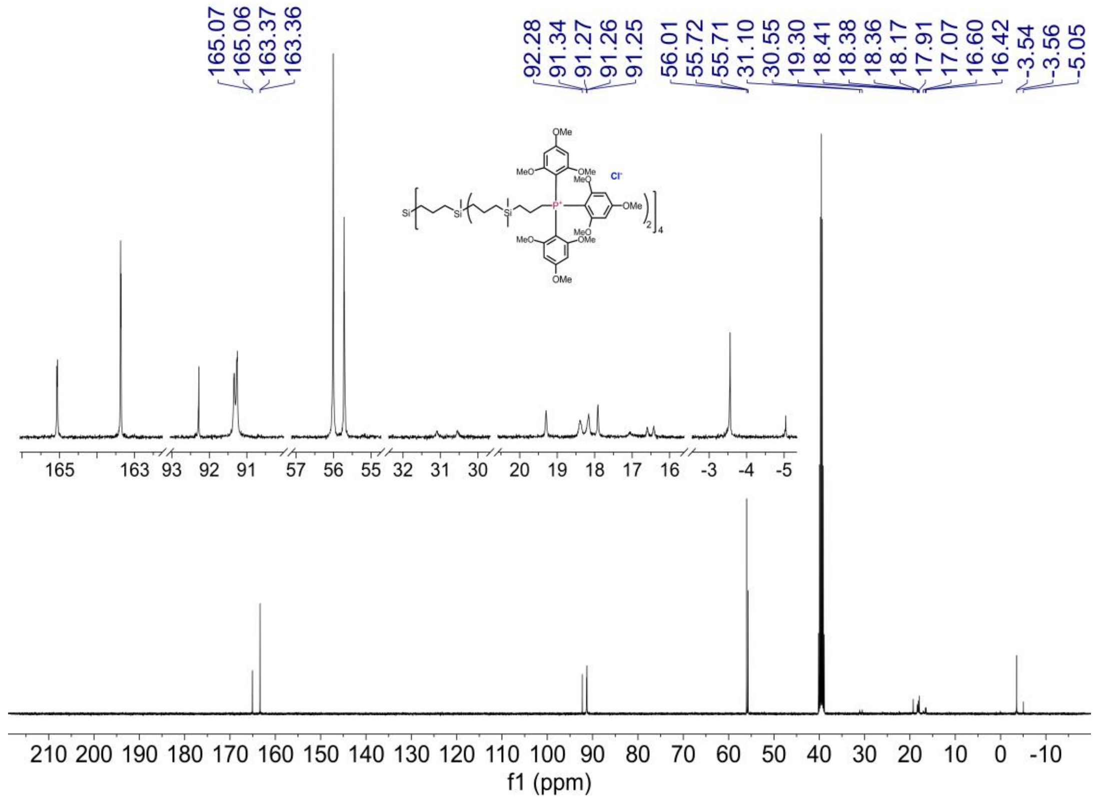

Figure S45: $^{13}C\ \{^{1}H\}$ NMR (101 MHz, DMSO-d_6) **11b**

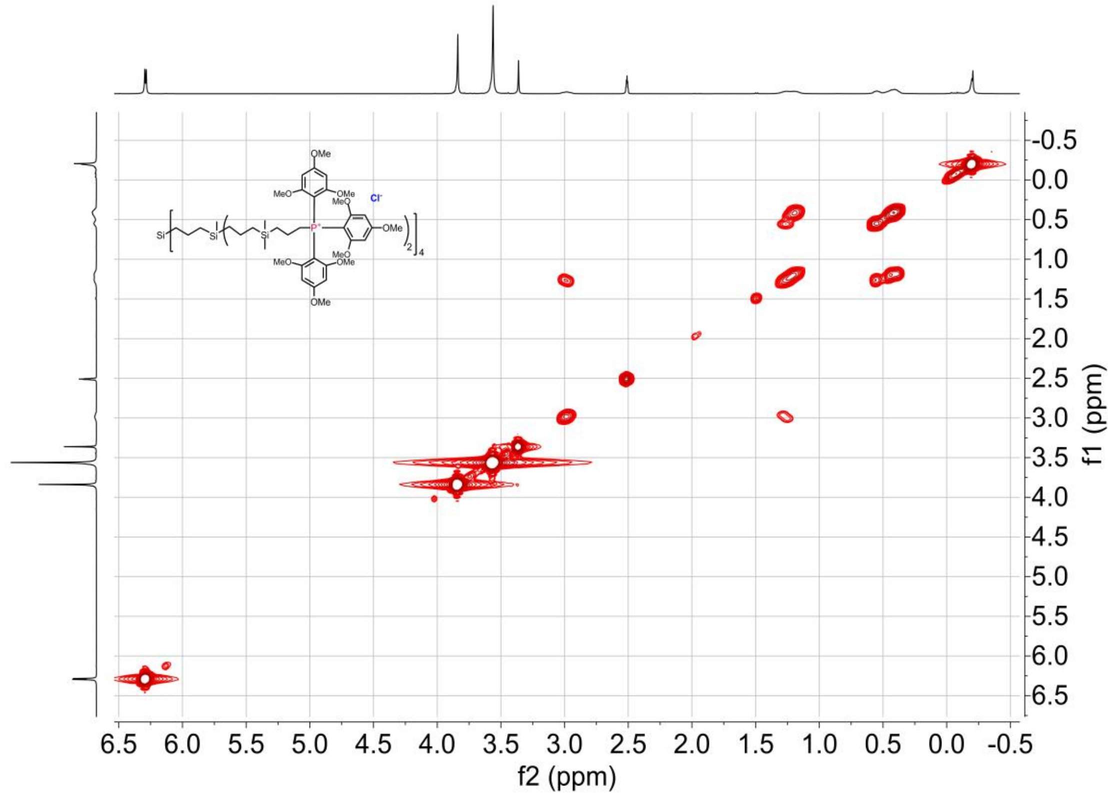

Figure S46: ^{1}H-^{1}H COSY NMR (DMSO-d_6) **11b**

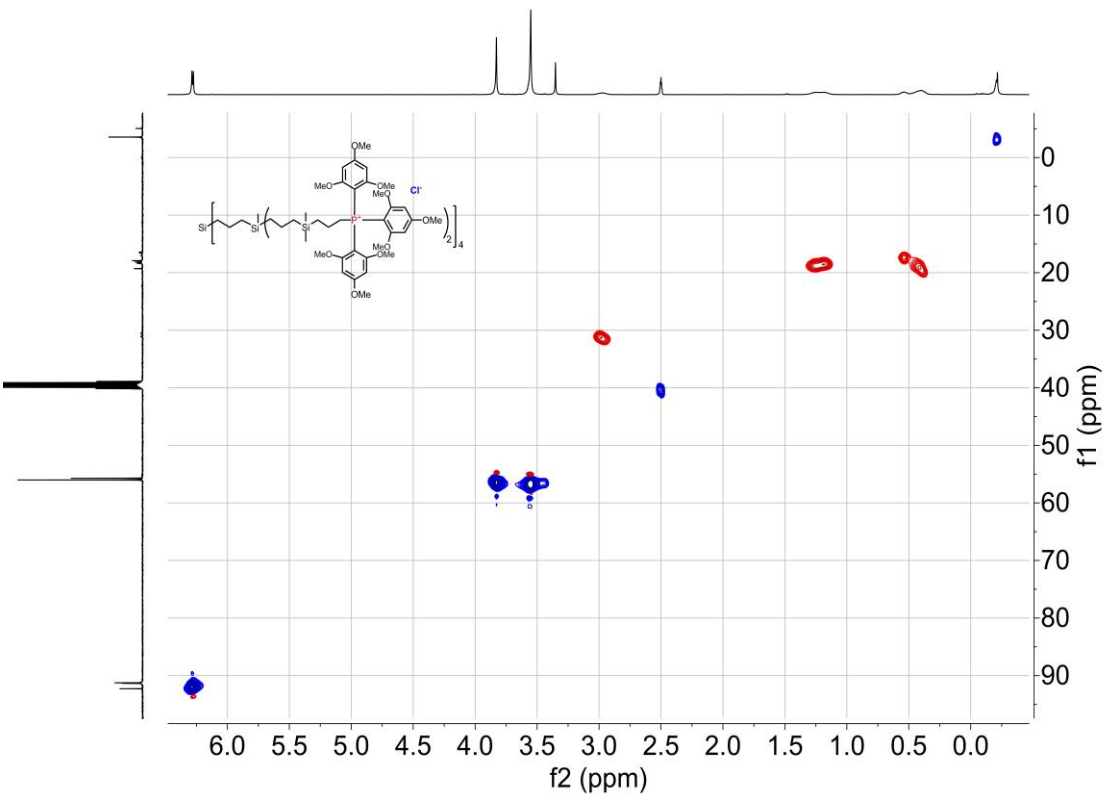

Figure S47: ^{1}H-^{13}C HSQC NMR (DMSO-d_6) **11b**

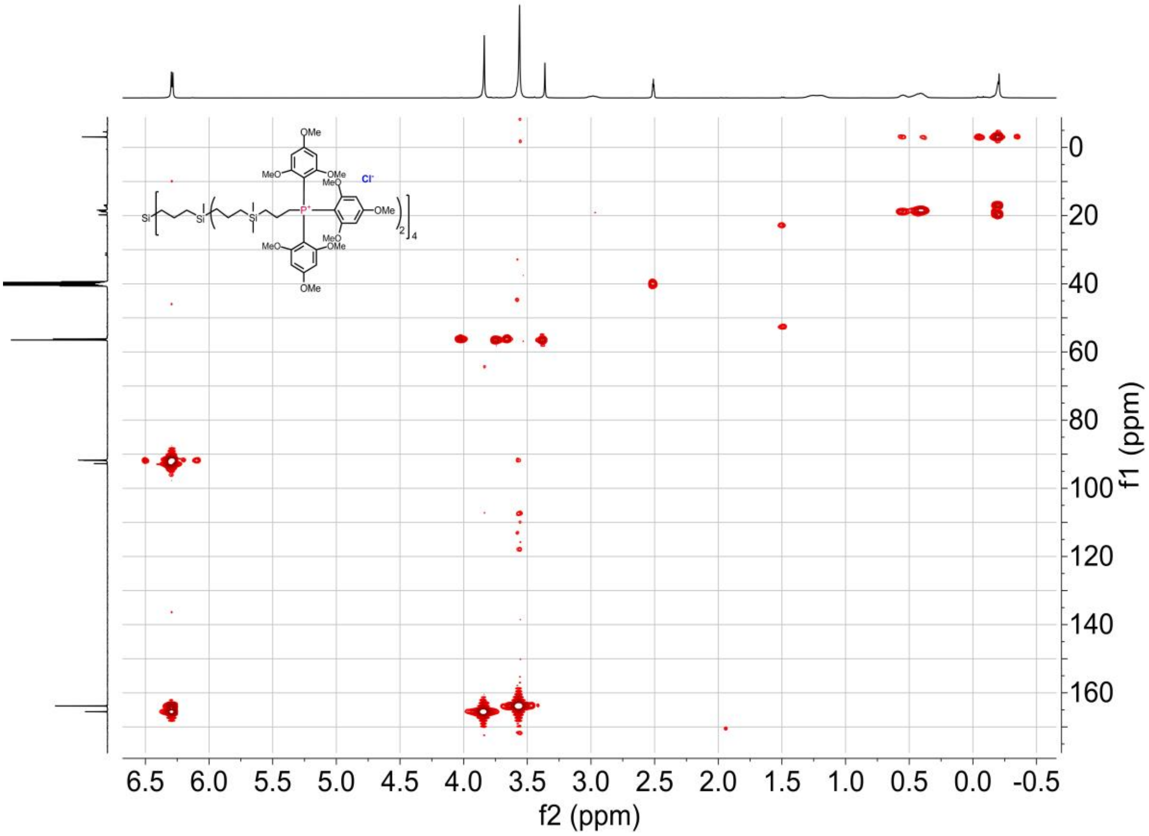

Figure S48: ^{1}H-^{13}C HMBC NMR (DMSO-d_6) **11b**

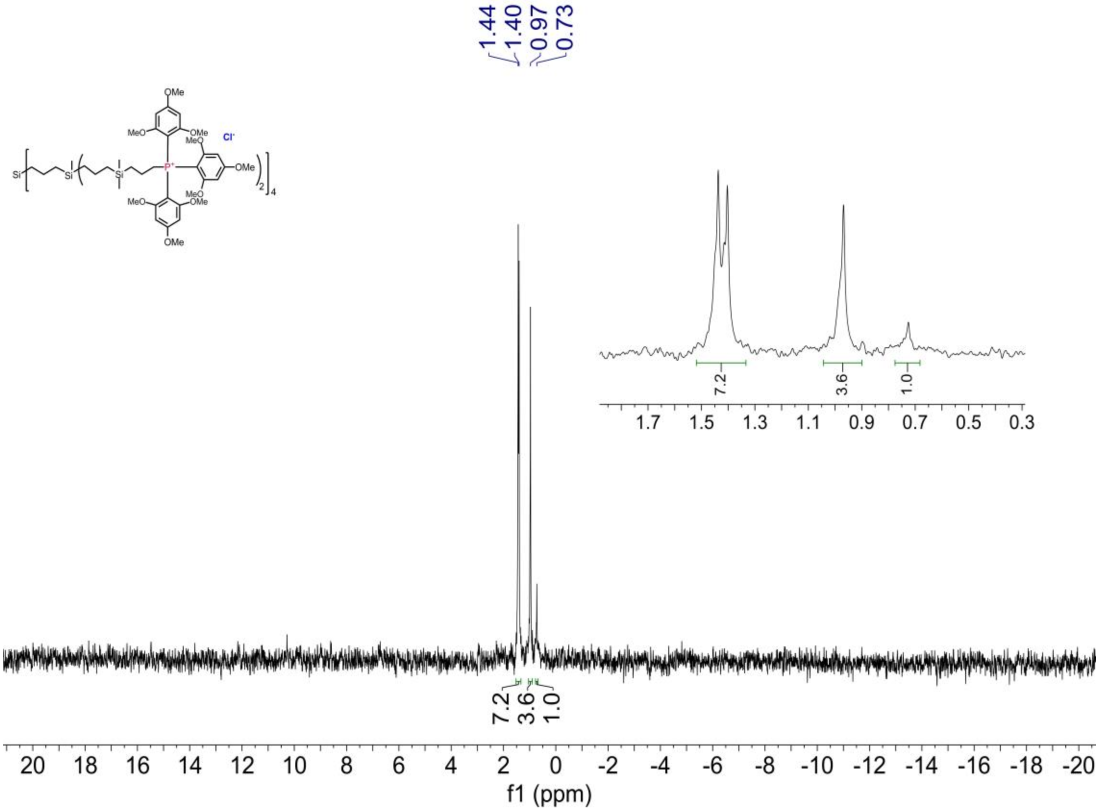

Figure S49: ^{29}Si {^{1}H} NMR (79 MHz, DMSO-d_6) **11b**

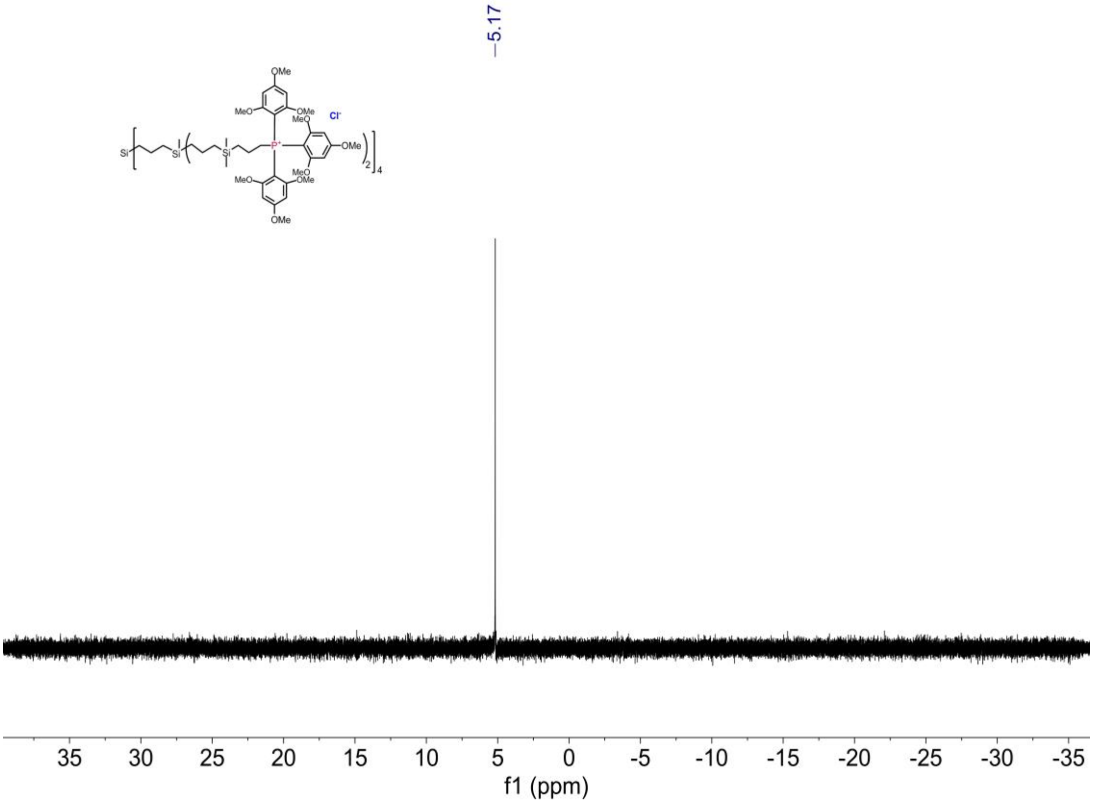

Figure S50: ^{31}P {^{1}H} NMR (162 MHz, DMSO-d_6) **11b**

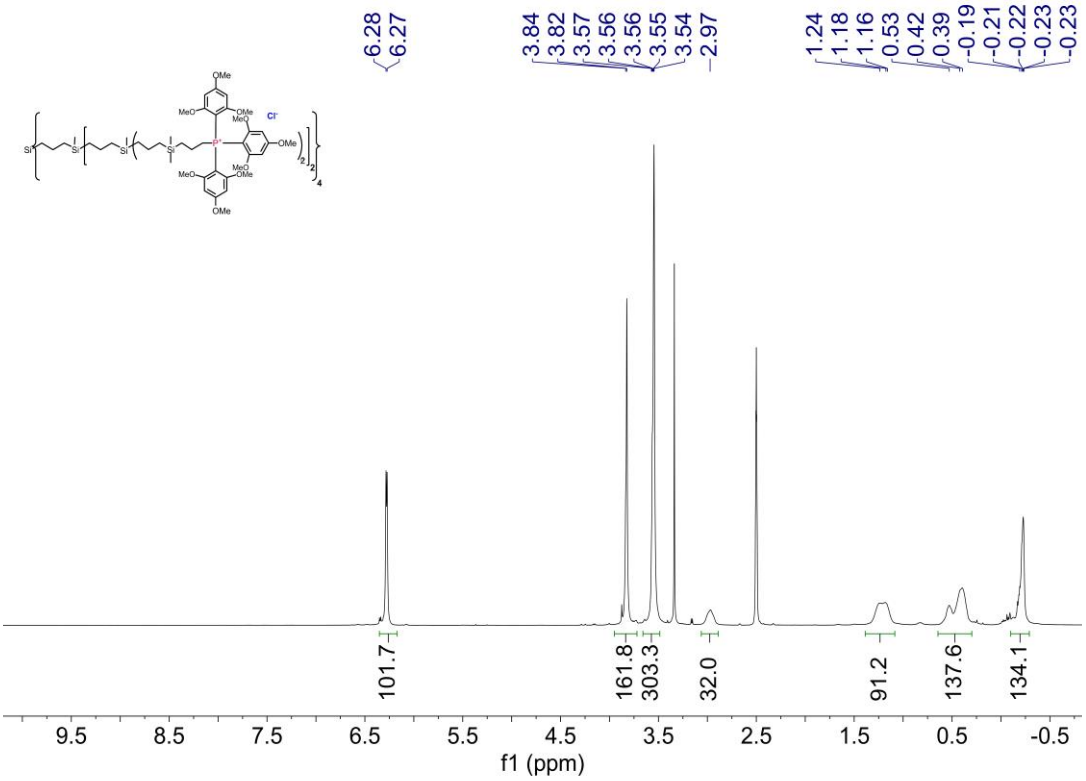

Figure S51: ^{1}H NMR (101 MHz, DMSO-d_6) **12b**

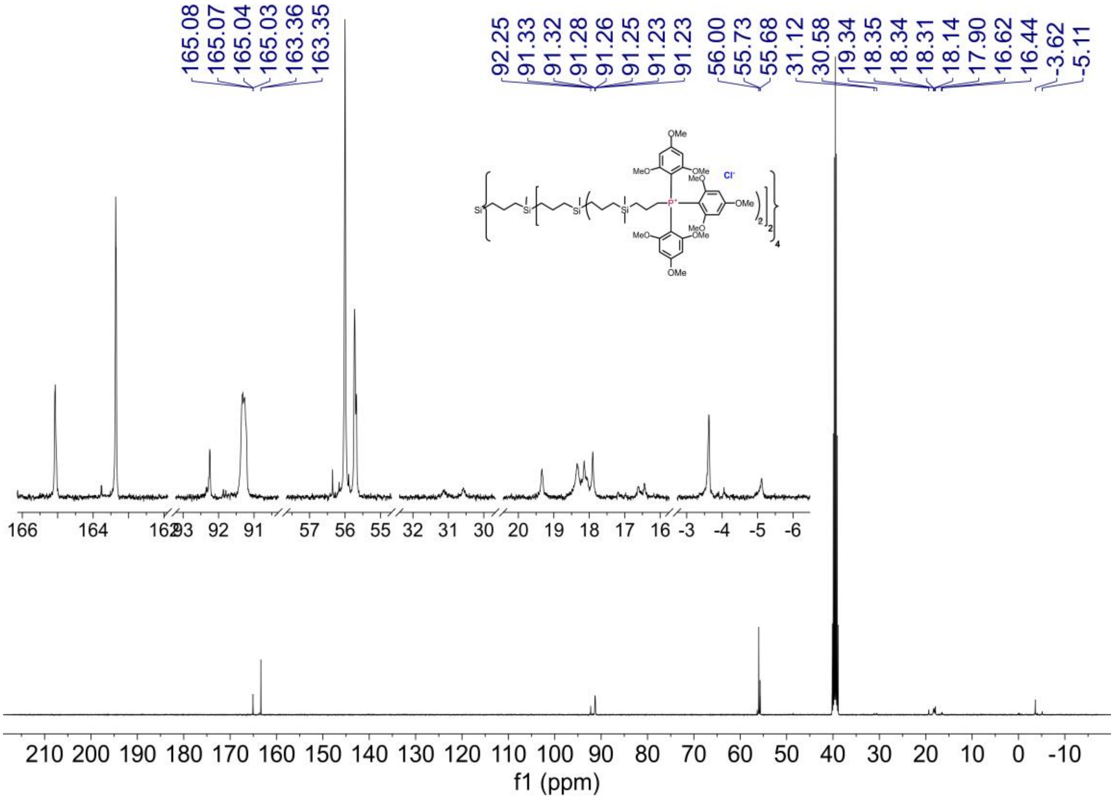

Figure S52: ^{13}C {^{1}H} NMR (101 MHz, DMSO-d_6) **12b**

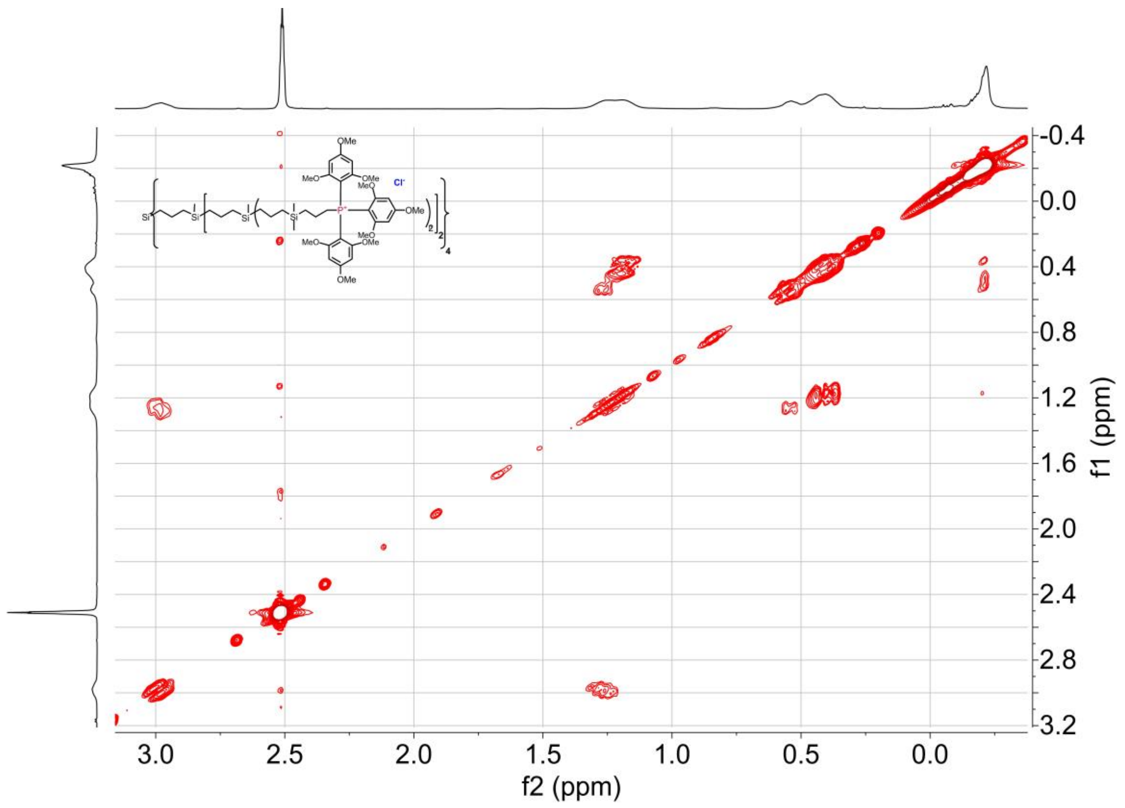

Figure S53: ^{1}H-^{1}H COSY NMR (DMSO-d_6) **12b**

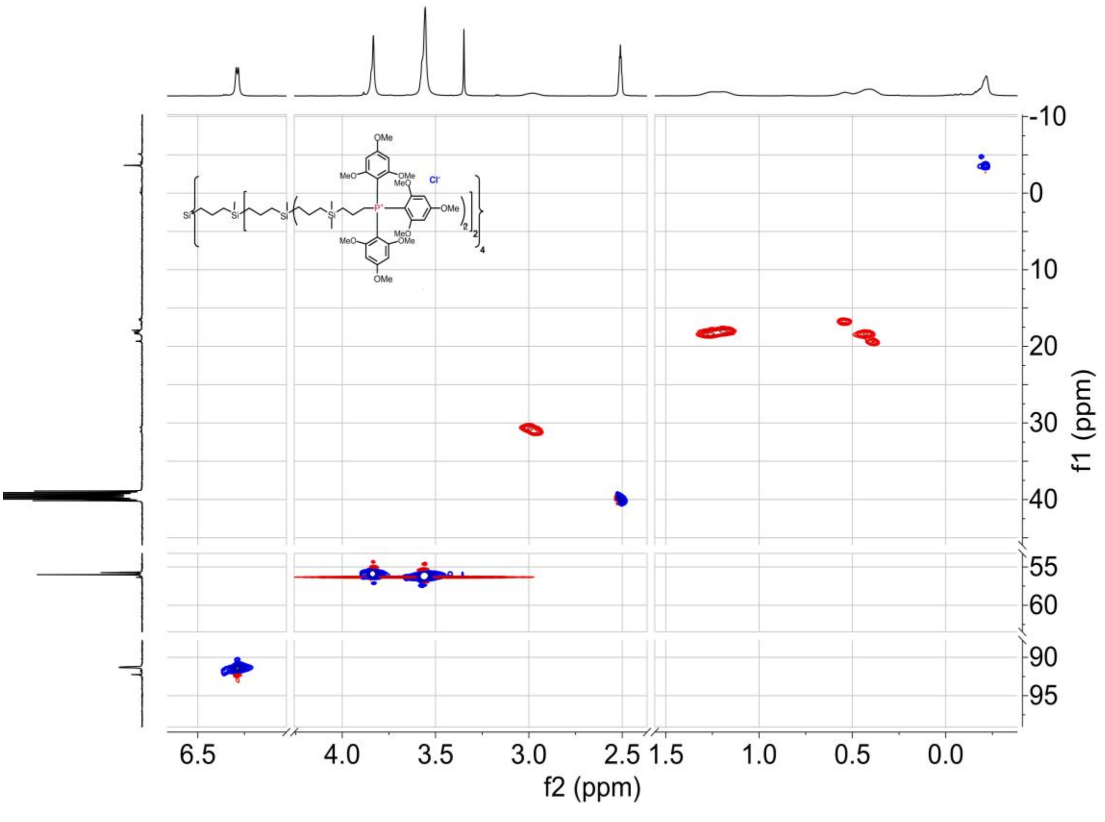

Figure S54: ^{1}H-^{13}C HSQC NMR (DMSO-d_6) **12b**

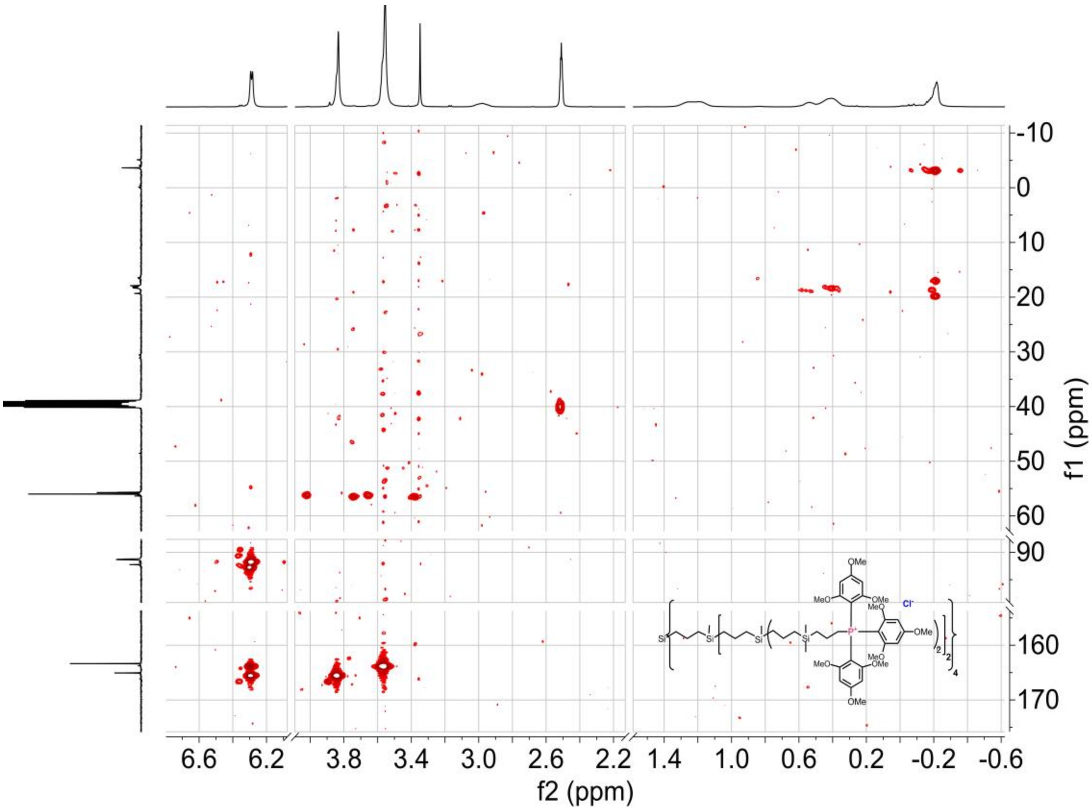

Figure S55: ^{1}H-^{13}C HMBC NMR (DMSO-d_6) **12b**

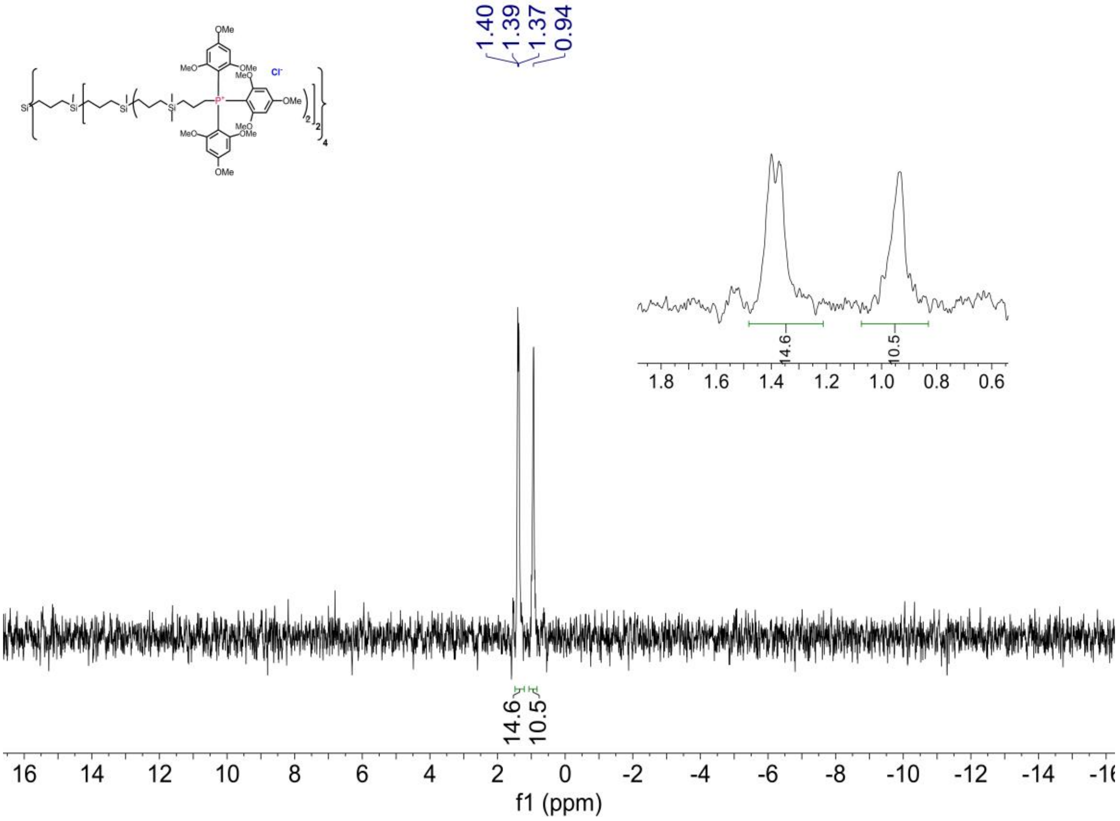

Figure S56: ^{29}Si {^{1}H} NMR (79 MHz, DMSO-d_6) **12b**

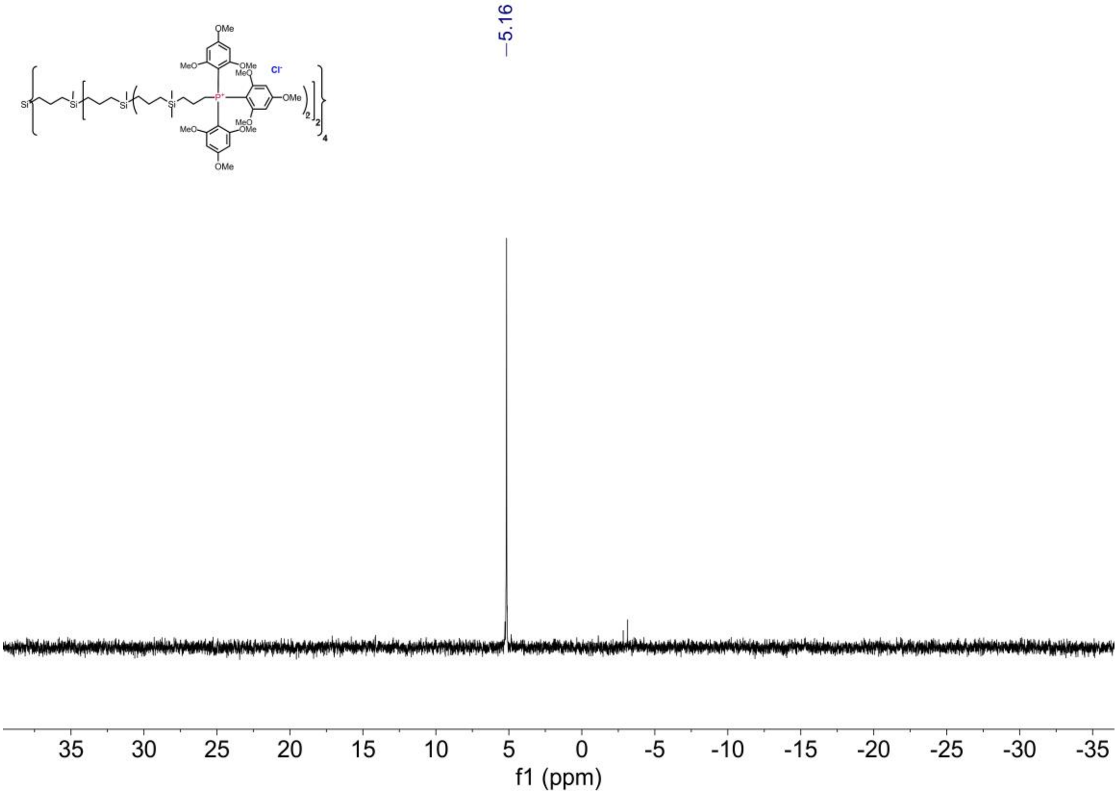

Figure S57: ^{31}P {^{1}H} NMR (162 MHz, DMSO-d_6) **12b**

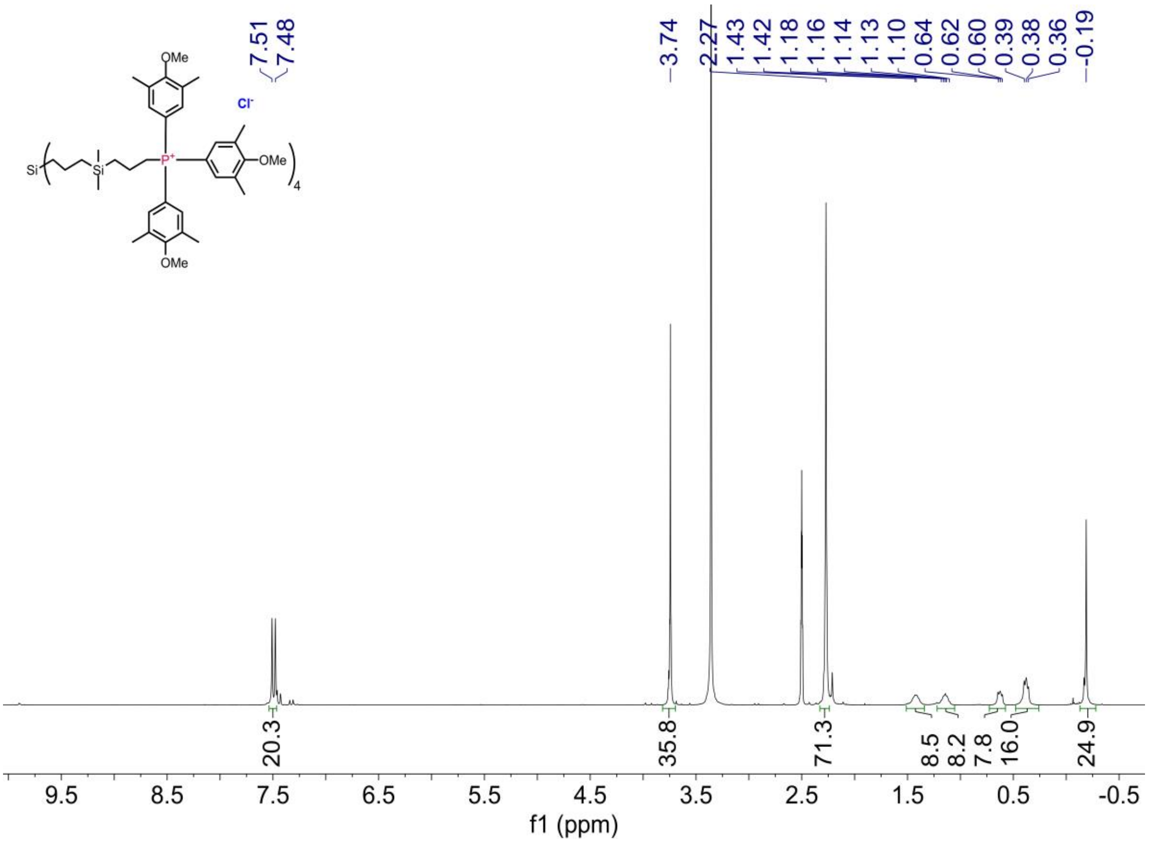

Figure S58: ^{1}H NMR (101 MHz, DMSO-d_6) **13b**

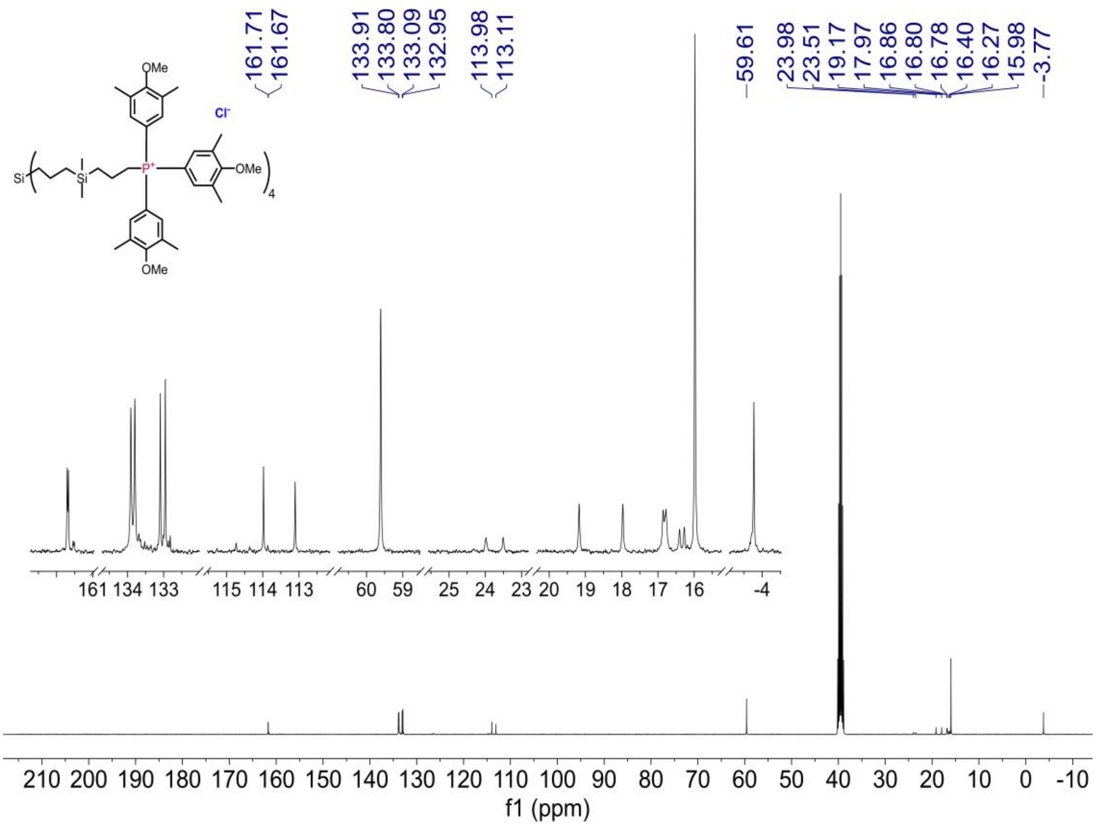

Figure S59: ^{13}C {^{1}H} NMR (101 MHz, DMSO-d_6) **13b**

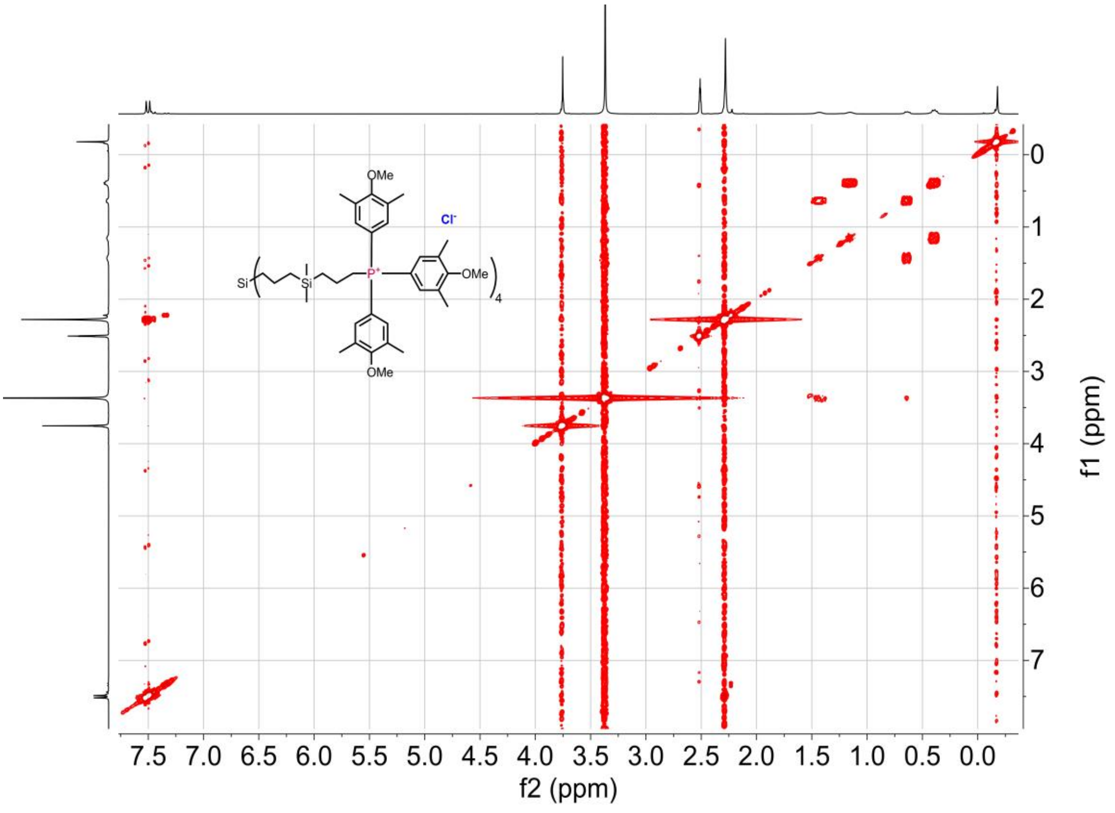

Figure S60: ^{1}H-^{1}H COSY NMR (DMSO-d_6) **13b**

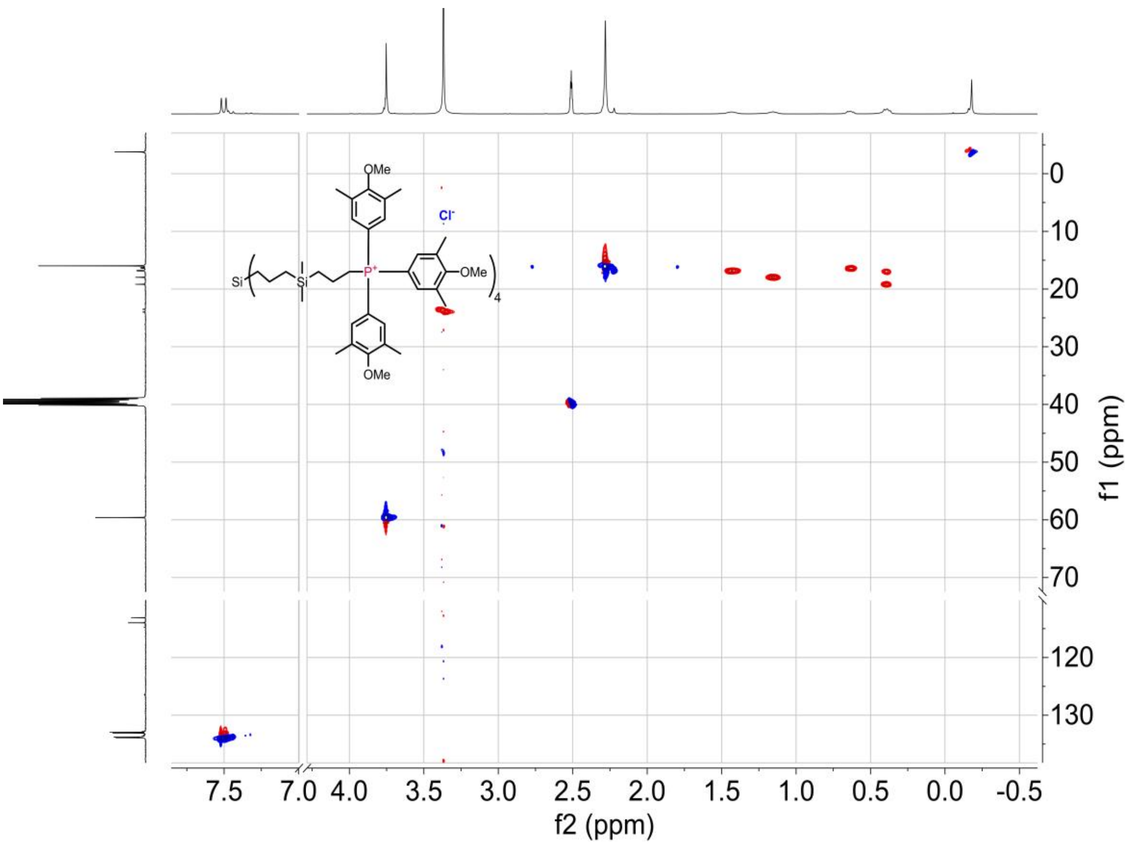

Figure S61: ^{1}H-^{13}C HSQC NMR (DMSO-d_6) **13b**

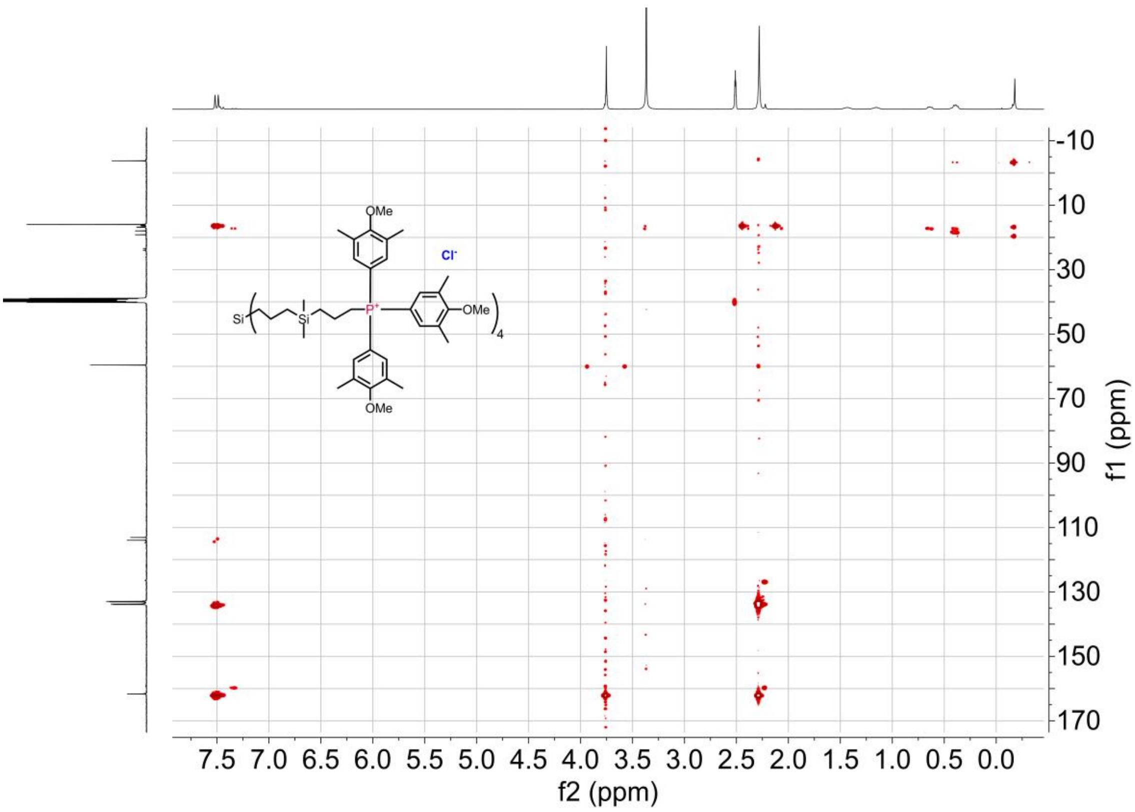

Figure S62: ^{1}H-^{13}C HMBC NMR (DMSO-d_6) **13b**

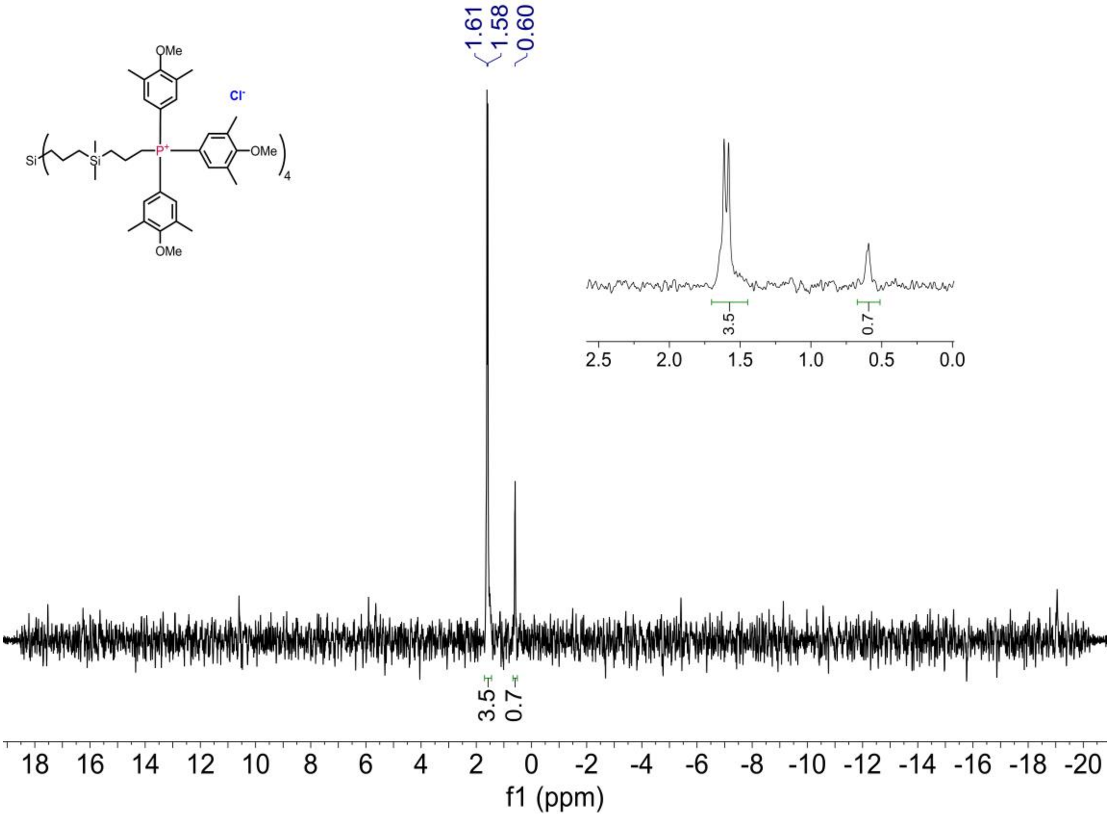

Figure S63: ^{29}Si {^{1}H} NMR (79 MHz, DMSO-d_6) **13b**

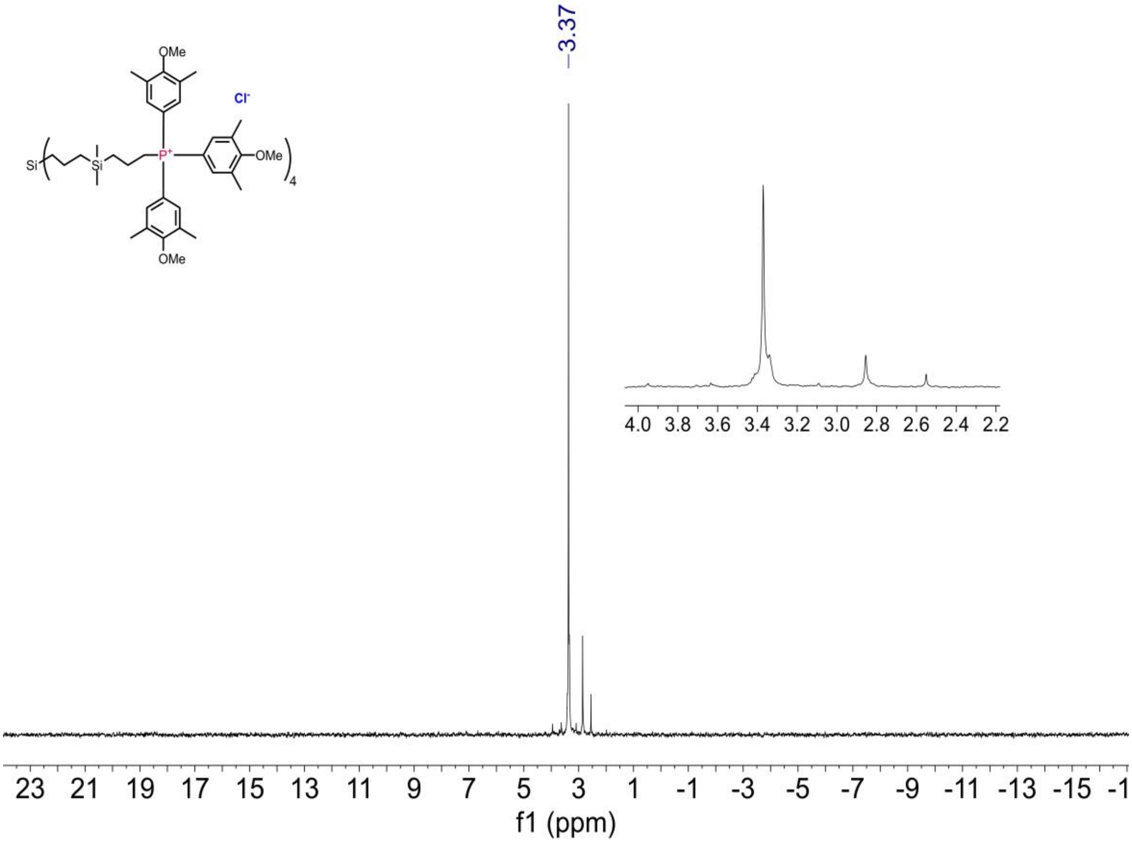

Figure S64: ^{31}P {^{1}H} NMR (162 MHz, DMSO-d_6) **13b**

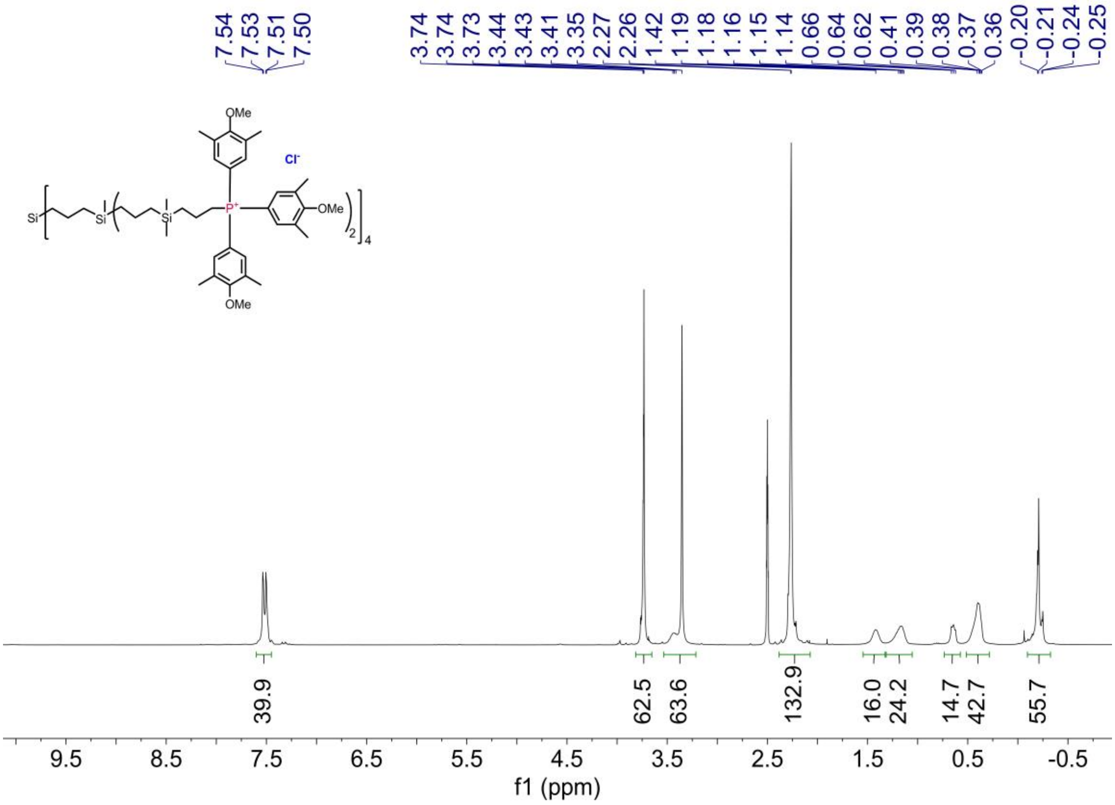

Figure S65: ^{1}H NMR (101 MHz, DMSO-d_6) **14b**

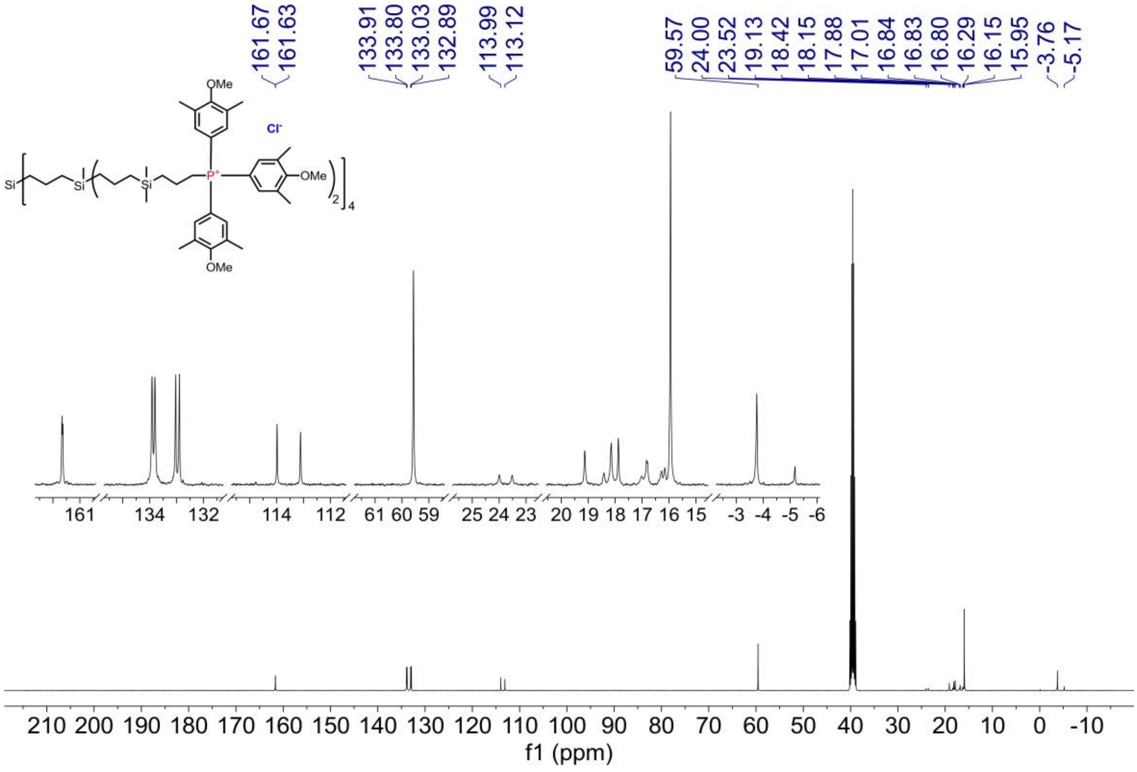

Figure S66: ^{13}C {^{1}H} NMR (101 MHz, DMSO-d_6) **14b**

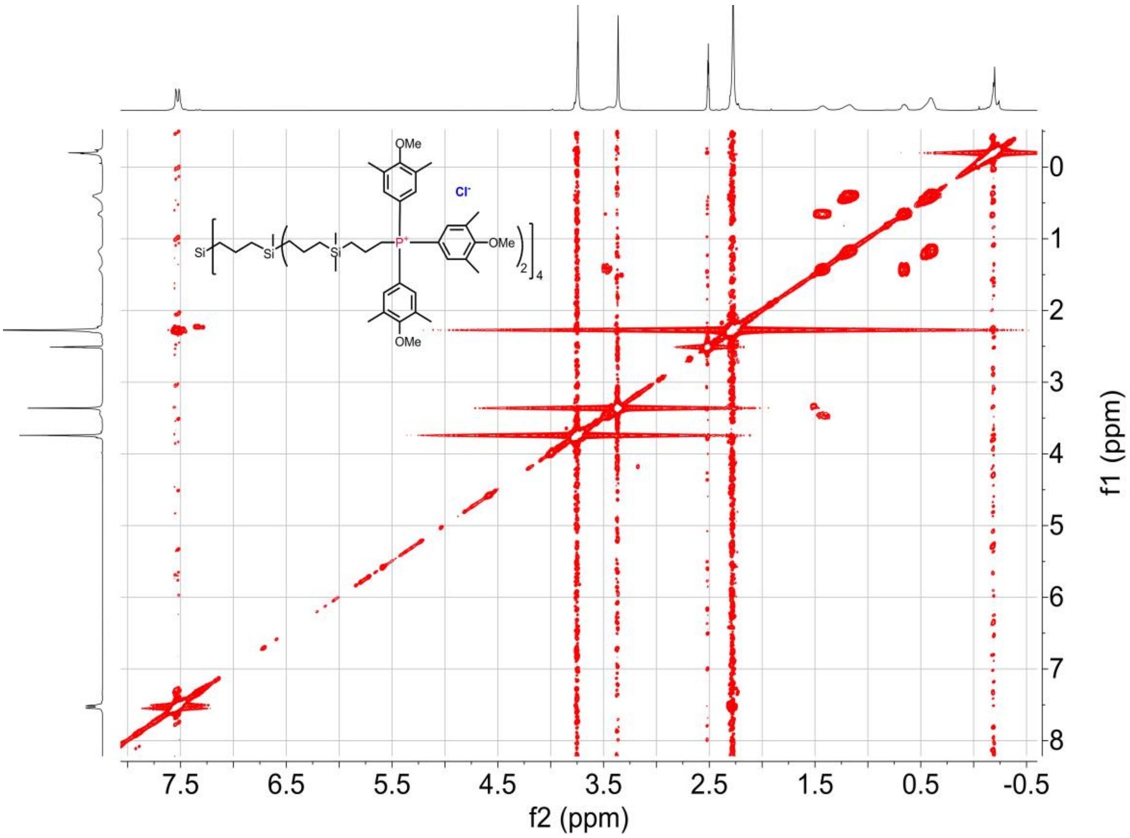

Figure S67: ^{1}H-^{1}H COSY NMR (DMSO-d_6) **14b**

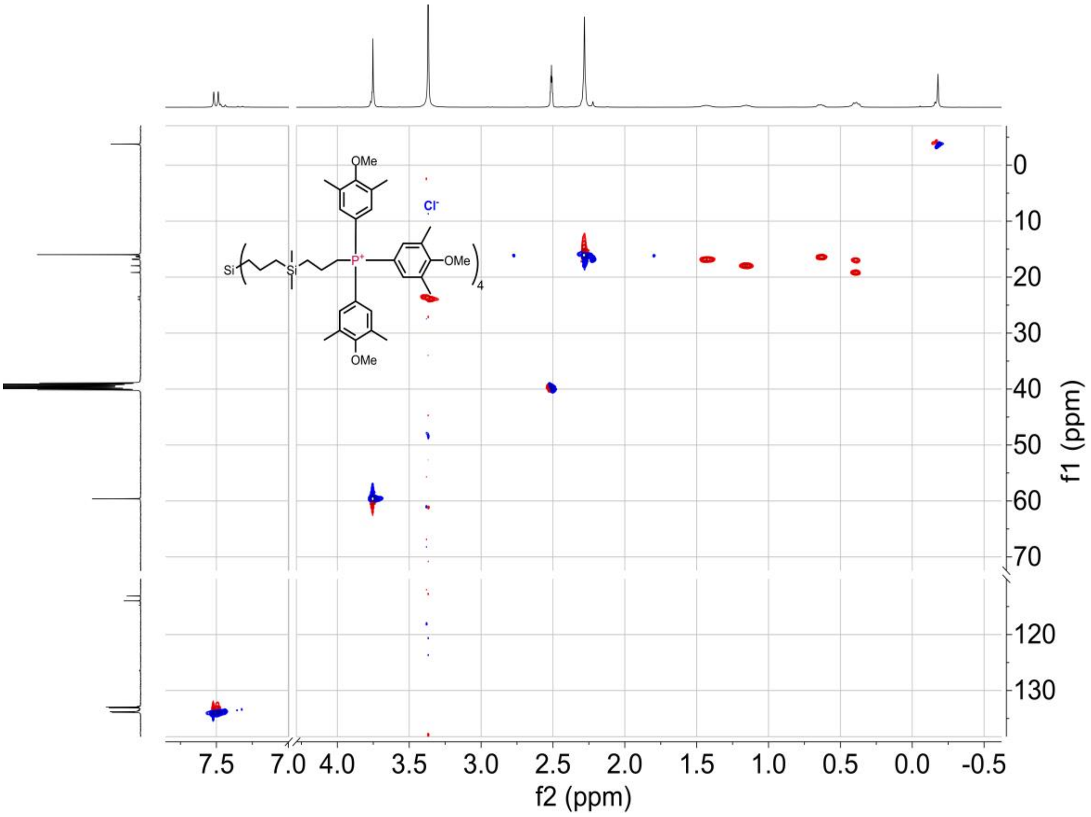

Figure S68: ^{1}H-^{13}C HSQC NMR (DMSO-d_6) **14b**

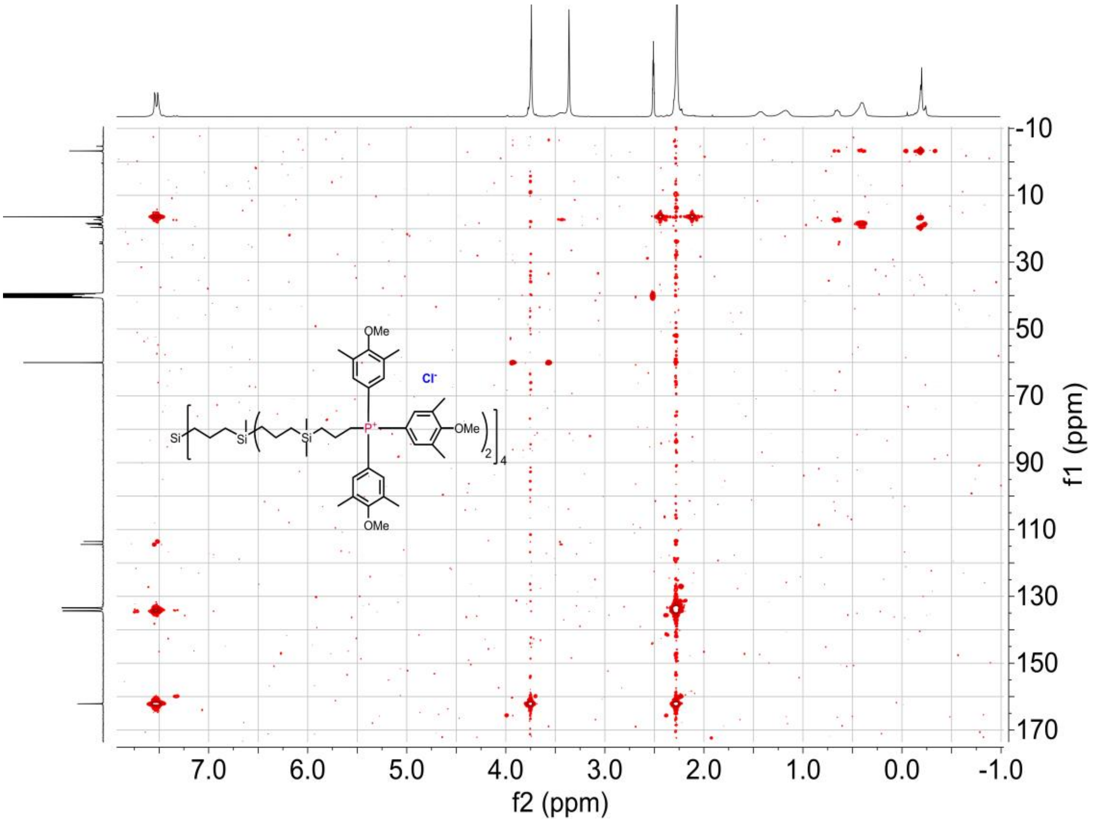

Figure S69: ^{1}H-^{13}C HMBC NMR (DMSO-d_6) **14b**

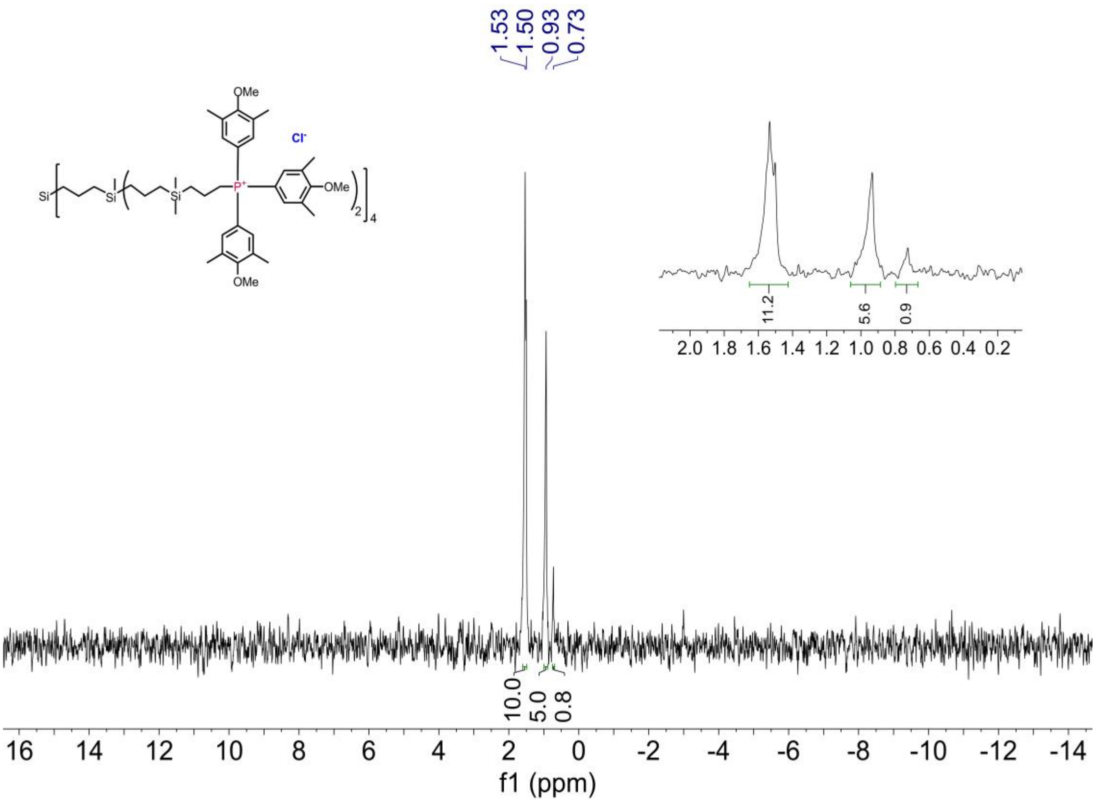

Figure S70: ^{29}Si {^{1}H} NMR (79 MHz, DMSO-d_6) **14b**

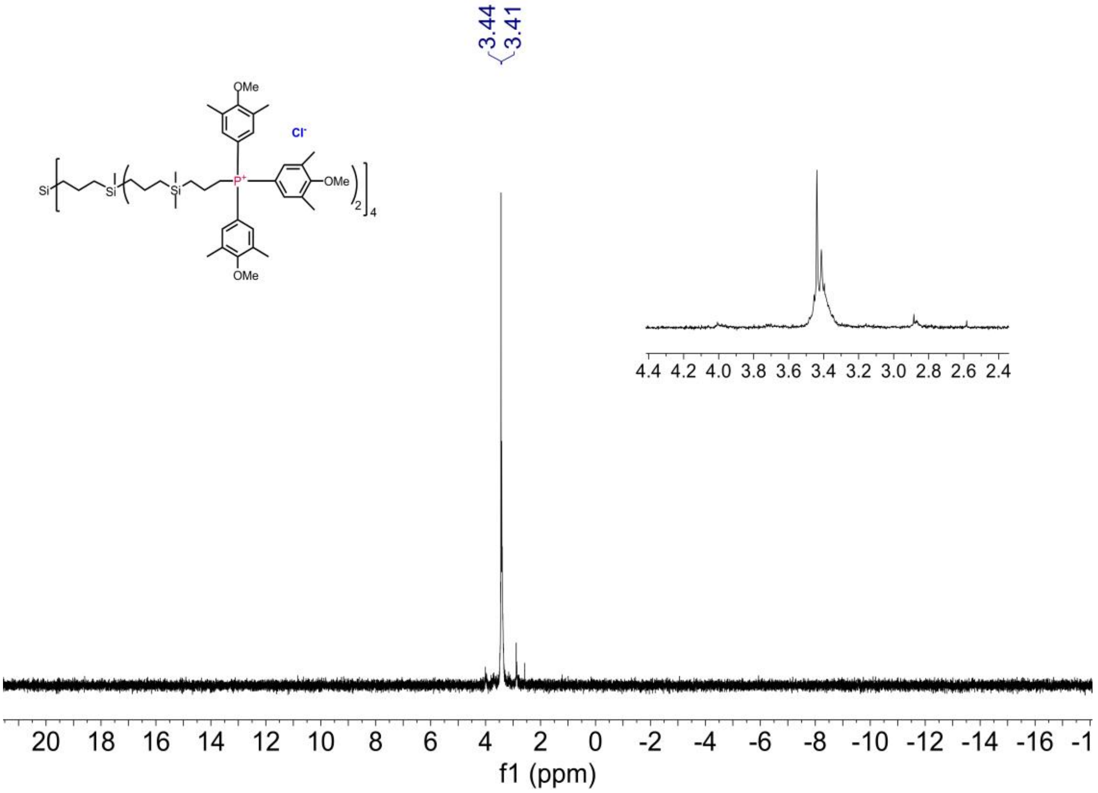

Figure S71: ^{31}P {^{1}H} NMR (162 MHz, DMSO-d_6) **14b**

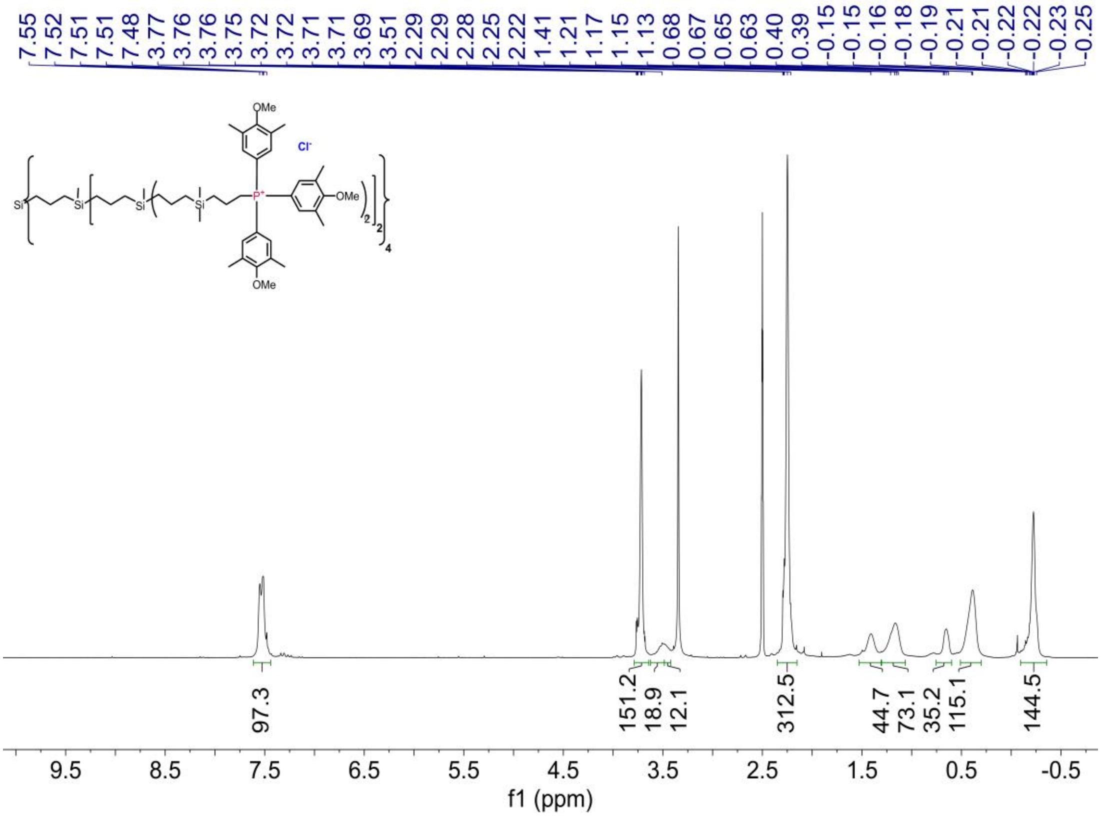

Figure S72: ^{1}H NMR (101 MHz, DMSO-d_6) **15b**

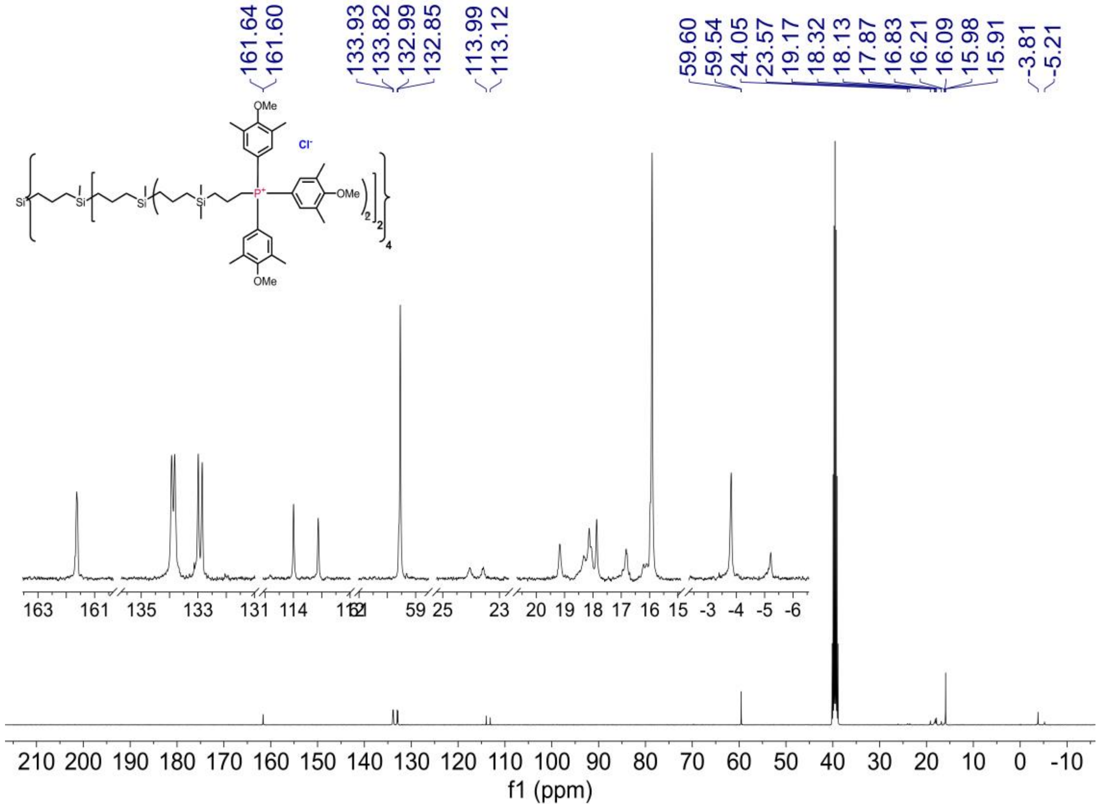

Figure S73: ^{13}C {^{1}H} NMR (101 MHz, DMSO-d_6) **15b**

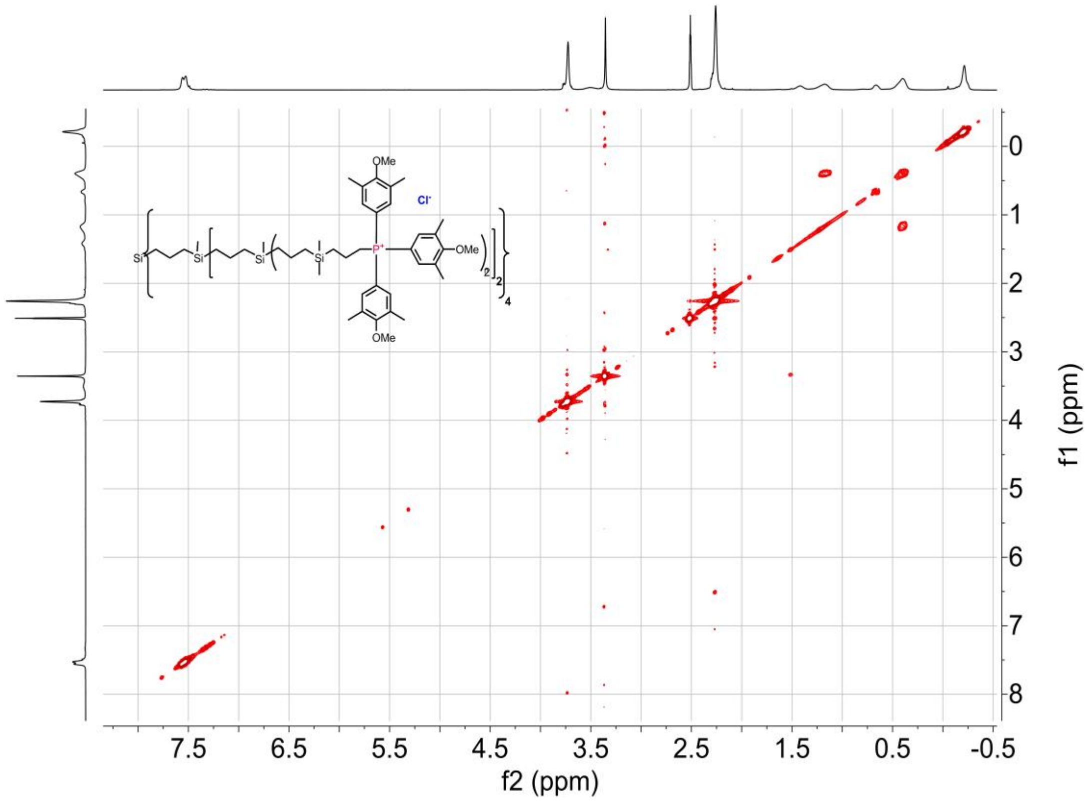

Figure S74: ^{1}H-^{1}H COSY NMR (DMSO-d_6) **15b**

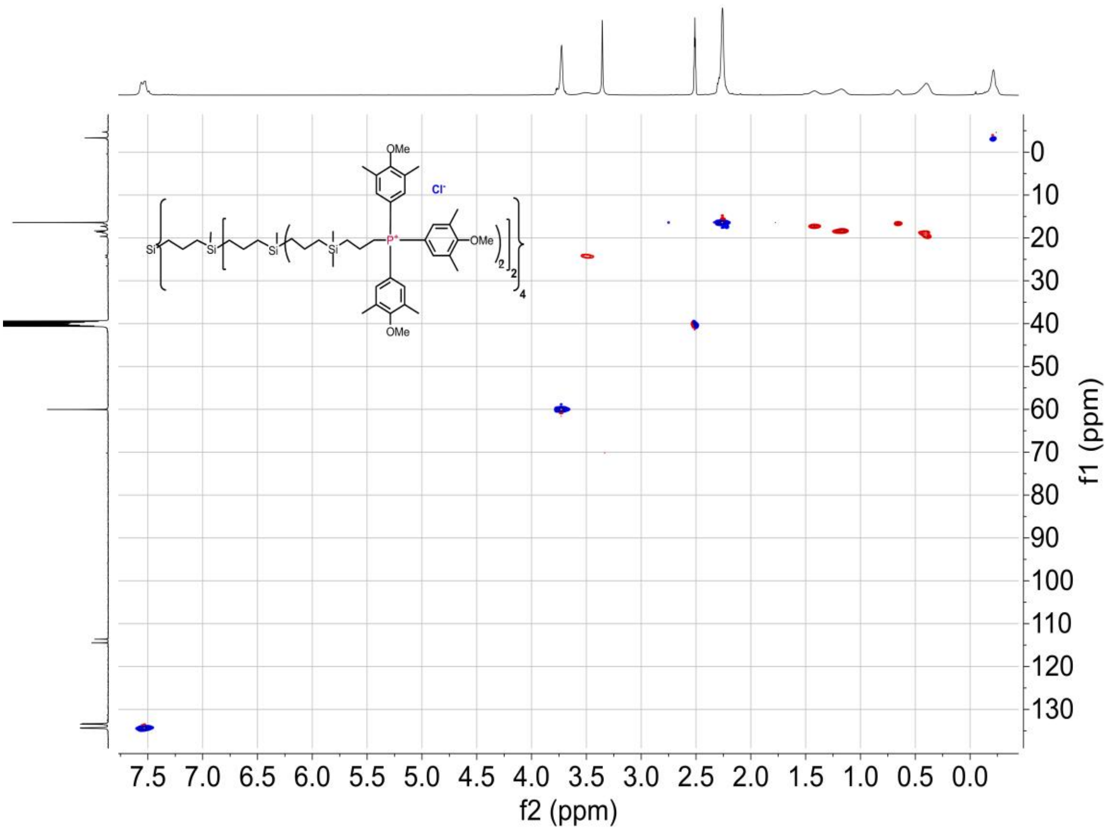

Figure S75: ^{1}H-^{13}C HSQC NMR (DMSO-d_6) **15b**

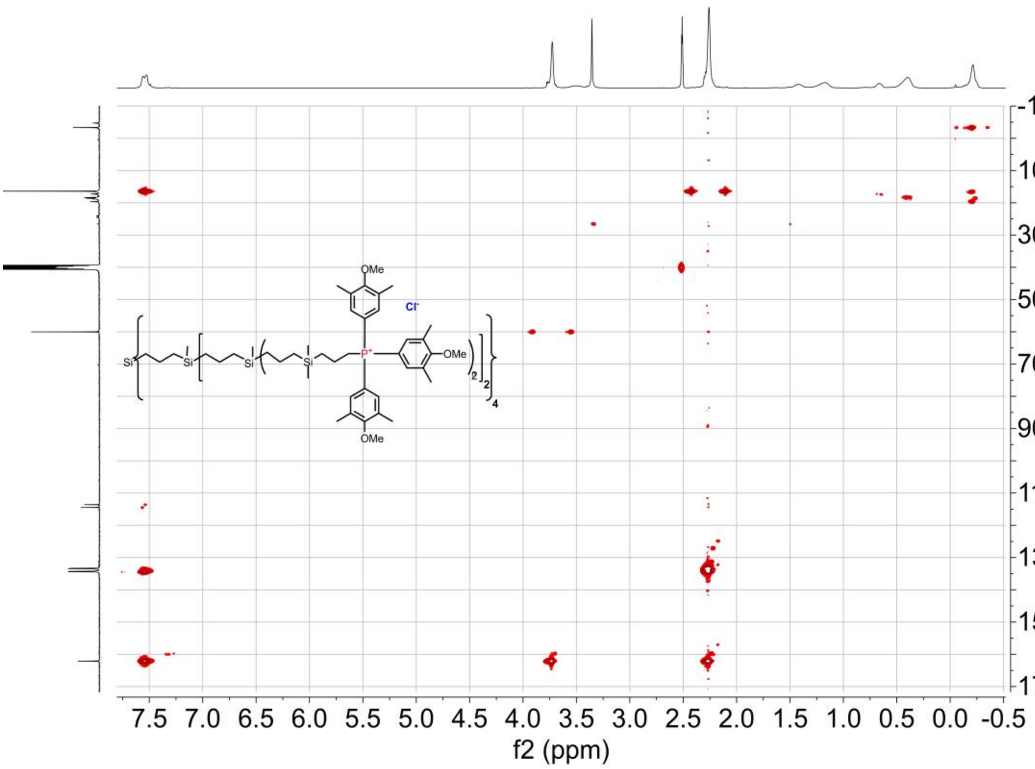

Figure S76: ^{1}H-^{13}C HMBC NMR (DMSO-d_6) **15b**

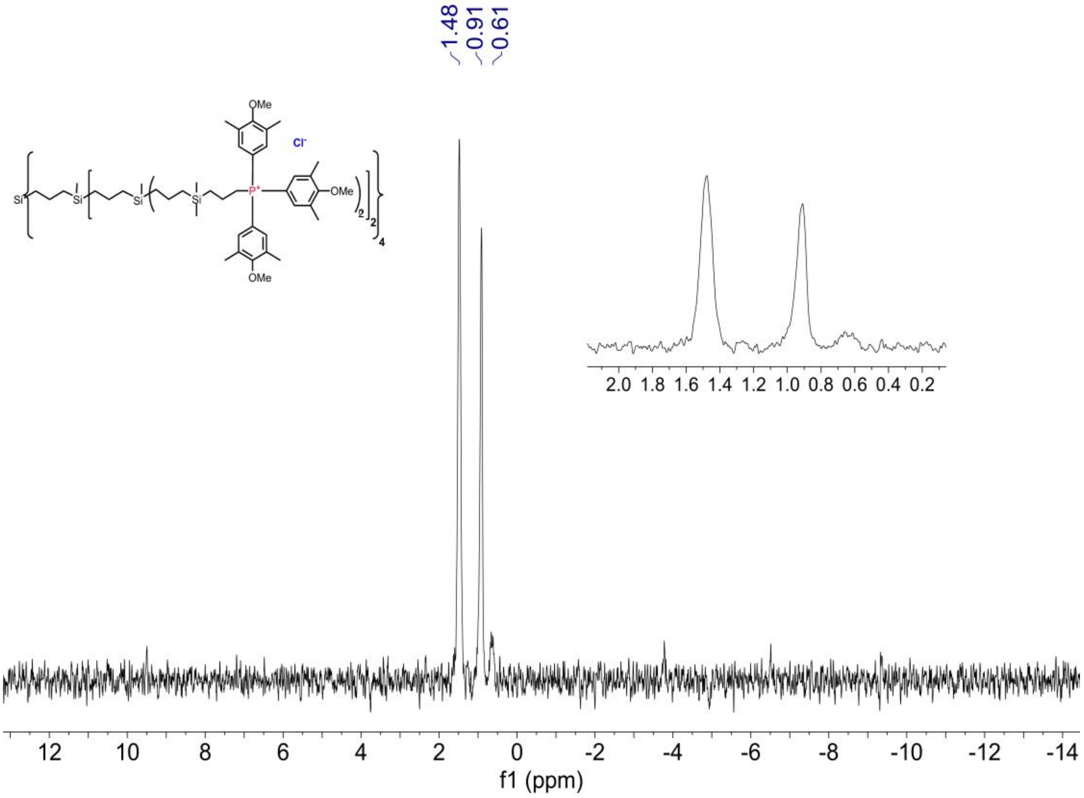

Figure S77: ^{29}Si {^{1}H} NMR (79 MHz, DMSO-d_6) **15b**

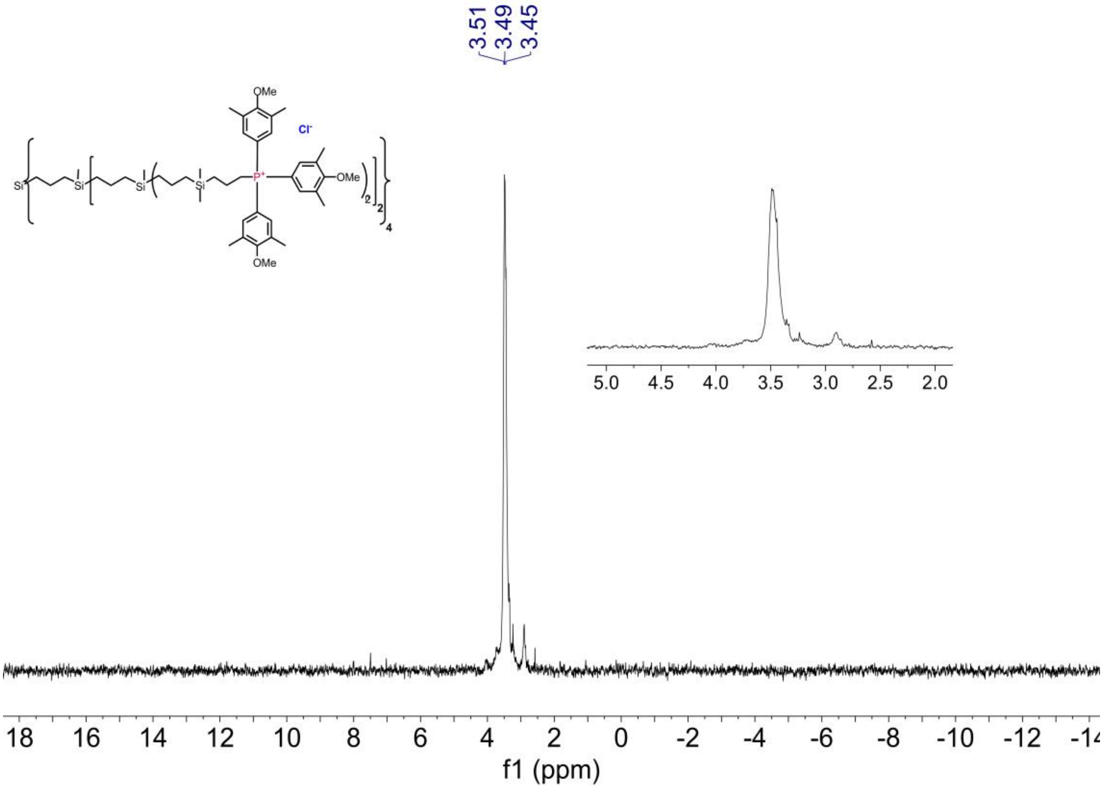

Figure S78: ^{31}P {^{1}H} NMR (162 MHz, DMSO-d_6) **15b**
